# Small gains, large losses: range shifts of the hoverfly *Dioprosopa clavata* (Fabricius, 1794) (Diptera: Syrphidae) to 2100

**DOI:** 10.1007/s00484-026-03147-y

**Published:** 2026-02-24

**Authors:** Janderson Batista Rodrigues Alencar, João Paulo Nunes, Matheus Augusto do Nascimento, Alessandre Pereira-Colavite, Adeilson de Melo Silva, Clarissa Rosa

**Affiliations:** 1 Programa Institucional de Pós-Doutorado, Pós-graduação em Ecologia, Instituto Nacional de Pesquisas da Amazônia, Constelação Cruzeiro do Sul Street, Aleixo, Manaus, Amazonas 69060-062 Brazil; 2 Laboratório de Entomologia, Departamento de Sistemática e Ecologia, CCEN, Universidade Federal da Paraíba, João Pessoa, Paraíba Brazil; 3 Núcleo de Ecologia e Monitoramento Ambiental (NEMA), Universidade Federal do Vale do São Francisco, Rodovia BR-407, KM 12 Lote 543 - S/N C1, Petrolina, Pernambuco 56300-000 Brazil; 4 Coordenação de Dinâmica Ambiental (CODAM), Instituto Nacional de Pesquisas da Amazônia, Manaus, 69067-375 Brazil

**Keywords:** Climate change, ENM, Ecosystem services, Neotropics, Syrphid conservation, Area contraction

## Abstract

Climate change is rapidly reshaping species distributions and threatening ecosystem services, yet continent-wide forecasts for Neotropical hoverflies are virtually absent. Here, we present the first large-scale ecological niche modeling assessment for *Dioprosopa clavata* (Fabricius, 1794), a key syrphid with dual ecological roles in pollination and aphid control. Using 1,214 curated occurrences and an ensemble of MaxEnt, DOMAIN, and GLM, we combined climatic and physiographic predictors (elevation, compound topographic index, profile curvature) to project suitability under SSP2–4.5 (intermediate) and SSP5–8.5 (high-end) scenarios for 2021–2040 and 2081–2100. Models showed excellent predictive performance, with climate explaining ~ 71% of model contribution and physiography ~ 29%, highlighting the buffering role of topographic heterogeneity. Near-term projections retained most of the current range (≈ 88–89% stability), but late-century forecasts revealed dramatic contractions, up to 43.5% habitat loss under SSP5–8.5, particularly in tropical lowlands of northern South America and Central America. These losses were accompanied by increased spatial fragmentation and modest poleward and upslope gains, suggesting climate-driven range shifts toward cooler, higher-elevation refugia. Our findings indicate that *D. clavata* habitats will decline most severely where pollination and pest control services are most needed, agricultural regions of tropical South America, potentially reducing local ecosystem service provision. By identifying priority areas for connectivity and microclimatic refugia, this study offers a mechanical aware forecast and actionable hypotheses to guide field validation, monitoring programs, and climate-smart conservation strategies for an understudied yet functionally crucial pollinator group.

**Supplementary information:**

The online version contains supplementary material available at 10.1007/s00484-026-03147-y.

## Introduction

Hoverflies, or flower flies (Diptera: Syrphidae) are key contributors to ecosystem functioning through complementary services: adults mediate pollination mutualisms, and larval guilds exert top-down control on sternorrhynchous hemipterans in agroecosystems (Pérez-Bañón et al. 2003; Thompson et al. 2010). They also serve as bioindicators of habitat integrity and landscape quality (Sommaggio 1999; Kassebeer 2000; IPCC 2021). Because roughly one-third of crops depend on pollination, syrphids act as important crop pollinators, providing services that are partly overlapping with, but also complementary to, those of bees in crops such as onion, oilseed rape, strawberry and sweet pepper (Inouye et al. 2015; Cook et al. 2020). However, the links between species traits and environment remain insufficiently understood, for example, in terms of species-specific floral niches and dispersal capacity, thereby constraining forecasts of global-change impacts (Inouye et al. 2015; Dunn et al. 2020).

Ecological niche modelling (ENM) plays a central role in predicting species’ geographic ranges and informing biogeography, conservation, and climate-change assessments (Peterson et al. 2011; Peterson and Soberón 2012; Zhu et al. 2013). These modelling approaches intersect with the Wallacean shortfall for many arthropods, where incomplete distributional data limit inference (Mammola et al. 2021). Scenario-based distribution models for syrphids can help anticipate climate-driven range shifts, phenological mismatches, and changes in ecosystem service supply, as well as guide management under Shared Socioeconomic Pathways (SSP) (Inouye et al. 2015; Dunn et al. 2020; IPCC 2021).

In this context, the Neotropics, which hold approximately 30% of global syrphid diversity, are of particular interest. Hoverflies contribute to pollination, biological control and detrital nutrient cycling (Parada-Marin et al. 2025). As ectotherms, their foraging and flight behaviors are tightly regulated by temperature, solar radiation, humidity, cloud cover and wind, and many species remain active under conditions in which bees are inactive (Inouye et al. 2015). Several taxa also perform long-distance seasonal movements that redistribute pollination and pest-control services across regions, indicating that climate and resource phenology can propagate ecological effects far beyond local habitats (Reynolds et al. 2024).

Within this assemblage, *Dioprosopa clavata* (Fabricius, 1794) is a Neotropical syrphine in the tribe Syrphini and the type species of *Dioprosopa* Hull, a New World genus segregated from *Pseudodoros* based on adult morphology and later supported by molecular phylogenies (Hull 1949; Kassebeer 2000; Mengual et al. 2008). *D. clavata* is widely distributed across tropical and subtropical regions of the Americas, occurring in both natural habitats and agroecosystems (Kassebeer 2000). Its larvae are aphidophagous and have also been recorded attacking spittlebugs and other hemipteran pests of economic importance, while adults feed on nectar and pollen, including composites such as *Tridax procumbens*, commonly known as coatbuttons or tridax daisy, thus contributing simultaneously to biological control and pollination networks, including citrus agroecosystems (Rojo et al. 2003; Inouye et al. 2015; Arcaya et al. 2018; Parada-Marin et al. 2025). Recent redescriptions of the third-instar larva and puparium provide diagnostic characters of the cuticle and cephalopharyngeal skeleton that distinguish *D. clavata* from other syrphine genera and reinforce its current systematic placement (Lillo et al. 2021). Given its broad Neotropical range, well-resolved taxonomy and dual role as predator and pollinator, *D. clavata* is an informative model for assessing how climate change may reshape the distribution of aphidophagous hoverflies and the services they provide.

There is increasing evidence confirming that climate change is altering the phenology and geographic distribution of syrphid. In temperate regions, flight periods advance markedly with warming (about 12 days per °C at onset), underscoring the sensitivity of activity and development to temperature regimes (Hassall et al. 2017). Species distribution studies for European syrphids have begun to quantify these patterns. Several analyses for Southeast Europe and the Balkans show projected range contractions in lowland areas and relative persistence or expansion in cooler, wetter mountainous regions, with precipitation seasonality and conditions during the driest months emerging as strong correlates of occurrence (Kaloveloni et al. 2015; Miličić et al. 2018; Milić et al. 2019). Upslope shifts into higher elevation belts may therefore occur simultaneously with declining suitability in hotter or drier lowlands, many of which are agriculturally important, raising the risk of pollinator shortfalls and altered biological control (Kaloveloni et al. 2015; Miličić et al. 2018). Dispersal capacity and dietary breadth appear to be key traits mediating the ability to track suitable climates (Inouye et al. 2015; Reynolds et al. 2024).

Physiographic gradients are likely to further modulate the projected responses of *D. clavata*. Elevation and terrain complexity may influence local water balance and temperature, potentially shaping plant communities and aphid dynamics that underpin adult resources and larval prey. In mountainous areas, cloud immersion and horizontal precipitation can increase moisture inputs, adiabatic cooling may lower temperatures, and epiphyte-rich canopies together with organic soils may improve water retention. These factors, combined with topographic and soil heterogeneity, can generate distinct vegetation assemblages and microclimates along short elevational spans, creating potential moisture-retentive refugia, while valley lowlands are likely to face stronger warming and drought stress (Caglioni et al. 2018). Together with climate change projections, such physiographic gradients may provide a mechanistic basis for anticipating potential gains at higher elevations and probable losses across lowlands for aphidophagous hoverflies (Miličić et al. 2018; Caglioni et al. 2018).

Here, we quantify the present and future potential distribution of *Dioprosopa clavata* across the Americas using an ensemble modelling approach that integrates climatic and physiographic predictors. Specifically, we benchmark the predictive performance of MaxEnt (MXD), Domain (DOM), and Generalized Linear Models (GLM), and we project and compare habitat suitability under SSP2-4.5 and SSP5-8.5 scenarios for the periods 2021–2040 and 2081–2100. We estimate range stability, gain, and loss relative to the current distribution. This design links ecological mechanism to predictive modelling, enabling the identification of potential refugia and vulnerable lowlands to guide monitoring and management.

## Materials and methods

### Occurrence records and data cleaning

**Fig. 1:**
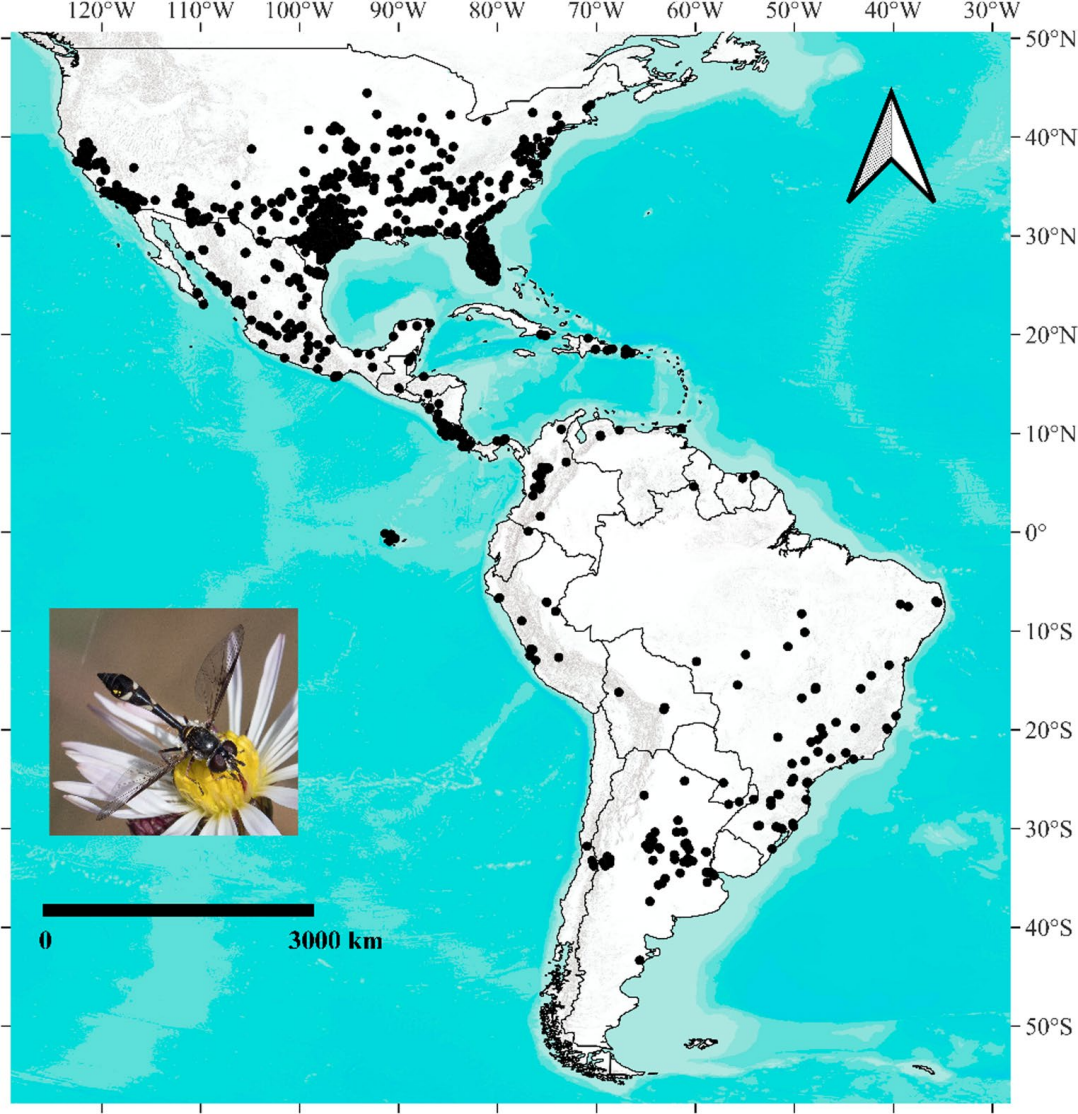
Compilation of known occurrences of *Dioprosopa clavata* (Diptera: Syrphidae) across the Americas. Inset: adult *D. clavata*; photograph by Jim Moore

We assembled distributional data for *Dioprosopa clavata* from two sources: (i) specimens examined in zoological and entomological collections and (ii) vetted records from specialised literature (checklists, catalogues and taxonomic revisions). In addition, we retrieved 8,036 occurrence records from the Global Biodiversity Information Facility (GBIF 2025, 10.15468/dl.3bxkun). All records underwent quality control and validation; after de-duplication and removal of entries that failed basic consistency checks, we remained 1,936 unique and verified occurrences for analysis and the spatial distribution of the 122 unfiltered records is shown (Fig. 1; Supplementary Material S1).

We filtered records to remove duplicates, entries with ambiguous or conflicting coordinates, and points located within urbanized or marine areas. To reduce spatial sampling bias, we applied the *cell-size* occurrence-thinning method (Fourcade et al. 2014), retaining one randomly selected occurrence per grid cell. We set grid cells to twice the resolution of the environmental predictors (2 × 2.5 arc-minutes = 5 arc-minutes; ≈ 9 km at the equator) (Fourcade et al. 2014; Velazco et al. 2019). After filtering and thinning, 1,214 occurrences of *D. clavata* remained.

## Environmental predictors: present and future

### Present (baseline) predictors

We initially considered 22 predictors (Supplementary Material S2). We standardized all raster layers to 2.5 arc-minutes and aligned to a common grid. Nineteen were bioclimatic variables (BIO1–BIO19) describing temperature and precipitation regimes, including annual means, seasonality and extremes (Booth et al. 2014), sourced from WorldClim v2 (Fick and Hijmans 2017). Three physiographic covariates were included: elevation, compound topographic index (CTI; a proxy for potential soil moisture) and profile curvature (pcurv; terrain concavity/convexity affecting flow acceleration), derived from a digital elevation model following Amatulli et al. (2020).

To limit multicollinearity, we computed variance inflation factors (VIF) and iteratively removed predictors with VIF > 10 until all remaining variables met this threshold (Marquardt 1970; Supplementary Material S3). The final set of predictors was then stacked and prepared for modelling.

## Future climate projections

We projected future suitability for *Dioprosopa clavata* using MIROC6 (a CMIP6 general circulation model; Tatebe and Watanabe 2023). We downscaled, bias-corrected montly temperature and precipitation from WorldClim v2 to derive BIO1–BIO19 at 2.5 arc-minutes for two time slices (2021–2040 and 2081–2100) under SSP2–4.5 and SSP5–8.5 (Fick and Hijmans 2017; IPCC 2021). All future rasters were aligned to the current grid, retained the same projection, and were masked to land areas. Following IPCC AR6, we interpret SSP2–4.5 as an intermediate emissions pathway and SSP5–8.5 as a high-end baseline (very high-emissions) scenario (IPCC 2021).

## Ecological niche models

We defined the calibration region for *Dioprosopa clavata* by buffering the occurrence cloud using the maximum inter-point distance, following accessibility (“M”) guidelines species (Barve et al. 2011; Peterson et al. 2011). For GLMs, we generated pseudo-absences at a 1:1 ratio to presences. For MaxEnt, we used randomly selected background points. We distributed both background and pseudo-absence points across the calibration area with an explicit bias towards areas of low predicted suitability (based on BIOCLIM), to reduce overfitting to sampling hotspots (Engler et al. 2004).

We fitted three algorithms representing distinct modelling algorithms: MaxEnt using presences and background points (Phillips et al. 2006; Phillips 2017); DOMAIN using presences only (Hijmans et al. 2017); and GLMs using presences and pseudo-absences (McCullagh and Nelder 1989). To capitalize on complementary strengths (Araujo and New 2007; Thuiller et al. 2019) and ensure adequate predictive skill (Pimenta et al. 2022), we retained models with a True Skill Statistic (TSS) ≥ 0.70 and averaged their continuous predictions into an ensemble.

We applied five-fold cross-validation (Fielding and Bell 1997). We quantified residual spatial autocorrelation using Moran’s I (I = 0.416 ± 0.019). Environmental novelty was assessed using multivariate environmental similarity surfaces (MESS; Elith et al. 2010), which indicated generally positive values (13.099 ± 0.824), i.e., interpolation within the training domain.

Continuous outputs were binarized using the threshold that maximizes the Sørensen similarity, helping to mitigate prevalence bias while balancing true positive and true negative rates (Jiménez-Valverde and Lobo 2007; Leroy et al. 2018). Model performance was summarized with TSS and Sørensen indices (Allouche et al. 2006; Leroy et al. 2018), with values ≥ 0.70 considered satisfactory, based on map inspection and expert knowledge of the species.

We retained only present and future projections that exhibited no high-extrapolation areas, operationalized as having zero pixels with MOP < 0.05 (i.e., no predictions in strongly novel environmental space; Owens et al. 2013). By enforcing this stringent MOP criterion, the projections analyzed are restricted to environmental conditions highly analogous to the calibration domain (> 0.05 MOP), thereby reducing uncertainty associated with extrapolative predictions, as proposed byFitzpatrick and Hargrove (2009) and Elith et al. (2010) (Supplementary Material S4).

We compared binarized ensembles outputs between current conditions and projections for the year 2100 under SSP2–4.5 (intermediate emissions) and SSP5–8.5 (very high emissions) to estimate proportions of range stability, loss and gain. Models were implemented in ENMTML (Andrade et al. 2020) and maps were prepared using QGIS 3.22.14. All analyses were conducted in R version 4.4.3 (R Core Team 2025).

## Results

The consensus (Ensemble) model for *Dioprosopa clavata* showed high predictive performance (TSS = 0.798 ± 0.012; Sørensen = 0.896 ± 0.006). Applying a Sørensen-optimised threshold (cut-off = 0.5679) yielded TPR = 0.879 and TNR = 0.916. For the best-performing single algorithm (Table 1), climatic predictors explained approximately 71.3% of the total contribution, while physiographic predictors (CTI, Pcurv, Elevation) accounted for 28.7% (Supplementary Material S3).

**Table 1:** Algorithms and ensemble model performance for *Dioprosopa clavata*, based on 1,214 presence records. Maximum entropy default (MXD); domain (DOM); generalized linear models (GLM). Values are mean TSS (± SD) and Sørensen (± SD) across model replicates

Species	Algorithm	TSS (± SD)	Sorensen (± SD)
*Dioprosopa clavata*	DOM	0.702 ± 0.016	0.861 ± 0.007
	GLM	0.797 ± 0.013	0.895 ± 0.007
	MXD	0.697 ± 0.043	0.856 ± 0.015
	**Ensemble**	**0.798 ± 0.012**	**0.896 ± 0.006**

## Current and future potential distribution

**Fig. 2:**
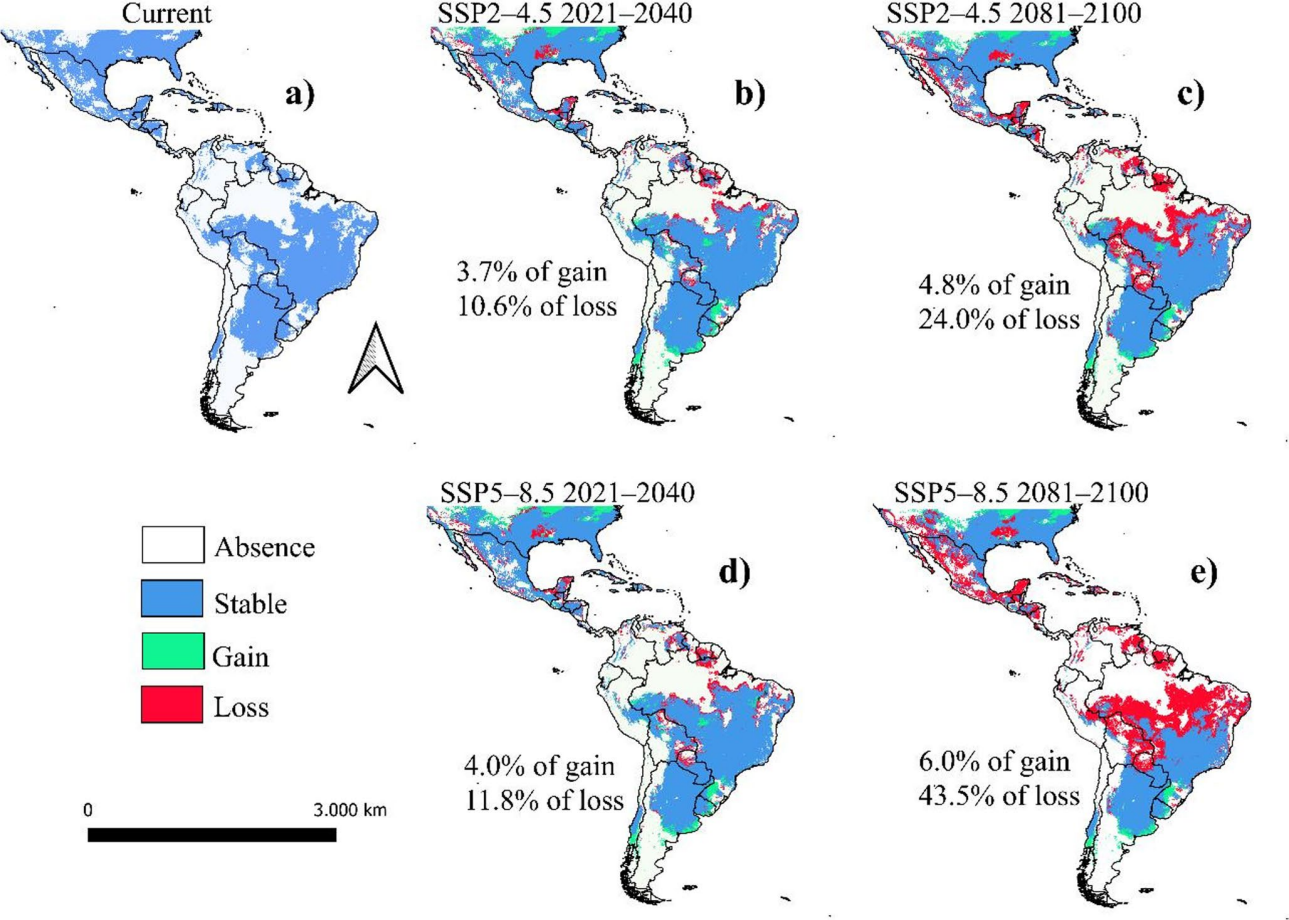
Ensemble projections of the potential climatic suitability of *Dioprosopa clavata* (Diptera: Syrphidae) across the Americas: **a** current climatic conditions; **b**, **c** MIROC6 SSP2–4.5 for 2021–2040 and 2081–2100, respectively; and **d**, **e** MIROC6 SSP5–8.5 for 2021–2040 and 2081–2100, respectively. Cells are classified relative to the present suitable range as absence (white), stable (blue), gain (green) and loss (red)

Current climatic suitability for *Dioprosopa clavata* is widespread across much of North, Central and South America (Fig. 2a). Under SSP2–4.5, near-term projections (2021–2040; Fig. 2b) retain most of the present suitable range (Stable = 89.4 ± 0.6%, Gain = 3.7 ± 0.6%, Loss = 10.6 ± 0.6%; Table 2). By 2081–2100 (Fig. 2c), suitable area declines (Stable = 75.9 ± 0.6%, Gain = 4.9 ± 0.6%, Loss = 24.1 ± 0.6%; Table 2). Under SSP5–8.5, near‐term projections (2021–2040; Fig. 2d) also maintain most of the current range (Stable = 88.2 ± 0.6%, Gain = 4.0 ± 0.6%, Loss = 11.8 ± 0.6%; Table 2), but losses intensify by 2081–2100 (Fig. 2e), with Stable = 56.5 ± 0.6%, Gain = 6.1 ± 0.6% and Loss = 43.5 ± 0.6% (Table 2). Spatially, the scenarios (Figs. 2b–e) show an expanding belt of unsuitability across tropical–equatorial latitudes over time, increasingly fragmenting the distribution and isolating suitable patches toward higher latitudes. Northern South America and Central America are projected to experience the most pronounced contractions by the end of the century.

**Table 2:** Suitable area (ha) and landscape percentages for *Dioprosopa clavata* from the ensemble (SUP) under current and future climates; area = total suitable area per scenario; stable + loss = 100% of current; gain = added vs. current

Model	Scenario	Area (ha)	Stable (%)	Gain (%)	Loss (%)
Ensemble	Current	1.28E + 09	–	–	–
	245/2040	1.19E + 09	89.4 ± 0.6	3.7 ± 0.6	10.6 ± 0.6
	245/2100	1.03E + 09	75.9 ± 0.6	4.9 ± 0.6	24.1 ± 0.6
	585/2040	1.18E + 09	88.2 ± 0.6	4.0 ± 0.6	11.8 ± 0.6
	585/2100	8.00E + 08	56.5 ± 0.6	6.1 ± 0.6	43.5 ± 0.6

## Discussion

This study is, to our knowledge, the first continent-wide ecological niche assessment for the most widely distributed species of *Dioprosopa*. Our models indicate that broad-scale climatic gradients are the primary drivers of the species’ potential range, while physiographic factors such as CTI, plan curvature and elevation add important constraints by modulating local habitat availability. This pattern suggests that macroclimate sets the overall limits of suitability, whereas topographic heterogeneity may shape the configuration and connectivity of suitable areas. In line with expectations for a warm-adapted, widely distributed taxon, projections point to limited changes in total suitable area in the near term under both emission pathways but increasingly pronounced contractions and fragmentation in tropical–equatorial regions toward the end of the century, particularly under SSP5–8.5. These trends highlight the potential vulnerability of populations in northern South America and Central America, in contrast to higher-latitude regions that may act as relative climatic refugia.

The analysis revealed an extreme contraction of suitable habitats in arid and savanna-like biomes, with a projected loss of 43.5% by 2100 under SSP5–8.5, particularly across the Cerrado and Caatinga of South America. Despite this drastic reduction, some arid regions are expected to retain areas of suitability until 2040 under both climate scenarios. The dominance of climatic controls aligns with ENM theory, which operationalises the Grinnellian niche through abiotic surfaces (Thrasher and Grinnell 1917; Soberón 2007; Peterson et al. 2011). However, the substantial contributions of physiographic predictors suggests that water balance and microclimatic conditions modulate climate exposure at landscape scales. In particular, CTI and plan curvature identify zones of flow convergence and moisture retention, which may reduce thermal and hydric stress. In the semi-arid Northeast, these include altitude-driven ‘Brejos de Altitude’, created by orographic uplift and cloud/moisture capture, as well as humid buffers along river basins and riparian corridors. These features may help explain the persistence of suitable patches in otherwise arid landscapes, highlighting physiographic heterogeneity as a stabilising factor against climate-driven habitat loss (Alencar et al. 2022; Dantas et al. 2024).

Our projections for *Dioprosopa clavata* revealed a marked poleward shift in suitable habitats by the end of the century, as equatorial lowlands becoming increasingly unsuitable. This tendency is consistent with large-scale evidence of poleward range shifts in non-migratory European butterflies, where 63% of species expanded northwards by 35–240 km during the twentieth century in response to regional warming (Parmesan et al. 1999). Similar patterns are reported for European syrphids, which tend to gain suitability in cooler mountainous areas while declining in lowlands (Miličić et al. 2018). Likewise, *D. clavata* is projected to lose ground in lowland Cerrado and Caatinga, while gaining potential habitats at higher latitudes. This spatial signal aligns with broader ecological expectations for ectothermic pollinators, whose distributions are tightly constrained by physiological thresholds related to temperature and moisture (Addo-Bediako et al. 2000; Inouye et al. 2015). Taken together, our results reinforce a general biogeographic pattern: warming climates drive contractions at hot equatorial boundaries while enabling expansions into cooler temperate environments.

These shifts have important implications for ecosystem services. Given its dual role as predator and pollinator, the documented predation of *D. clavata* on citrus aphids suggests a potential role in biological control within citrus systems, while adult foraging implies contributions to pollination networks (Sommaggio 1999; Inouye et al. 2015; Arcaya et al. 2018; Cook et al. 2020). Contractions in tropical lowlands along hot equatorial boundaries coincide with major agricultural regions (Goldsmith and Cohn 2017) and may lead to declines in local service provision where demand is highest, even if small gains occur in cooler highlands.

Syrphids remain underrepresented in global assessments of pollinator decline, despite their dual role as pollinators and biological control agents (Arcaya et al. 2018; Cook et al. 2020; Dunn et al. 2020). Our projections show that *D. clavata* is comparably vulnerable to climate-driven range contractions, particularly in tropical regions. This contrasts with the special attention that bees typically receive in global assessments, even though hoverflies can also provide key pollination services. This pattern parallels growing evidence for bees, especially bumblebees (*Bombus*) in temperate regions and native bee assemblages in Brazil, which are consistently identified as highly vulnerable to climate change (Giannini et al. 2012, 2017; Kerr et al. 2015; Soroye et al. 2020). Studies indicate that climate change, deforestation and the loss of native vegetation will substantially reduce the richness, distribution and pollination services provided by these bee groups, with cascading effects on plant reproduction and crop productivity (Elias et al. 2017; Giannini et al. 2017; Frigero et al. 2025). These findings emphasize that pollination services are threatened not only by the decline of bee populations, but also by the decline of functionally important but understudied pollinators such as hoverflies.

Ecological niche models based solely on abiotic variables do not capture biotic interactions or dispersal constraints, which can reduce transferability under novel climates (Soberón 2007; Peterson and Soberón 2012). Future efforts should incorporate key floral resources and host plants to refine estimates for syrphids. In addition, major knowledge gaps persist for arthropods, especially in the Neotropics, where occurrence data remain sparse and uneven (Hortal et al. 2015; Mammola et al. 2021). While gridded climate layers are essential for broad-scale inference, they can obscure fine-scale environmental heterogeneity relevant to hoverflies and their sternorrhynchous hemipterans prey. Addressing these limitations will require expanded field sampling, digitization and curation of museum specimens (Balmford and Gaston 1999), and integration of movement ecology and trait-based filters considering dispersal capacity, floral niche breadth, and thermal tolerance. Despite these caveats, the ensemble framework implemented here showed strong predictive performance and adheres to current best practices in ENM. Similar modelling pipelines developed by our group have already been applied to other insect taxa to support conservation planning and climate-change assessments (Aguiar et al. 2023; Cruz et al. 2023; Alencar et al. 2024a, b, 2025a, b). These strengths and limitations together highlight both the promise and the constraints of predictive models in understanding pollinator responses to environmental change.

From a management perspective, two key insights emerge from our findings. (i) The pronounced vulnerability of *D. clavata* in tropical lowlands, where dependence on pollination and biological control is high, indicates that conservation actions should prioritise microclimatic refugia and physiographic heterogeneity, for example by protecting biological corridors, agroforestry mosaics and other landscape elements that buffer heat and drought, helping sustain syrphid-mediated services where climate-driven contractions are expected to be most severe. (ii) The projected poleward and elevational shifts highlight the need to maintain connectivity across latitudinal and altitudinal gradients. For highly mobile syrphids, including migratory species, permeable landscapes and functional corridors of sequentially flowering habitats can facilitate climate tracking while sustaining long-distance pollen transfer and gene flow (Doyle et al. 2020; Schleimer and Frantz 2025). Empirical evidence from forest-dominated agricultural mosaics shows that hoverfly richness and abundance in mass-flowering crops increase with surrounding forest and other non-arable habitats, whereas arable-dominated landscapes support poorer communities (Toikkanen et al. 2022), reinforcing the importance of conserving forest patches, riparian strips, hedgerows and transitional woodland along climatic gradients. Together, these insights align with climate-smart conservation frameworks and underscore that neglected pollinator groups such as hoverflies must be explicitly integrated into global biodiversity and agroecosystem strategies to anticipate and mitigate cascading impacts of climate and land-use change.

Besides hoverflies, several other dipteran families, such as Anthomyiidae, Ceratopogonidae and Tachinidae, also act as pollinators (Skevington and Dang 2002; Courtney et al. 2017). Although bees and syrphids are often regarded as the main pollinators, there is a rich diversity of flies that fulfil this ecological role worldwide. Consequently, further ENM studies focusing on pollinating flies are needed to broaden our understanding of how climate change may affect pollinating insects in general, rather than restricting attention solely to bees.

## Supplementary information

Below is the link to the electronic supplementary material.

Supplementary Material 1

## Data Availability

Data generated or analysed during this study are provided in full within the published article and its supplementary materials.
