# Supplementary material for: Small gains, large losses: range shifts of the hoverfly Dioprosopa clavata (Fabricius, 1794) (Diptera: Syrphidae) to 2100
Source: Int J Biometeorol. 2026 Feb 24;70(3):71. doi: 10.1007/s00484-026-03147-y (PMC12932299; doi:10.1007/s00484-026-03147-y)
Supplement: Supplementary file 1 — Supplementary Material 1 [file 484_2026_3147_MOESM1_ESM.pdf]

Supplementary File

Small gains, large losses: Range shifts of the hoverfly *Dioprosopa clavata* (Fabricius, 1794) (Diptera: Syrphidae) to 2100

Journal: *International Journal of Biometeorology*

Janderson Batista Rodrigues Alencar[1*], João Paulo Nunes[2], Matheus Augusto do Nascimento[2], Alessandre Pereira-Colavite[2], Adeilson de Melo Silva[3], Clarissa Rosa[1,4]

[1]Programa Institucional de Pós-Doutorado, Pós-graduação em Ecologia, Instituto Nacional de Pesquisas da Amazônia, Constelação Cruzeiro do Sul Street, Aleixo, ZIP 69060-062 Manaus- AM, Brazil. ORCID – 0000-0001-9482-7866
[2]Laboratório de Entomologia, Departamento de Sistemática e Ecologia, CCEN, Universidade Federal da Paraíba. JPN ORCID – 0000-0002-0441-4935; MAN ORCID –0000-0001-5259-9738; APC ORCID –0000-0002-7660-8384
[3]Núcleo de Ecologia e Monitoramento Ambiental (NEMA), Universidade Federal do Vale do São Francisco, Rodovia BR-407, KM 12 Lote 543 - S/N C1, ZIP 56300-000 Petrolina-PE, Brazil. ORCID – 0000-0001-5238-9968
[4]Coordenação de Dinâmica Ambiental (CODAM), Instituto Nacional de Pesquisas da Amazônia, Manaus 69067-375, Brasil; ORCID – 0000-0001-7462-1991
*Corresponding author: jandersonrn@gmail.com

Supplementary File S1. Raw georeferenced occurrence records of *Dioprosopa clavata* used to map the unfiltered points shown in Fig. 1, including locality information and original data sources

species	country	state_province	locality	latitude_decimal	longitude_decimal	source
Dioprosopa clavatus	United States of America	Texas	Travis County	30.366064	-98.127259	Occurrence Download https://doi.org/10.15468/dl.3bxkun
Dioprosopa clavatus	Mexico	Querétaro	Corregidora	20.522076	-100.388049	Occurrence Download https://doi.org/10.15468/dl.3bxkun
Dioprosopa clavatus	United States of America	Texas	Dripping Springs	30.204251	-98.08016	Occurrence Download https://doi.org/10.15468/dl.3bxkun
Dioprosopa clavatus	Mexico	Sinaloa	Las Higueras	25.036376	-107.544473	Occurrence Download https://doi.org/10.15468/dl.3bxkun
Dioprosopa clavatus	Belize	Cayo	Spanish Lookout	17.282217	-88.998175	Occurrence Download https://doi.org/10.15468/dl.3bxkun
Dioprosopa clavatus	Brazil	Minas Gerais	Sacramento	-19.821606	-47.32694	Occurrence Download https://doi.org/10.15468/dl.3bxkun
Dioprosopa clavatus	United States of America	Texas	Georgetown	30.682927	-97.713306	Occurrence Download https://doi.org/10.15468/dl.3bxkun
Dioprosopa clavatus	United States of America	Florida	Saint Petersburg	27.653584	-82.677545	Occurrence Download https://doi.org/10.15468/dl.3bxkun
Dioprosopa clavatus	Mexico	Oaxaca	Santa María Huatulco	15.854842	-96.360933	Occurrence Download https://doi.org/10.15468/dl.3bxkun
Dioprosopa clavatus	United States of America	Texas	San Marcos	29.910067	-97.894095	Occurrence Download https://doi.org/10.15468/dl.3bxkun
Dioprosopa clavatus	Argentina	Córdoba	Cuesta Blanca	-31.47877	-64.585881	Occurrence Download https://doi.org/10.15468/dl.3bxkun
Dioprosopa clavatus	Brazil	Distrito Federal	Lago Sul	-15.796278	-47.809968	Occurrence Download https://doi.org/10.15468/dl.3bxkun
Dioprosopa clavatus	Chile	Región Metropolitana de Santiago	Lo Barnechea	-33.229046	-70.455648	Occurrence Download https://doi.org/10.15468/dl.3bxkun
Dioprosopa clavatus	Argentina	Santa Fe	Municipio de San Cristóbal	-30.313682	-61.232484	Occurrence Download https://doi.org/10.15468/dl.3bxkun
Dioprosopa clavatus	Mexico	Sinaloa	Culiacán	24.84111	-107.354625	Occurrence Download https://doi.org/10.15468/dl.3bxkun
Dioprosopa clavatus	Argentina	Córdoba	Oliva	-32.041482	-63.571854	Occurrence Download https://doi.org/10.15468/dl.3bxkun
Dioprosopa clavatus	United States of America	Florida	Minneola	28.625997	-81.700181	Occurrence Download https://doi.org/10.15468/dl.3bxkun
Dioprosopa clavatus	United States of America	California	Indian Wells	33.670883	-116.365617	Occurrence Download https://doi.org/10.15468/dl.3bxkun
Dioprosopa clavatus	United States of America	California	Indian Wells	33.670892	-116.365608	Occurrence Download https://doi.org/10.15468/dl.3bxkun
Dioprosopa clavatus	United States of America	California	Indian Wells	33.670888	-116.365595	Occurrence Download https://doi.org/10.15468/dl.3bxkun
Dioprosopa clavatus	United States of America	California	Indian Wells	33.670831	-116.365525	Occurrence Download https://doi.org/10.15468/dl.3bxkun
Dioprosoma clavatus	United States of America	California	Indian Wells	33.670825	-116.365647	Occurrence Download https://doi.org/10.15468/dl.3bxkun
Dioprosopa clavatus	United States of America	California	Indian Wells	33.670833	-116.365617	Occurrence Download https://doi.org/10.15468/dl.3bxkun

Dioprosopa clavatus	United States of America	California	Indian Wells	33.670855	-116.36557	Occurrence Download https://doi.org/10.15468/dl.3bxkun
Dioprosopa clavatus	United States of America	California	Indian Wells	33.702321	-116.363471	Occurrence Download https://doi.org/10.15468/dl.3bxkun
Dioprosopa clavatus	Colombia	Antioquia	Sabaneta	6.145493	-75.621786	Occurrence Download https://doi.org/10.15468/dl.3bxkun
Dioprosopa clavatus	United States of America	California	Palm Desert	33.685936	-116.375512	Occurrence Download https://doi.org/10.15468/dl.3bxkun
Dioprosopa clavatus	United States of America	California	Indian Wells	33.670856	-116.365592	Occurrence Download https://doi.org/10.15468/dl.3bxkun
Dioprosopa clavatus	United States of America	California	New Santa Rosa / Sew'ia	33.63499	-116.409649	Occurrence Download https://doi.org/10.15468/dl.3bxkun
Dioprosopa clavatus	United States of America	California	Indian Wells	33.670892	-116.365592	Occurrence Download https://doi.org/10.15468/dl.3bxkun
Dioprosopa clavatus	United States of America	California	Riverside County	33.667915	-116.407821	Occurrence Download https://doi.org/10.15468/dl.3bxkun
Dioprosopa clavatus	United States of America	Florida	Brevard County	28.657379	-80.754402	Occurrence Download https://doi.org/10.15468/dl.3bxkun
Dioprosopa clavatus	Mexico	Veracruz	Xalapa	19.518537	-96.944475	Occurrence Download https://doi.org/10.15468/dl.3bxkun
Dioprosopa clavatus	United States of America	California	Indian Wells	33.670863	-116.365578	Occurrence Download https://doi.org/10.15468/dl.3bxkun
Dioprosopa clavatus	United States of America	California	San Diego County	33.350023	-117.334107	Occurrence Download https://doi.org/10.15468/dl.3bxkun
Dioprosopa clavatus	United States of America	Florida	Alachua County	29.730321	-82.332059	Occurrence Download https://doi.org/10.15468/dl.3bxkun
Dioprosopa clavatus	United States of America	Florida	Seminole County	28.705451	-81.123618	Occurrence Download https://doi.org/10.15468/dl.3bxkun
Dioprosopa clavatus	Argentina	Santa Fe	Municipio de Desvío Arijón	-31.831971	-60.875669	Occurrence Download https://doi.org/10.15468/dl.3bxkun
Dioprosopa clavatus	Brazil	Santa Catarina	Itajaí	-27.050426	-48.806981	Occurrence Download https://doi.org/10.15468/dl.3bxkun
Dioprosopa clavatus	United States of America	California	Palm Springs	33.77871	-116.555573	Occurrence Download https://doi.org/10.15468/dl.3bxkun
Dioprosopa clavatus	United States of America	Texas	Travis County	30.250927	-97.932244	Occurrence Download https://doi.org/10.15468/dl.3bxkun
Dioprosopa clavatus	Mexico	Sinaloa	Culiacán	24.759077	-107.466832	Occurrence Download https://doi.org/10.15468/dl.3bxkun
Dioprosopa clavatus	United States of America	Texas	Hays County	30.043313	-97.813477	Occurrence Download https://doi.org/10.15468/dl.3bxkun
Dioprosopa clavatus	United States of America	Texas	Flower Mound	33.023887	-97.145724	Occurrence Download https://doi.org/10.15468/dl.3bxkun
Dioprosopa clavatus	United States of America	Texas	Hidalgo County	26.365079	-98.339242	Occurrence Download https://doi.org/10.15468/dl.3bxkun
Dioprosopa clavatus	United States of America	Florida	Port Saint Lucie	27.291699	-80.276027	Occurrence Download https://doi.org/10.15468/dl.3bxkun
Dioprosopa clavatus	United States of America	Louisiana	St. George	30.35669	-91.095201	Occurrence Download https://doi.org/10.15468/dl.3bxkun
Dioprosopa clavatus	Argentina	Córdoba	Los Chañares	-31.714286	-64.443257	Occurrence Download https://doi.org/10.15468/dl.3bxkun
Dioprosopa clavatus	United States of America	Texas	San Antonio	29.251895	-98.555987	Occurrence Download https://doi.org/10.15468/dl.3bxkun
Dioprosopa clavatus	United States of America	Texas	San Antonio	29.639929	-98.629723	Occurrence Download https://doi.org/10.15468/dl.3bxkun
Dioprosopa clavatus	United States of America	Florida	Broward County	26.23563	-80.462647	Occurrence Download https://doi.org/10.15468/dl.3bxkun
Dioprosopa clavatus	United States of America	Texas	San Antonio	29.251283	-98.546703	Occurrence Download https://doi.org/10.15468/dl.3bxkun
Dioprosopa clavatus	United States of America	Texas	Stockdale	29.236907	-97.960003	Occurrence Download https://doi.org/10.15468/dl.3bxkun
Dioprosopa clavatus	United States of America	Texas	San Antonio	29.4698	-98.362042	Occurrence Download https://doi.org/10.15468/dl.3bxkun
Dioprosopa clavatus	United States of America	Florida	Pasco County	28.202773	-82.523579	Occurrence Download https://doi.org/10.15468/dl.3bxkun
Dioprosopa clavatus	United States of America	Florida	Palm Beach County	26.496528	-80.215563	Occurrence Download https://doi.org/10.15468/dl.3bxkun
Dioprosopa clavatus	United States of America	Texas	Blanco	30.092219	-98.422389	Occurrence Download https://doi.org/10.15468/dl.3bxkun
Dioprosopa clavatus	United States of America	Texas	Lee County	30.305246	-96.822637	Occurrence Download https://doi.org/10.15468/dl.3bxkun
Dioprosopa clavatus	United States of America	Florida	Salt Springs	29.357863	-81.733564	Occurrence Download https://doi.org/10.15468/dl.3bxkun
Dioprosopa clavatus	United States of America	Texas	Kendall County	29.880863	-98.614303	Occurrence Download https://doi.org/10.15468/dl.3bxkun
Dioprosopa clavatus	United States of America	Texas	San Saba County	31.270307	-98.624321	Occurrence Download https://doi.org/10.15468/dl.3bxkun
Dioprosopa clavatus	United States of America	Arizona	Pima County	32.38753	-110.901705	Occurrence Download https://doi.org/10.15468/dl.3bxkun

Dioprosopa clavatus	United States of America	Texas	Temple	31.060936	-97.380462	Occurrence Download https://doi.org/10.15468/dl.3bxkun
Dioprosopa clavatus	United States of America	Texas	Providence	30.656787	-94.910098	Occurrence Download https://doi.org/10.15468/dl.3bxkun
Dioprosopa clavatus	United States of America	Florida	Gainesville	29.786255	-82.377306	Occurrence Download https://doi.org/10.15468/dl.3bxkun
Dioprosopa clavatus	United States of America	Texas	Parker County	32.9935	-97.580117	Occurrence Download https://doi.org/10.15468/dl.3bxkun
Dioprosopa clavatus	United States of America	Texas	Rambo Estates Colonia	26.311707	-98.087175	Occurrence Download https://doi.org/10.15468/dl.3bxkun
Dioprosopa clavatus	Mexico	Nuevo León	Juárez	25.638687	-100.165039	Occurrence Download https://doi.org/10.15468/dl.3bxkun
Dioprosopa clavatus	United States of America	Texas	San Saba County	31.271241	-98.619486	Occurrence Download https://doi.org/10.15468/dl.3bxkun
Dioprosopa clavatus	United States of America	Texas	Blanco	30.103799	-98.42598	Occurrence Download https://doi.org/10.15468/dl.3bxkun
Dioprosopa clavatus	Mexico	Nuevo León	Juárez	25.638697	-100.16503	Occurrence Download https://doi.org/10.15468/dl.3bxkun
Dioprosopa clavatus	United States of America	Texas	Bryan	30.676734	-96.417407	Occurrence Download https://doi.org/10.15468/dl.3bxkun
Dioprosopa clavatus	United States of America	Texas	Hays County	30.043142	-97.813428	Occurrence Download https://doi.org/10.15468/dl.3bxkun
Dioprosopa clavatus	United States of America	Texas	Comal County	29.973796	-98.281279	Occurrence Download https://doi.org/10.15468/dl.3bxkun
Dioprosopa clavatus	United States of America	Texas	Parker County	32.993622	-97.580117	Occurrence Download https://doi.org/10.15468/dl.3bxkun
Dioprosopa clavatus	United States of America	Texas	Huntsville	30.660729	-95.533999	Occurrence Download https://doi.org/10.15468/dl.3bxkun
Dioprosopa clavatus	United States of America	Florida	Honore	27.23666	-82.261236	Occurrence Download https://doi.org/10.15468/dl.3bxkun
Dioprosopa clavatus	United States of America	Texas	Alvin	29.439932	-95.225008	Occurrence Download https://doi.org/10.15468/dl.3bxkun
Dioprosopa clavatus	United States of America	Texas	Andrews	32.324188	-102.557022	Occurrence Download https://doi.org/10.15468/dl.3bxkun
Dioprosopa clavatus	United States of America	Florida	Cutler Bay	25.559167	-80.347012	Occurrence Download https://doi.org/10.15468/dl.3bxkun
Dioprosopa clavatus	United States of America	Texas	Georgetown	30.703361	-97.770656	Occurrence Download https://doi.org/10.15468/dl.3bxkun
Dioprosopa clavatus	United States of America	Texas	Abilene	32.59748	-99.689361	Occurrence Download https://doi.org/10.15468/dl.3bxkun
Dioprosopa clavatus	United States of America	Texas	Parker County	32.993645	-97.580192	Occurrence Download https://doi.org/10.15468/dl.3bxkun
Dioprosopa clavatus	United States of America	Texas	Parker County	32.993605	-97.58013	Occurrence Download https://doi.org/10.15468/dl.3bxkun
Dioprosopa clavatus	United States of America	Texas	Austin	30.185489	-97.873265	Occurrence Download https://doi.org/10.15468/dl.3bxkun
Dioprosopa clavatus	Mexico	Sinaloa	La Brecha	25.371109	-108.424416	Occurrence Download https://doi.org/10.15468/dl.3bxkun
Dioprosopa clavatus	United States of America	Florida	Pasco County	28.424963	-82.49453	Occurrence Download https://doi.org/10.15468/dl.3bxkun
Dioprosopa clavatus	United States of America	Florida	Martin County	27.03053	-80.376268	Occurrence Download https://doi.org/10.15468/dl.3bxkun
Dioprosopa clavatus	United States of America	Arizona	Yavapai County	34.667481	-111.71539	Occurrence Download https://doi.org/10.15468/dl.3bxkun
Dioprosopa clavatus	United States of America	Texas	Parker County	32.993603	-97.5801	Occurrence Download https://doi.org/10.15468/dl.3bxkun
Dioprosopa clavatus	United States of America	Texas	Round Rock	30.548789	-97.625575	Occurrence Download https://doi.org/10.15468/dl.3bxkun
Dioprosopa clavatus	United States of America	Texas	Tomball	30.026826	-95.629286	Occurrence Download https://doi.org/10.15468/dl.3bxkun
Dioprosopa clavatus	United States of America	Texas	Thompsons	29.525842	-95.639953	Occurrence Download https://doi.org/10.15468/dl.3bxkun
Dioprosopa clavatus	Mexico	Michoacán	Morelia	19.673908	-101.213633	Occurrence Download https://doi.org/10.15468/dl.3bxkun
Dioprosopa clavatus	United States of America	Texas	Bexar County	29.442291	-98.260469	Occurrence Download https://doi.org/10.15468/dl.3bxkun
Dioprosopa clavatus	United States of America	Alabama	Elberta	30.360468	-87.575147	Occurrence Download https://doi.org/10.15468/dl.3bxkun
Dioprosopa clavatus	United States of America	Texas	Temple	31.060939	-97.380488	Occurrence Download https://doi.org/10.15468/dl.3bxkun
Dioprosopa clavatus	United States of America	Arkansas	Rogers	36.330537	-94.072623	Occurrence Download https://doi.org/10.15468/dl.3bxkun
Dioprosopa clavatus	United States of America	Florida	Tampa	27.849863	-82.562724	Occurrence Download https://doi.org/10.15468/dl.3bxkun
Dioprosopa clavatus	United States of America	Florida	Jacksonville	30.144144	-81.655284	Occurrence Download https://doi.org/10.15468/dl.3bxkun
Dioprosopa clavatus	United States of America	Florida	Hernando County	28.590191	-82.495077	Occurrence Download https://doi.org/10.15468/dl.3bxkun

Dioprosopa clavatus	United States of America	Texas	Polk County	30.836124	-94.848123	Occurrence Download https://doi.org/10.15468/dl.3bxkun
Dioprosopa clavatus	United States of America	Texas	Morgan's Point Resort	31.160473	-97.442205	Occurrence Download https://doi.org/10.15468/dl.3bxkun
Dioprosopa clavatus	United States of America	Texas	Lubbock	33.531478	-101.826804	Occurrence Download https://doi.org/10.15468/dl.3bxkun
Dioprosopa clavatus	United States of America	California	Los Angeles	33.717555	-118.316232	Occurrence Download https://doi.org/10.15468/dl.3bxkun
Dioprosopa clavatus	United States of America	Texas	Collin County	33.136404	-96.56158	Occurrence Download https://doi.org/10.15468/dl.3bxkun
Dioprosopa clavatus	Mexico	Sinaloa	Culiacán	24.835048	-107.446283	Occurrence Download https://doi.org/10.15468/dl.3bxkun
Dioprosopa clavatus	United States of America	Texas	Hill Country Retreat	29.47988	-98.75098	Occurrence Download https://doi.org/10.15468/dl.3bxkun
Dioprosopa clavatus	United States of America	Florida	Manatee County	27.481894	-82.344138	Occurrence Download https://doi.org/10.15468/dl.3bxkun
Dioprosopa clavatus	United States of America	Texas	Blanco	30.103787	-98.425982	Occurrence Download https://doi.org/10.15468/dl.3bxkun
Dioprosopa clavatus	United States of America	Texas	Fort Worth	32.993171	-97.362199	Occurrence Download https://doi.org/10.15468/dl.3bxkun
Dioprosopa clavatus	United States of America	Texas	Blanco	30.103789	-98.425981	Occurrence Download https://doi.org/10.15468/dl.3bxkun
Dioprosopa clavatus	United States of America	Oklahoma	Broken Arrow	36.043867	-95.842248	Occurrence Download https://doi.org/10.15468/dl.3bxkun
Dioprosopa clavatus	United States of America	New Mexico	Otero County	33.046218	-106.156047	Occurrence Download https://doi.org/10.15468/dl.3bxkun
Dioprosopa clavatus	United States of America	Texas	Temple	31.126245	-97.464975	Occurrence Download https://doi.org/10.15468/dl.3bxkun
Dioprosopa clavatus	United States of America	Texas	Collin County	33.279275	-96.625983	Occurrence Download https://doi.org/10.15468/dl.3bxkun
Dioprosopa clavatus	United States of America	Oklahoma	Norman	35.194349	-97.46145	Occurrence Download https://doi.org/10.15468/dl.3bxkun
Dioprosopa clavatus	United States of America	Texas	Cinco Ranch	29.720395	-95.807787	Occurrence Download https://doi.org/10.15468/dl.3bxkun
Dioprosopa clavatus	United States of America	Mississippi	Oktibbeha County	33.451117	-88.796014	Occurrence Download https://doi.org/10.15468/dl.3bxkun
Dioprosopa clavatus	United States of America	Texas	Prosper	33.237337	-96.867317	Occurrence Download https://doi.org/10.15468/dl.3bxkun
Dioprosopa clavatus	United States of America	Florida	Lake Wales	27.93837	-81.576425	Occurrence Download https://doi.org/10.15468/dl.3bxkun
Dioprosopa clavatus	United States of America	Virginia	Yorktown	37.213875	-76.513604	Occurrence Download https://doi.org/10.15468/dl.3bxkun
Dioprosopa clavatus	United States of America	Texas	Rockwall	32.965424	-96.437504	Occurrence Download https://doi.org/10.15468/dl.3bxkun
Dioprosopa clavatus	United States of America	Texas	Little Elm	33.188294	-96.89054	Occurrence Download https://doi.org/10.15468/dl.3bxkun
Dioprosopa clavatus	United States of America	Florida	Gulf City	27.690942	-82.5018	Occurrence Download https://doi.org/10.15468/dl.3bxkun
Dioprosopa clavatus	United States of America	Texas	Hood County	32.487439	-97.706786	Occurrence Download https://doi.org/10.15468/dl.3bxkun
Dioprosopa clavatus	United States of America	Texas	Fulshear	29.710432	-95.877588	Occurrence Download https://doi.org/10.15468/dl.3bxkun
Dioprosopa clavatus	United States of America	Florida	Pasco County	28.424787	-82.494628	Occurrence Download https://doi.org/10.15468/dl.3bxkun
Dioprosopa clavatus	United States of America	Florida	Lely	26.112792	-81.731429	Occurrence Download https://doi.org/10.15468/dl.3bxkun
Dioprosopa clavatus	United States of America	Texas	Little Elm	33.225302	-96.92269	Occurrence Download https://doi.org/10.15468/dl.3bxkun
Dioprosopa clavatus	United States of America	Arizona	Cochise County	31.883499	-109.205948	Occurrence Download https://doi.org/10.15468/dl.3bxkun
Dioprosopa clavatus	Mexico	Chihuahua	Colonia Francisco Portillo (Los Jáquez)	28.261899	-105.474705	Occurrence Download https://doi.org/10.15468/dl.3bxkun
Dioprosopa clavatus	United States of America	Texas	Sugar Land	29.549326	-95.597612	Occurrence Download https://doi.org/10.15468/dl.3bxkun
Dioprosopa clavatus	United States of America	Texas	Reina del Sol Mobile Home Estates Colonia	26.235001	-98.068033	Occurrence Download https://doi.org/10.15468/dl.3bxkun
Dioprosopa clavatus	United States of America	Texas	Boerne	29.782153	-98.70928	Occurrence Download https://doi.org/10.15468/dl.3bxkun
Dioprosopa clavatus	United States of America	Oklahoma	Norman	35.257217	-97.44767	Occurrence Download https://doi.org/10.15468/dl.3bxkun
Dioprosopa clavatus	United States of America	Arizona	Tucson	32.243283	-111.167862	Occurrence Download https://doi.org/10.15468/dl.3bxkun
Dioprosopa clavatus	United States of America	Florida	Martin County	27.137339	-80.23882	Occurrence Download https://doi.org/10.15468/dl.3bxkun
Dioprosopa clavatus	United States of America	Texas	San Antonio	29.333035	-98.454907	Occurrence Download https://doi.org/10.15468/dl.3bxkun
Dioprosopa clavatus	United States of America	Alabama	Fairhope	30.511142	-87.915499	Occurrence Download https://doi.org/10.15468/dl.3bxkun

Dioprosopa clavatus	United States of America	Texas	Kerr County	29.998218	-99.26427	Occurrence Download https://doi.org/10.15468/dl.3bxkun
Dioprosopa clavatus	United States of America	Texas	Johnson County	32.418953	-97.551155	Occurrence Download https://doi.org/10.15468/dl.3bxkun
Dioprosopa clavatus	United States of America	Texas	Fort Bend County	29.584135	-95.446075	Occurrence Download https://doi.org/10.15468/dl.3bxkun
Dioprosopa clavatus	United States of America	Texas	Brazoria County	29.527325	-95.307276	Occurrence Download https://doi.org/10.15468/dl.3bxkun
Dioprosopa clavatus	United States of America	Alabama	Fairhope	30.534624	-87.903183	Occurrence Download https://doi.org/10.15468/dl.3bxkun
Dioprosopa clavatus	United States of America	Texas	Cameron County	26.161613	-97.794207	Occurrence Download https://doi.org/10.15468/dl.3bxkun
Dioprosopa clavatus	United States of America	Texas	Argyle	33.168509	-97.17382	Occurrence Download https://doi.org/10.15468/dl.3bxkun
Dioprosopa clavatus	United States of America	Florida	Polk County	28.106962	-82.05481	Occurrence Download https://doi.org/10.15468/dl.3bxkun
Dioprosopa clavatus	United States of America	Texas	Tarrant County	32.799307	-97.531092	Occurrence Download https://doi.org/10.15468/dl.3bxkun
Dioprosopa clavatus	United States of America	Florida	Brevard County	28.697329	-80.855422	Occurrence Download https://doi.org/10.15468/dl.3bxkun
Dioprosopa clavatus	United States of America	Oklahoma	Norman	35.194475	-97.461487	Occurrence Download https://doi.org/10.15468/dl.3bxkun
Dioprosopa clavatus	United States of America	Texas	Wharton County	29.479541	-96.076732	Occurrence Download https://doi.org/10.15468/dl.3bxkun
Dioprosopa clavatus	United States of America	Texas	Weatherford	32.736078	-97.754732	Occurrence Download https://doi.org/10.15468/dl.3bxkun
Dioprosopa clavatus	United States of America	Florida	Brooker	29.890817	-82.331253	Occurrence Download https://doi.org/10.15468/dl.3bxkun
Dioprosopa clavatus	United States of America	Georgia	Skidaway Island	31.95614	-81.024646	Occurrence Download https://doi.org/10.15468/dl.3bxkun
Dioprosopa clavatus	United States of America	Pennsylvania	Liberty Township	39.773051	-77.335491	Occurrence Download https://doi.org/10.15468/dl.3bxkun
Dioprosopa clavatus	United States of America	Mississippi	Hernando	34.817543	-89.983638	Occurrence Download https://doi.org/10.15468/dl.3bxkun
Dioprosopa clavatus	United States of America	Texas	Fort Worth	32.750074	-97.571358	Occurrence Download https://doi.org/10.15468/dl.3bxkun
Dioprosopa clavatus	United States of America	Texas	Kerr County	29.9984	-99.264195	Occurrence Download https://doi.org/10.15468/dl.3bxkun
Dioprosopa clavatus	United States of America	Georgia	Skidaway Island	31.956227	-81.024673	Occurrence Download https://doi.org/10.15468/dl.3bxkun
Dioprosopa clavatus	United States of America	Kansas	Douglas County	38.840473	-95.217845	Occurrence Download https://doi.org/10.15468/dl.3bxkun
Dioprosopa clavatus	United States of America	Texas	Comal County	29.821363	-98.382845	Occurrence Download https://doi.org/10.15468/dl.3bxkun
Dioprosopa clavatus	United States of America	Florida	Inglis	29.021315	-82.637446	Occurrence Download https://doi.org/10.15468/dl.3bxkun
Dioprosopa clavatus	Argentina	Córdoba	Mallín	-31.299678	-64.57228	Occurrence Download https://doi.org/10.15468/dl.3bxkun
Dioprosopa clavatus		Texas	Aransas County	28.130156	-96.985324	Occurrence Download https://doi.org/10.15468/dl.3bxkun
Dioprosopa clavatus	Brazil	Minas Gerais	Itamonte	-22.329778	-44.808865	Occurrence Download https://doi.org/10.15468/dl.3bxkun
Dioprosopa clavatus	United States of America	Texas	Palmview South	26.217523	-98.37592	Occurrence Download https://doi.org/10.15468/dl.3bxkun
Dioprosopa clavatus	Mexico	Sinaloa	Laguna Colorada	24.671477	-107.293931	Occurrence Download https://doi.org/10.15468/dl.3bxkun
Dioprosopa clavatus	United States of America	Texas	Palmview South	26.217918	-98.376232	Occurrence Download https://doi.org/10.15468/dl.3bxkun
Dioprosopa clavatus	Argentina	Santa Fe	San Jerónimo Norte	-31.550832	-61.06775	Occurrence Download https://doi.org/10.15468/dl.3bxkun
Dioprosopa clavatus	Argentina	Santa Fe		-31.550912	-61.067689	Occurrence Download https://doi.org/10.15468/dl.3bxkun
Dioprosopa clavatus	Argentina	Santa Fe	San Jerónimo Norte	-31.550978	-61.067707	Occurrence Download https://doi.org/10.15468/dl.3bxkun
Dioprosopa clavatus	Mexico	Querétaro	Corregidora	20.521976	-100.388231	Occurrence Download https://doi.org/10.15468/dl.3bxkun
Dioprosopa clavatus	Argentina	Córdoba	Cuesta Blanca	-31.479271	-64.586225	Occurrence Download https://doi.org/10.15468/dl.3bxkun
Dioprosopa clavatus	Mexico	Tamaulipas	Mission	26.179914	-98.384439	Occurrence Download https://doi.org/10.15468/dl.3bxkun
Dioprosopa clavatus	United States of America	Texas	Progreso	26.090052	-97.98074	Occurrence Download https://doi.org/10.15468/dl.3bxkun
Dioprosopa clavatus	United States of America	Texas	Palmview South	26.217301	-98.375293	Occurrence Download https://doi.org/10.15468/dl.3bxkun
Dioprosopa clavatus	United States of America	Texas	Progreso	26.087472	-97.979812	Occurrence Download https://doi.org/10.15468/dl.3bxkun
Dioprosopa clavatus	Argentina	Córdoba	Pedanía Los Reartes	-31.999126	-64.761418	Occurrence Download https://doi.org/10.15468/dl.3bxkun

Dioprosopa clavatus	United States of America	Florida	Manatee County	27.483163	-82.349133	Occurrence Download https://doi.org/10.15468/dl.3bxkun
Dioprosopa clavatus	Argentina	Santa Fe	San Jerónimo Norte	-31.550942	-61.06775	Occurrence Download https://doi.org/10.15468/dl.3bxkun
Dioprosopa clavatus	Argentina	Santa Fe	Esperanza	-31.453808	-60.925219	Occurrence Download https://doi.org/10.15468/dl.3bxkun
Dioprosopa clavatus	Argentina	Córdoba	Río Ceballos	-31.185156	-64.301259	Occurrence Download https://doi.org/10.15468/dl.3bxkun
Dioprosopa clavatus	United States of America	Florida	Miami	25.55876	-80.451905	Occurrence Download https://doi.org/10.15468/dl.3bxkun
Dioprosopa clavatus	Argentina	Santa Fe		-31.550796	-61.067578	Occurrence Download https://doi.org/10.15468/dl.3bxkun
Dioprosopa clavatus	Argentina	Santa Fe	San Jerónimo Norte	-31.550905	-61.06775	Occurrence Download https://doi.org/10.15468/dl.3bxkun
Dioprosopa clavatus	Argentina	Santa Fe	San Jerónimo Norte	-31.552734	-61.077921	Occurrence Download https://doi.org/10.15468/dl.3bxkun
Dioprosopa clavatus	Argentina	Santa Fe		-31.550832	-61.067621	Occurrence Download https://doi.org/10.15468/dl.3bxkun
Dioprosopa clavatus	Argentina	Santa Fe		-31.550869	-61.067621	Occurrence Download https://doi.org/10.15468/dl.3bxkun
Dioprosopa clavatus	Mexico	Querétaro	Higuerillas	20.8872	-99.7718	Occurrence Download https://doi.org/10.15468/dl.3bxkun
Dioprosopa clavatus	Argentina	Santa Fe	Esperanza	-31.453803	-60.925152	Occurrence Download https://doi.org/10.15468/dl.3bxkun
Dioprosopa clavatus	United States of America	Florida	Hernando County	28.59017	-82.49506	Occurrence Download https://doi.org/10.15468/dl.3bxkun
Dioprosopa clavatus	United States of America	Texas	Travis County	30.190472	-97.610066	Occurrence Download https://doi.org/10.15468/dl.3bxkun
Dioprosopa clavatus	United States of America	Texas	Hunt County	32.841892	-95.99366	Occurrence Download https://doi.org/10.15468/dl.3bxkun
Dioprosopa clavatus	United States of America	Florida	Sarasota County	27.249792	-82.474106	Occurrence Download https://doi.org/10.15468/dl.3bxkun
Dioprosopa clavatus	United States of America	Texas	McAllen	26.279746	-98.265698	Occurrence Download https://doi.org/10.15468/dl.3bxkun
Dioprosopa clavatus	United States of America	Florida	Miami-Dade County	25.763188	-80.499655	Occurrence Download https://doi.org/10.15468/dl.3bxkun
Dioprosopa clavatus	United States of America	Texas	Alvin	29.439854	-95.22461	Occurrence Download https://doi.org/10.15468/dl.3bxkun
Dioprosopa clavatus	United States of America	Florida	Sarasota County	27.19168	-82.443437	Occurrence Download https://doi.org/10.15468/dl.3bxkun
Dioprosopa clavatus	Bolivia	Santa Cruz	Municipio La Guardia	-17.96069	-63.197662	Occurrence Download https://doi.org/10.15468/dl.3bxkun
Dioprosopa clavatus	Argentina	Tucumán	Municipio de El Cadillal	-26.620413	-65.186983	Occurrence Download https://doi.org/10.15468/dl.3bxkun
Dioprosopa clavatus	United States of America	Texas	Brazoria County	29.540876	-95.378374	Occurrence Download https://doi.org/10.15468/dl.3bxkun
Dioprosopa clavatus	Mexico	Chiapas	San Cristóbal	16.706259	-92.615667	Occurrence Download https://doi.org/10.15468/dl.3bxkun
Dioprosopa clavatus	Mexico	Michoacán	Morelia	19.675003	-101.213615	Occurrence Download https://doi.org/10.15468/dl.3bxkun
Dioprosopa clavatus	United States of America	Florida	Miami-Dade County	25.391018	-80.689474	Occurrence Download https://doi.org/10.15468/dl.3bxkun
Dioprosopa clavatus	United States of America	Florida	Hendry County	26.31726	-80.881048	Occurrence Download https://doi.org/10.15468/dl.3bxkun
Dioprosopa clavatus	Brazil	Rio Grande do Sul	Triunfo	-29.865095	-51.687642	Occurrence Download https://doi.org/10.15468/dl.3bxkun
Dioprosopa clavatus	United States of America	Florida	Coral Gables	25.681675	-80.276664	Occurrence Download https://doi.org/10.15468/dl.3bxkun
Dioprosopa clavatus	United States of America	Texas	Cinco Ranch	29.722951	-95.744177	Occurrence Download https://doi.org/10.15468/dl.3bxkun
Dioprosopa clavatus	Mexico	Chiapas	San Cristóbal	16.706257	-92.615663	Occurrence Download https://doi.org/10.15468/dl.3bxkun
Dioprosopa clavatus	Peru	Lima	Surco	-11.887817	-76.444557	Occurrence Download https://doi.org/10.15468/dl.3bxkun
Dioprosopa clavatus	Mexico	Sinaloa	Culiacán	24.87838	-107.296303	Occurrence Download https://doi.org/10.15468/dl.3bxkun
Dioprosopa clavatus	Chile	Región Metropolitana de Santiago	San José de Maipo	-33.76589	-70.279135	Occurrence Download https://doi.org/10.15468/dl.3bxkun
Dioprosopa clavatus	United States of America	Texas	Alton	26.273162	-98.294528	Occurrence Download https://doi.org/10.15468/dl.3bxkun
Dioprosopa clavatus	Argentina	Entre Ríos	Basavilbaso	-32.377046	-58.872649	Occurrence Download https://doi.org/10.15468/dl.3bxkun
Dioprosopa clavatus	Argentina	Santa Fe		-31.474872	-60.944046	Occurrence Download https://doi.org/10.15468/dl.3bxkun
Dioprosopa clavatus	United States of America	Texas	Comal County	29.680214	-98.294652	Occurrence Download https://doi.org/10.15468/dl.3bxkun
Dioprosopa clavatus	United States of America	Florida	Okeechobee	27.228186	-80.851461	Occurrence Download https://doi.org/10.15468/dl.3bxkun

Dioprosopa clavatus	United States of America	Florida	Archbold	27.183306	-81.351778	Occurrence Download https://doi.org/10.15468/dl.3bxkun
Dioprosopa clavatus	United States of America	Florida	Yulee	30.627379	-81.583479	Occurrence Download https://doi.org/10.15468/dl.3bxkun
Dioprosopa clavatus	United States of America	Florida	Gainesville	29.75203	-82.368879	Occurrence Download https://doi.org/10.15468/dl.3bxkun
Dioprosopa clavatus	United States of America	Texas	Somervell County	32.208156	-97.648725	Occurrence Download https://doi.org/10.15468/dl.3bxkun
Dioprosopa clavatus	United States of America	Texas	Grand Prairie	32.588213	-97.040603	Occurrence Download https://doi.org/10.15468/dl.3bxkun
Dioprosopa clavatus	Argentina	Buenos Aires	Vedia	-34.4979	-61.542368	Occurrence Download https://doi.org/10.15468/dl.3bxkun
Dioprosopa clavatus	United States of America	Texas	Brewster County	29.242112	-103.296845	Occurrence Download https://doi.org/10.15468/dl.3bxkun
Dioprosopa clavatus	United States of America	Arizona	Cochise County	31.754533	-109.358268	Occurrence Download https://doi.org/10.15468/dl.3bxkun
Dioprosopa clavatus	United States of America	Alabama	Battens Crossroads	31.255	-85.90452	Occurrence Download https://doi.org/10.15468/dl.3bxkun
Dioprosopa clavatus	United States of America	Texas	Cinco Ranch	29.72207	-95.745835	Occurrence Download https://doi.org/10.15468/dl.3bxkun
Dioprosopa clavatus	United States of America	Texas	Alvin	29.440051	-95.224922	Occurrence Download https://doi.org/10.15468/dl.3bxkun
Dioprosopa clavatus	Mexico	Chihuahua	Meoqui	28.263588	-105.496662	Occurrence Download https://doi.org/10.15468/dl.3bxkun
Dioprosopa clavatus	Mexico	Chihuahua	Meoqui	28.258382	-105.503243	Occurrence Download https://doi.org/10.15468/dl.3bxkun
Dioprosopa clavatus	United States of America	Texas	Boerne	29.782108	-98.709221	Occurrence Download https://doi.org/10.15468/dl.3bxkun
Dioprosopa clavatus	United States of America	Arizona	Tucson	32.094251	-110.779289	Occurrence Download https://doi.org/10.15468/dl.3bxkun
Dioprosopa clavatus	United States of America	Florida	North Port	27.117765	-82.198246	Occurrence Download https://doi.org/10.15468/dl.3bxkun
Dioprosopa clavatus	United States of America	Florida	Jacksonville	30.14	-81.65	Occurrence Download https://doi.org/10.15468/dl.3bxkun
Dioprosopa clavatus	United States of America	Arizona	Village of Oak Creek	34.77142	-111.769745	Occurrence Download https://doi.org/10.15468/dl.3bxkun
Dioprosopa clavatus	United States of America	Texas	Collin County	33.258807	-96.651201	Occurrence Download https://doi.org/10.15468/dl.3bxkun
Dioprosopa clavatus	United States of America	Florida	Hernando County	28.590382	-82.495456	Occurrence Download https://doi.org/10.15468/dl.3bxkun
Dioprosopa clavatus	United States of America	Texas	Coryell County	31.346188	-97.789537	Occurrence Download https://doi.org/10.15468/dl.3bxkun
Dioprosopa clavatus	United States of America	Texas	Throckmorton County	33.169883	-99.003731	Occurrence Download https://doi.org/10.15468/dl.3bxkun
Dioprosopa clavatus	United States of America	Tennessee	Guthrie	36.641674	-87.156858	Occurrence Download https://doi.org/10.15468/dl.3bxkun
Dioprosopa clavatus	Argentina	Córdoba	Los Quebrachitos	-31.196018	-64.366174	Occurrence Download https://doi.org/10.15468/dl.3bxkun
Dioprosopa clavatus	Argentina	Chaco	Municipio de El Sauzalito	-25.179007	-61.096533	Occurrence Download https://doi.org/10.15468/dl.3bxkun
Dioprosopa clavatus	United States of America	Florida	Sarasota County	27.32543	-82.432721	Occurrence Download https://doi.org/10.15468/dl.3bxkun
Dioprosopa clavatus	Mexico	Michoacán	Morelia	19.684541	-101.236383	Occurrence Download https://doi.org/10.15468/dl.3bxkun
Dioprosopa clavatus	United States of America	Florida	DeBary	28.884942	-81.301382	Occurrence Download https://doi.org/10.15468/dl.3bxkun
Dioprosopa clavatus	United States of America	Texas	Throckmorton County	33.169813	-99.003656	Occurrence Download https://doi.org/10.15468/dl.3bxkun
Dioprosopa clavatus	United States of America	Kansas	Lenexa	38.978724	-94.864628	Occurrence Download https://doi.org/10.15468/dl.3bxkun
Dioprosopa clavatus	United States of America	Texas	Cinco Ranch	29.722059	-95.745838	Occurrence Download https://doi.org/10.15468/dl.3bxkun
Dioprosopa clavatus	United States of America	California	Laguna Beach	33.541386	-117.779631	Occurrence Download https://doi.org/10.15468/dl.3bxkun
Dioprosopa clavatus	United States of America	Arizona	Pima County	31.784042	-110.646662	Occurrence Download https://doi.org/10.15468/dl.3bxkun
Dioprosopa clavatus	United States of America	Texas	Burnet	30.85007	-98.133544	Occurrence Download https://doi.org/10.15468/dl.3bxkun
Dioprosopa clavatus	United States of America	Texas	Alvin	29.439905	-95.225392	Occurrence Download https://doi.org/10.15468/dl.3bxkun
Dioprosopa clavatus	United States of America	Florida	Collier County	26.375662	-81.603828	Occurrence Download https://doi.org/10.15468/dl.3bxkun
Dioprosopa clavatus	Mexico	Sinaloa	San Manuel	24.348786	-107.372614	Occurrence Download https://doi.org/10.15468/dl.3bxkun
Dioprosopa clavatus	United States of America	Texas	Fort Bend County	29.367262	-95.823238	Occurrence Download https://doi.org/10.15468/dl.3bxkun
Dioprosopa clavatus	Bolivia	Santa Cruz	Municipio Santa Cruz de la Sierra	-17.736478	-63.115881	Occurrence Download https://doi.org/10.15468/dl.3bxkun

Dioprosopa clavatus	United States of America	Texas	Waterside Estates	29.651166	-95.731032	Occurrence Download	https://doi.org/10.15468/dl.3bxkun
Dioprosopa clavatus	United States of America	Hawaii	Waimanalo Beach	21.342144	-157.7017	Occurrence Download	https://doi.org/10.15468/dl.3bxkun
Dioprosopa clavatus	United States of America	Texas	Sterling Creek	29.972167	-95.201997	Occurrence Download	https://doi.org/10.15468/dl.3bxkun
Dioprosopa clavatus	United States of America	Texas	Oglesby	31.421158	-97.507661	Occurrence Download	https://doi.org/10.15468/dl.3bxkun
Dioprosopa clavatus	United States of America	Florida	Bradford County	29.871509	-82.072251	Occurrence Download	https://doi.org/10.15468/dl.3bxkun
Dioprosopa clavatus	United States of America	Florida	Bradford County	29.955032	-82.169366	Occurrence Download	https://doi.org/10.15468/dl.3bxkun
Dioprosopa clavatus	United States of America	Arizona	Maricopa County	33.510162	-111.858817	Occurrence Download	https://doi.org/10.15468/dl.3bxkun
Dioprosopa clavatus	United States of America	Florida	Bradford County	29.929949	-82.162834	Occurrence Download	https://doi.org/10.15468/dl.3bxkun
Dioprosopa clavatus	United States of America	Texas	Medina County	29.551582	-98.855259	Occurrence Download	https://doi.org/10.15468/dl.3bxkun
Dioprosopa clavatus	United States of America	Arizona	Soldier Camp	32.425951	-110.748457	Occurrence Download	https://doi.org/10.15468/dl.3bxkun
Dioprosopa clavatus	United States of America	Arizona	Pima County	31.787645	-110.636091	Occurrence Download	https://doi.org/10.15468/dl.3bxkun
Dioprosopa clavatus	United States of America	Texas	Sugar Land	29.564469	-95.642844	Occurrence Download	https://doi.org/10.15468/dl.3bxkun
Dioprosopa clavatus	United States of America	Texas	Ennis	32.312298	-96.631557	Occurrence Download	https://doi.org/10.15468/dl.3bxkun
Dioprosopa clavatus	United States of America	Texas	Bastrop County	30.163681	-97.183228	Occurrence Download	https://doi.org/10.15468/dl.3bxkun
Dioprosopa clavatus	United States of America	Virginia	York County	37.23736	-76.578135	Occurrence Download	https://doi.org/10.15468/dl.3bxkun
Dioprosopa clavatus	United States of America	Arizona	Pima County	32.281833	-110.730775	Occurrence Download	https://doi.org/10.15468/dl.3bxkun
Dioprosopa clavatus	United States of America	Florida	Hendry County	26.288735	-80.888518	Occurrence Download	https://doi.org/10.15468/dl.3bxkun
Dioprosopa clavatus	United States of America	Florida	Hillsborough County	27.824858	-82.312276	Occurrence Download	https://doi.org/10.15468/dl.3bxkun
Dioprosopa clavatus	United States of America	Florida	Sarasota County	27.325454	-82.432688	Occurrence Download	https://doi.org/10.15468/dl.3bxkun
Dioprosopa clavatus	United States of America	Texas	Wise County	33.30915	-97.607497	Occurrence Download	https://doi.org/10.15468/dl.3bxkun
Dioprosopa clavatus	United States of America	Florida	Osceola County	28.347058	-81.195023	Occurrence Download	https://doi.org/10.15468/dl.3bxkun
Dioprosopa clavatus	United States of America	Florida	Escambia County	30.45425	-87.343071	Occurrence Download	https://doi.org/10.15468/dl.3bxkun
Dioprosopa clavatus	United States of America	Virginia	Gloucester County	37.396664	-76.598792	Occurrence Download	https://doi.org/10.15468/dl.3bxkun
Dioprosopa clavatus	United States of America	Texas	Gonzales	29.486864	-97.45196	Occurrence Download	https://doi.org/10.15468/dl.3bxkun
Dioprosopa clavatus	United States of America	Kansas	Lawrence	38.951437	-95.215622	Occurrence Download	https://doi.org/10.15468/dl.3bxkun
Dioprosopa clavatus	United States of America	Florida	Sarasota County	27.123217	-82.352383	Occurrence Download	https://doi.org/10.15468/dl.3bxkun
Dioprosopa clavatus	United States of America	Florida	Sarasota County	27.325409	-82.432729	Occurrence Download	https://doi.org/10.15468/dl.3bxkun
Dioprosopa clavatus	United States of America	Louisiana	St. George	30.357029	-91.095111	Occurrence Download	https://doi.org/10.15468/dl.3bxkun
Dioprosopa clavatus	United States of America	Louisiana	St. George	30.365965	-91.082598	Occurrence Download	https://doi.org/10.15468/dl.3bxkun
Dioprosopa clavatus	United States of America	Florida	Osprey	27.196497	-82.483936	Occurrence Download	https://doi.org/10.15468/dl.3bxkun
Dioprosopa clavatus	United States of America	Texas	Cameron County	26.161635	-97.794207	Occurrence Download	https://doi.org/10.15468/dl.3bxkun
Dioprosopa clavatus	United States of America	Texas	Burnet County	30.54401	-98.244445	Occurrence Download	https://doi.org/10.15468/dl.3bxkun
Dioprosopa clavatus	United States of America	New York	City of New York	40.736639	-74.010301	Occurrence Download	https://doi.org/10.15468/dl.3bxkun
Dioprosopa clavatus	United States of America	Texas	Temple	31.06093	-97.38045	Occurrence Download	https://doi.org/10.15468/dl.3bxkun
Dioprosopa clavatus	United States of America	Texas	New Braunfels	29.64149	-98.128243	Occurrence Download	https://doi.org/10.15468/dl.3bxkun
Dioprosopa clavatus	United States of America	Texas	Collin County	33.136375	-96.562449	Occurrence Download	https://doi.org/10.15468/dl.3bxkun
Dioprosopa clavatus	United States of America	Maryland	Snow Hill	38.176262	-75.390075	Occurrence Download	https://doi.org/10.15468/dl.3bxkun
Dioprosopa clavatus	United States of America	Texas	Harris County	29.851895	-95.726913	Occurrence Download	https://doi.org/10.15468/dl.3bxkun
Dioprosopa clavatus	United States of America	New York	Village of Lansing	42.480534	-76.449419	Occurrence Download	https://doi.org/10.15468/dl.3bxkun

Dioprosopa clavatus	United States of America	Alabama	Burnout	34.458213	-88.030272	Occurrence Download https://doi.org/10.15468/dl.3bxkun
Dioprosopa clavatus	United States of America	Kentucky	Burgin	37.757518	-84.791117	Occurrence Download https://doi.org/10.15468/dl.3bxkun
Dioprosopa clavatus	United States of America	Texas	Austin	30.185571	-97.873814	Occurrence Download https://doi.org/10.15468/dl.3bxkun
Dioprosopa clavatus	United States of America	Texas	Antioch	30.122306	-97.159522	Occurrence Download https://doi.org/10.15468/dl.3bxkun
Dioprosopa clavatus	United States of America	Florida	Waldo	29.749912	-82.22345	Occurrence Download https://doi.org/10.15468/dl.3bxkun
Dioprosopa clavatus	United States of America	New Mexico	Lea County	32.809237	-103.75149	Occurrence Download https://doi.org/10.15468/dl.3bxkun
Dioprosopa clavatus	United States of America	Florida	Hillsborough County	27.824882	-82.31228	Occurrence Download https://doi.org/10.15468/dl.3bxkun
Dioprosopa clavatus	United States of America	Texas	San Antonio	29.421493	-98.370899	Occurrence Download https://doi.org/10.15468/dl.3bxkun
Dioprosopa clavatus	United States of America	Texas	Saint Hedwig	29.410242	-98.245115	Occurrence Download https://doi.org/10.15468/dl.3bxkun
Dioprosopa clavatus	United States of America	Texas	Oglesby	31.421203	-97.507619	Occurrence Download https://doi.org/10.15468/dl.3bxkun
Dioprosopa clavatus	United States of America	Texas	Hidalgo County	26.276574	-98.531921	Occurrence Download https://doi.org/10.15468/dl.3bxkun
Dioprosopa clavatus	Mexico	Durango	Gómez Palacio	25.598386	-103.454534	Occurrence Download https://doi.org/10.15468/dl.3bxkun
Dioprosopa clavatus	United States of America	Illinois	McDonough County	40.477073	-90.743038	Occurrence Download https://doi.org/10.15468/dl.3bxkun
Dioprosopa clavatus	Brazil	Paraíba	Areia	-6.970938	-35.71246	Occurrence Download https://doi.org/10.15468/dl.3bxkun
Dioprosopa clavatus	Mexico	Chihuahua	Meoqui	28.261787	-105.496109	Occurrence Download https://doi.org/10.15468/dl.3bxkun
Dioprosopa clavatus	Mexico	Chihuahua	Meoqui	28.263164	-105.497199	Occurrence Download https://doi.org/10.15468/dl.3bxkun
Dioprosopa clavatus	United States of America	Arkansas	Fayetteville	36.074815	-94.24725	Occurrence Download https://doi.org/10.15468/dl.3bxkun
Dioprosopa clavatus	Mexico	Querétaro	Cadereyta de Montes	20.685806	-99.803331	Occurrence Download https://doi.org/10.15468/dl.3bxkun
Dioprosopa clavatus	Honduras	Atlántida	Tela	15.767678	-87.456955	Occurrence Download https://doi.org/10.15468/dl.3bxkun
Dioprosopa clavatus	United States of America	Florida	Eagle Lake	27.970437	-81.762833	Occurrence Download https://doi.org/10.15468/dl.3bxkun
Dioprosopa clavatus	United States of America	Texas	Austin	30.366795	-97.860632	Occurrence Download https://doi.org/10.15468/dl.3bxkun
Dioprosopa clavatus	United States of America	Texas	Travis County	30.167038	-97.874038	Occurrence Download https://doi.org/10.15468/dl.3bxkun
Dioprosopa clavatus	United States of America	Texas	Colorado County	29.674475	-96.287728	Occurrence Download https://doi.org/10.15468/dl.3bxkun
Dioprosopa clavatus	United States of America	Texas	Oglesby	31.421214	-97.507664	Occurrence Download https://doi.org/10.15468/dl.3bxkun
Dioprosopa clavatus	United States of America	Texas	Cypress	29.993362	-95.71212	Occurrence Download https://doi.org/10.15468/dl.3bxkun
Dioprosopa clavatus	United States of America	Texas	Bascom	32.307607	-95.216037	Occurrence Download https://doi.org/10.15468/dl.3bxkun
Dioprosopa clavatus	United States of America	Texas	Hood County	32.475351	-97.857203	Occurrence Download https://doi.org/10.15468/dl.3bxkun
Dioprosopa clavatus	United States of America	Texas	College Station	30.547725	-96.29888	Occurrence Download https://doi.org/10.15468/dl.3bxkun
Dioprosopa clavatus	United States of America	Texas	Stockdale	29.240703	-97.962862	Occurrence Download https://doi.org/10.15468/dl.3bxkun
Dioprosopa clavatus	Mexico	Guanajuato	Irapuato	20.661171	-101.382008	Occurrence Download https://doi.org/10.15468/dl.3bxkun
Dioprosopa clavatus	United States of America	Alabama	Woodland Mills	34.46957	-86.657566	Occurrence Download https://doi.org/10.15468/dl.3bxkun
Dioprosopa clavatus	United States of America	Texas	Jackson County	28.803922	-96.336324	Occurrence Download https://doi.org/10.15468/dl.3bxkun
Dioprosopa clavatus	United States of America	Texas	San Marcos	29.853825	-97.930757	Occurrence Download https://doi.org/10.15468/dl.3bxkun
Dioprosopa clavatus	United States of America	Oklahoma	Sapulpa	35.99008	-96.12075	Occurrence Download https://doi.org/10.15468/dl.3bxkun
Dioprosopa clavatus	United States of America	New Mexico	Eddy County	32.11046	-104.464089	Occurrence Download https://doi.org/10.15468/dl.3bxkun
Dioprosopa clavatus	Mexico	Chihuahua	Tesoro Escondido	31.607537	-106.541345	Occurrence Download https://doi.org/10.15468/dl.3bxkun
Dioprosopa clavatus	United States of America	North Carolina	Durham County	36.165347	-78.859767	Occurrence Download https://doi.org/10.15468/dl.3bxkun
Dioprosopa clavatus	United States of America	Texas	Sugar Land	29.565422	-95.64725	Occurrence Download https://doi.org/10.15468/dl.3bxkun
Dioprosopa clavatus	United States of America	Texas	Montgomery County	30.22333	-95.236917	Occurrence Download https://doi.org/10.15468/dl.3bxkun

Dioprosopa clavatus	United States of America	New York	City of New York	40.767242	-73.944733	Occurrence Download	https://doi.org/10.15468/dl.3bxkun
Dioprosopa clavatus	United States of America	Texas	Montgomery County	30.22333	-95.23693	Occurrence Download	https://doi.org/10.15468/dl.3bxkun
Dioprosopa clavatus	United States of America	Delaware	Kent County	39.044204	-75.527218	Occurrence Download	https://doi.org/10.15468/dl.3bxkun
Dioprosopa clavatus	United States of America	Louisiana	St. George	30.35668	-91.095037	Occurrence Download	https://doi.org/10.15468/dl.3bxkun
Dioprosopa clavatus	Mexico	Veracruz	Agua Dulce	18.137524	-94.131816	Occurrence Download	https://doi.org/10.15468/dl.3bxkun
Dioprosopa clavatus	Mexico	Querétaro	Cadereyta de Montes	20.68466	-99.803171	Occurrence Download	https://doi.org/10.15468/dl.3bxkun
Dioprosopa clavatus	Mexico	Nuevo León	Monterrey	25.542071	-100.271444	Occurrence Download	https://doi.org/10.15468/dl.3bxkun
Dioprosopa clavatus	United States of America	Tennessee	Clarksville	36.619361	-87.33851	Occurrence Download	https://doi.org/10.15468/dl.3bxkun
Dioprosopa clavatus	United States of America	Kansas	Salina	38.810845	-97.587101	Occurrence Download	https://doi.org/10.15468/dl.3bxkun
Dioprosopa clavatus	United States of America	Pennsylvania	Atglen	39.945553	-75.971062	Occurrence Download	https://doi.org/10.15468/dl.3bxkun
Dioprosopa clavatus	United States of America	North Carolina	Swannanoa	35.597894	-82.399843	Occurrence Download	https://doi.org/10.15468/dl.3bxkun
Dioprosopa clavatus	United States of America	Tennessee	Warren County	35.61692	-85.859558	Occurrence Download	https://doi.org/10.15468/dl.3bxkun
Dioprosopa clavatus	United States of America	Missouri	Greene County	37.416045	-93.348554	Occurrence Download	https://doi.org/10.15468/dl.3bxkun
Dioprosopa clavatus	United States of America	Illinois	Knox County	40.822384	-90.118908	Occurrence Download	https://doi.org/10.15468/dl.3bxkun
Dioprosopa clavatus	Mexico	Sinaloa	Culiacán	24.76067	-107.462606	Occurrence Download	https://doi.org/10.15468/dl.3bxkun
Dioprosopa clavatus	United States of America	Delaware	Kent County	39.044542	-75.527478	Occurrence Download	https://doi.org/10.15468/dl.3bxkun
Dioprosopa clavatus	United States of America	Texas	Grayson County	33.448353	-96.709	Occurrence Download	https://doi.org/10.15468/dl.3bxkun
Dioprosopa clavatus	United States of America	Missouri	Greene County	37.415122	-93.372232	Occurrence Download	https://doi.org/10.15468/dl.3bxkun
Dioprosopa clavatus	United States of America	Missouri	Castlewood	38.550632	-90.555283	Occurrence Download	https://doi.org/10.15468/dl.3bxkun
Dioprosopa clavatus	United States of America	Indiana	Tippecanoe County	40.335525	-86.865039	Occurrence Download	https://doi.org/10.15468/dl.3bxkun
Dioprosopa clavatus	United States of America	Louisiana	Bossier Parish	32.612684	-93.73303	Occurrence Download	https://doi.org/10.15468/dl.3bxkun
Dioprosopa clavatus	Mexico	Guanajuato	Acámbaro	19.965693	-100.706955	Occurrence Download	https://doi.org/10.15468/dl.3bxkun
Dioprosopa clavatus	United States of America	South Carolina	Johns Island	32.735869	-80.022056	Occurrence Download	https://doi.org/10.15468/dl.3bxkun
Dioprosopa clavatus	United States of America	Florida	Cantonment	30.599777	-87.3474	Occurrence Download	https://doi.org/10.15468/dl.3bxkun
Dioprosopa clavatus	Mexico	Sinaloa	Culiacán	24.761951	-107.465967	Occurrence Download	https://doi.org/10.15468/dl.3bxkun
Dioprosopa clavatus	Brazil	Paraíba	Alagoa Grande	-7.117621	-35.525163	Occurrence Download	https://doi.org/10.15468/dl.3bxkun
Dioprosopa clavatus	Brazil	Paraíba	Alagoa Grande	-7.117925	-35.524716	Occurrence Download	https://doi.org/10.15468/dl.3bxkun
Dioprosopa clavatus	United States of America	New York	City of New York	40.767493	-73.944584	Occurrence Download	https://doi.org/10.15468/dl.3bxkun
Dioprosopa clavatus	Brazil	Ceará	Barbalha	-7.277607	-39.281314	Occurrence Download	https://doi.org/10.15468/dl.3bxkun
Dioprosopa clavatus	United States of America	Missouri	Greene County	37.16983	-93.360767	Occurrence Download	https://doi.org/10.15468/dl.3bxkun
Dioprosopa clavatus	United States of America	Texas	Travis County	30.158317	-97.869522	Occurrence Download	https://doi.org/10.15468/dl.3bxkun
Dioprosopa clavatus	United States of America	Virginia	Cape Charles	37.268383	-76.016139	Occurrence Download	https://doi.org/10.15468/dl.3bxkun
Dioprosopa clavatus	United States of America	Florida	Martin County	27.112492	-80.285936	Occurrence Download	https://doi.org/10.15468/dl.3bxkun
Dioprosopa clavatus	United States of America	Mississippi	Monroe County	33.810354	-88.43946	Occurrence Download	https://doi.org/10.15468/dl.3bxkun
Dioprosopa clavatus	United States of America	California	Redlands	34.031092	-117.195051	Occurrence Download	https://doi.org/10.15468/dl.3bxkun
Dioprosopa clavatus	United States of America	Kansas	Atchison	39.553813	-95.125375	Occurrence Download	https://doi.org/10.15468/dl.3bxkun
Dioprosopa clavatus	United States of America	Texas	College Station	30.56185	-96.27673	Occurrence Download	https://doi.org/10.15468/dl.3bxkun
Dioprosopa clavatus	United States of America	Arkansas	Barling	35.325311	-94.301375	Occurrence Download	https://doi.org/10.15468/dl.3bxkun
Dioprosopa clavatus	United States of America	Arkansas	Bella Vista	36.45292	-94.185824	Occurrence Download	https://doi.org/10.15468/dl.3bxkun

Dioprosopa clavatus	United States of America	Texas	Sugar Land	29.564894	-95.646635	Occurrence Download https://doi.org/10.15468/dl.3bxkun
Dioprosopa clavatus	United States of America	Texas	Grayson County	33.7853	-96.904831	Occurrence Download https://doi.org/10.15468/dl.3bxkun
Dioprosopa clavatus	United States of America	California	San Diego County	32.888871	-116.803747	Occurrence Download https://doi.org/10.15468/dl.3bxkun
Dioprosopa clavatus	Mexico	Chihuahua	Meoqui	28.263359	-105.493051	Occurrence Download https://doi.org/10.15468/dl.3bxkun
Dioprosopa clavatus	United States of America	Texas	Johnson County	32.418942	-97.551047	Occurrence Download https://doi.org/10.15468/dl.3bxkun
Dioprosopa clavatus	United States of America	Texas	Rowlett	32.947007	-96.533878	Occurrence Download https://doi.org/10.15468/dl.3bxkun
Dioprosopa clavatus	United States of America	Texas	Fairview	32.502598	-97.811789	Occurrence Download https://doi.org/10.15468/dl.3bxkun
Dioprosopa clavatus	United States of America	Oklahoma	Jones	35.561576	-97.277522	Occurrence Download https://doi.org/10.15468/dl.3bxkun
Dioprosopa clavatus	United States of America	Louisiana	Bankers	30.148092	-91.7725	Occurrence Download https://doi.org/10.15468/dl.3bxkun
Dioprosopa clavatus	Mexico	Jalisco	Santa Ana Tepetitlán	20.604478	-103.480939	Occurrence Download https://doi.org/10.15468/dl.3bxkun
Dioprosopa clavatus	United States of America	Louisiana	Ramsay	30.522518	-90.126849	Occurrence Download https://doi.org/10.15468/dl.3bxkun
Dioprosopa clavatus	United States of America	Alabama	Auburn	32.58	-85.49	Occurrence Download https://doi.org/10.15468/dl.3bxkun
Dioprosopa clavatus	United States of America	Georgia	Pickens County	34.404438	-84.493912	Occurrence Download https://doi.org/10.15468/dl.3bxkun
Dioprosopa clavatus	United States of America	Florida	Brevard County	28.6924	-80.846969	Occurrence Download https://doi.org/10.15468/dl.3bxkun
Dioprosopa clavatus	United States of America	Texas	Sugar Land	29.564883	-95.646608	Occurrence Download https://doi.org/10.15468/dl.3bxkun
Dioprosopa clavatus	United States of America	Texas	Hood County	32.44398	-97.999855	Occurrence Download https://doi.org/10.15468/dl.3bxkun
Dioprosopa clavatus	United States of America	Texas	Fort Bend County	29.554561	-95.585411	Occurrence Download https://doi.org/10.15468/dl.3bxkun
Dioprosopa clavatus	United States of America	Texas	Johnson County	32.419025	-97.551047	Occurrence Download https://doi.org/10.15468/dl.3bxkun
Dioprosopa clavatus	United States of America	Indiana	Jeffersonville	38.391842	-85.662247	Occurrence Download https://doi.org/10.15468/dl.3bxkun
Dioprosopa clavatus	United States of America	Texas	Stockdale	29.24052	-97.962778	Occurrence Download https://doi.org/10.15468/dl.3bxkun
Dioprosopa clavatus	United States of America	Georgia	Gray	33.00089	-83.528993	Occurrence Download https://doi.org/10.15468/dl.3bxkun
Dioprosopa clavatus	United States of America	California	Santa Clara County	37.369593	-121.734857	Occurrence Download https://doi.org/10.15468/dl.3bxkun
Dioprosopa clavatus	United States of America	Texas	Georgetown	30.689978	-97.646972	Occurrence Download https://doi.org/10.15468/dl.3bxkun
Dioprosopa clavatus	United States of America	California	Atwater	37.333397	-120.578381	Occurrence Download https://doi.org/10.15468/dl.3bxkun
Dioprosopa clavatus	United States of America	California	Merced County	37.398797	-120.430436	Occurrence Download https://doi.org/10.15468/dl.3bxkun
Dioprosopa clavatus	United States of America	Texas	Abernathy	33.812917	-101.804097	Occurrence Download https://doi.org/10.15468/dl.3bxkun
Dioprosopa clavatus	United States of America	North Carolina	Durham	35.842349	-78.924575	Occurrence Download https://doi.org/10.15468/dl.3bxkun
Dioprosopa clavatus	United States of America	Texas	Lamar County	33.646752	-95.349518	Occurrence Download https://doi.org/10.15468/dl.3bxkun
Dioprosopa clavatus	United States of America	Maryland	Queen Anne Hill	38.738611	-76.556667	Occurrence Download https://doi.org/10.15468/dl.3bxkun
Dioprosopa clavatus	United States of America	Texas	Midlothian	32.456893	-96.982737	Occurrence Download https://doi.org/10.15468/dl.3bxkun
Dioprosopa clavatus	United States of America	Texas	Jefferson County	30.058772	-94.292847	Occurrence Download https://doi.org/10.15468/dl.3bxkun
Dioprosopa clavatus	United States of America	California	San Diego County	32.945143	-116.920056	Occurrence Download https://doi.org/10.15468/dl.3bxkun
Dioprosopa clavatus	United States of America	Georgia	Bluff Creek	34.127817	-84.449065	Occurrence Download https://doi.org/10.15468/dl.3bxkun
Dioprosopa clavatus	United States of America	Georgia	Bluff Creek	34.127903	-84.449078	Occurrence Download https://doi.org/10.15468/dl.3bxkun
Dioprosopa clavatus	United States of America	Georgia	Rockdale County	33.584823	-84.068002	Occurrence Download https://doi.org/10.15468/dl.3bxkun
Dioprosopa clavatus	United States of America	Florida	Hampton	29.850228	-82.089822	Occurrence Download https://doi.org/10.15468/dl.3bxkun
Dioprosopa clavatus	United States of America	California	San Diego County	32.92844	-116.812852	Occurrence Download https://doi.org/10.15468/dl.3bxkun
Dioprosopa clavatus	United States of America	South Carolina	Berkeley County	33.166875	-79.532363	Occurrence Download https://doi.org/10.15468/dl.3bxkun
Dioprosopa clavatus	United States of America	Texas	Winscott	32.555963	-97.526762	Occurrence Download https://doi.org/10.15468/dl.3bxkun

Dioprosopa clavatus	United States of America	Alabama	Jefferson County	33.760405	-86.67879	Occurrence Download https://doi.org/10.15468/dl.3bxkun
Dioprosopa clavatus	United States of America	Georgia	Bluff Creek	34.128047	-84.448835	Occurrence Download https://doi.org/10.15468/dl.3bxkun
Dioprosopa clavatus	Mexico	Nuevo León	Guadalupe	25.668997	-100.135612	Occurrence Download https://doi.org/10.15468/dl.3bxkun
Dioprosopa clavatus	United States of America	Arizona	Cornville	34.763248	-111.894695	Occurrence Download https://doi.org/10.15468/dl.3bxkun
Dioprosopa clavatus	Mexico	Nuevo León	Monterrey	25.549845	-100.270427	Occurrence Download https://doi.org/10.15468/dl.3bxkun
Dioprosopa clavatus	United States of America	Texas	San Marcos	29.905418	-97.896944	Occurrence Download https://doi.org/10.15468/dl.3bxkun
Dioprosopa clavatus	United States of America	Arkansas	Erbie	36.060505	-93.256316	Occurrence Download https://doi.org/10.15468/dl.3bxkun
Dioprosopa clavatus	United States of America	California	Los Angeles	33.722351	-118.305912	Occurrence Download https://doi.org/10.15468/dl.3bxkun
Dioprosopa clavatus	United States of America	Florida	Marion County	29.211347	-81.909058	Occurrence Download https://doi.org/10.15468/dl.3bxkun
Dioprosopa clavatus	United States of America	Texas	Lucas	33.075167	-96.548569	Occurrence Download https://doi.org/10.15468/dl.3bxkun
Dioprosopa clavatus	United States of America	California	San Diego	32.89809	-117.056716	Occurrence Download https://doi.org/10.15468/dl.3bxkun
Dioprosopa clavatus	United States of America	Texas	San Antonio	29.251157	-98.546854	Occurrence Download https://doi.org/10.15468/dl.3bxkun
Dioprosopa clavatus	United States of America	California	San Diego	32.922961	-117.041008	Occurrence Download https://doi.org/10.15468/dl.3bxkun
Dioprosopa clavatus	United States of America	Florida	Jacksonville	30.202439	-81.861653	Occurrence Download https://doi.org/10.15468/dl.3bxkun
Dioprosopa clavatus	United States of America	Texas	Singleton	30.6545	-95.9637	Occurrence Download https://doi.org/10.15468/dl.3bxkun
Dioprosopa clavatus	United States of America	Texas	Denton County	33.136386	-97.151397	Occurrence Download https://doi.org/10.15468/dl.3bxkun
Dioprosopa clavatus	United States of America	North Carolina	Grandy	36.261142	-75.885812	Occurrence Download https://doi.org/10.15468/dl.3bxkun
Dioprosopa clavatus	United States of America	Texas	Fort Worth	32.90201	-97.35806	Occurrence Download https://doi.org/10.15468/dl.3bxkun
Dioprosopa clavatus	United States of America	Delaware	Buck Hill Landing	38.56322	-75.221626	Occurrence Download https://doi.org/10.15468/dl.3bxkun
Dioprosopa clavatus	United States of America	Texas	Hays County	29.848295	-97.988756	Occurrence Download https://doi.org/10.15468/dl.3bxkun
Dioprosopa clavatus	Mexico	Chihuahua	Meoqui	28.258893	-105.503209	Occurrence Download https://doi.org/10.15468/dl.3bxkun
Dioprosopa clavatus	United States of America	California	Jamul	32.675775	-116.705191	Occurrence Download https://doi.org/10.15468/dl.3bxkun
Dioprosopa clavatus	United States of America	Texas	Palmview South	26.2173	-98.376532	Occurrence Download https://doi.org/10.15468/dl.3bxkun
Dioprosopa clavatus	United States of America	New Mexico	Mesilla	32.283914	-106.825676	Occurrence Download https://doi.org/10.15468/dl.3bxkun
Dioprosopa clavatus	United States of America	Florida	Orlando	28.419014	-81.236248	Occurrence Download https://doi.org/10.15468/dl.3bxkun
Dioprosopa clavatus	Mexico	Campeche	Campeche	19.827781	-90.53001	Occurrence Download https://doi.org/10.15468/dl.3bxkun
Dioprosopa clavatus	United States of America	Texas	Edinburg	26.29065	-98.135282	Occurrence Download https://doi.org/10.15468/dl.3bxkun
Dioprosopa clavatus	Mexico	Oaxaca	Puerto Ángel	15.670294	-96.485785	Occurrence Download https://doi.org/10.15468/dl.3bxkun
Dioprosopa clavatus	Mexico	Tamaulipas	Ciudad Tula	22.990656	-99.72048	Occurrence Download https://doi.org/10.15468/dl.3bxkun
Dioprosopa clavatus	United States of America	Texas	Weatherford	32.720294	-97.788122	Occurrence Download https://doi.org/10.15468/dl.3bxkun
Dioprosopa clavatus	United States of America	Texas	Kingsville	27.524283	-97.880867	Occurrence Download https://doi.org/10.15468/dl.3bxkun
Dioprosopa clavatus	United States of America	Texas	Bandera County	29.628055	-99.181044	Occurrence Download https://doi.org/10.15468/dl.3bxkun
Dioprosopa clavatus	United States of America	Texas	Harker Heights	31.066218	-97.671855	Occurrence Download https://doi.org/10.15468/dl.3bxkun
Dioprosopa clavatus	Mexico	Nuevo León	Monterrey	25.550052	-100.27039	Occurrence Download https://doi.org/10.15468/dl.3bxkun
Dioprosopa clavatus	United States of America	Texas	Austin	30.33932	-97.891983	Occurrence Download https://doi.org/10.15468/dl.3bxkun
Dioprosopa clavatus	United States of America	North Carolina	Grandy	36.261112	-75.885895	Occurrence Download https://doi.org/10.15468/dl.3bxkun
Dioprosopa clavatus	United States of America	North Carolina	Currituck County	36.260722	-75.886397	Occurrence Download https://doi.org/10.15468/dl.3bxkun
Dioprosopa clavatus	United States of America	Maine	Wells	43.293108	-70.569298	Occurrence Download https://doi.org/10.15468/dl.3bxkun
Dioprosopa clavatus	United States of America	Kentucky	Louisville	38.362398	-85.631611	Occurrence Download https://doi.org/10.15468/dl.3bxkun

Dioprosopa clavatus	United States of America	Georgia	Jones County	32.941858	-83.420528	Occurrence Download https://doi.org/10.15468/dl.3bxkun
Dioprosopa clavatus	United States of America	Oklahoma	Osage County	36.206864	-96.062136	Occurrence Download https://doi.org/10.15468/dl.3bxkun
Dioprosopa clavatus	United States of America	Texas	Edinburg	26.290387	-98.136167	Occurrence Download https://doi.org/10.15468/dl.3bxkun
Dioprosopa clavatus	United States of America	North Carolina	Currituck County	36.259297	-75.889205	Occurrence Download https://doi.org/10.15468/dl.3bxkun
Dioprosopa clavatus	United States of America	Georgia	Jones County	32.94283	-83.419036	Occurrence Download https://doi.org/10.15468/dl.3bxkun
Dioprosopa clavatus	United States of America	Missouri	Joplin	37.050303	-94.530455	Occurrence Download https://doi.org/10.15468/dl.3bxkun
Dioprosopa clavatus	United States of America	Texas	Mission	26.17731	-98.366098	Occurrence Download https://doi.org/10.15468/dl.3bxkun
Dioprosopa clavatus	United States of America	California	San Diego County	32.660632	-116.851909	Occurrence Download https://doi.org/10.15468/dl.3bxkun
Dioprosopa clavatus	United States of America	Texas	Mission	26.180017	-98.366768	Occurrence Download https://doi.org/10.15468/dl.3bxkun
Dioprosopa clavatus	United States of America	Texas	Brazoria County	29.094889	-95.279678	Occurrence Download https://doi.org/10.15468/dl.3bxkun
Dioprosopa clavatus	Argentina	Córdoba	Villa Tulumba	-30.348989	-64.08914	Occurrence Download https://doi.org/10.15468/dl.3bxkun
Dioprosopa clavatus	United States of America	Georgia	Euchee Creek	33.465863	-82.230181	Occurrence Download https://doi.org/10.15468/dl.3bxkun
Dioprosopa clavatus	Mexico	Quintana Roo	Cancún	21.133664	-86.83615	Occurrence Download https://doi.org/10.15468/dl.3bxkun
Dioprosopa clavatus	United States of America	Florida	Groveland	28.511106	-81.841911	Occurrence Download https://doi.org/10.15468/dl.3bxkun
Dioprosopa clavatus	Brazil	Minas Gerais	Santa Luzia	-19.812124	-43.857852	Occurrence Download https://doi.org/10.15468/dl.3bxkun
Dioprosopa clavatus	Mexico	Jalisco	Magdalena	20.901242	-104.004535	Occurrence Download https://doi.org/10.15468/dl.3bxkun
Dioprosopa clavatus	United States of America	Oklahoma	Norman	35.255637	-97.451675	Occurrence Download https://doi.org/10.15468/dl.3bxkun
Dioprosopa clavatus	Argentina	Córdoba	Oliva	-32.04144	-63.571919	Occurrence Download https://doi.org/10.15468/dl.3bxkun
Dioprosopa clavatus	United States of America	Texas	Fort Worth	32.89445	-97.351132	Occurrence Download https://doi.org/10.15468/dl.3bxkun
Dioprosopa clavatus	United States of America	Florida	South Venice	27.05389	-82.434933	Occurrence Download https://doi.org/10.15468/dl.3bxkun
Dioprosopa clavatus	United States of America	Florida	Bradenton	27.500539	-82.676327	Occurrence Download https://doi.org/10.15468/dl.3bxkun
Dioprosopa clavatus	United States of America	Florida	Saint Lucie County	27.247037	-80.64097	Occurrence Download https://doi.org/10.15468/dl.3bxkun
Dioprosopa clavatus	Brazil	Minas Gerais	Quartel de São João	-19.241386	-45.786431	Occurrence Download https://doi.org/10.15468/dl.3bxkun
Dioprosopa clavatus	United States of America	Texas	Bexar County	29.712342	-98.4039	Occurrence Download https://doi.org/10.15468/dl.3bxkun
Dioprosopa clavatus	United States of America	Texas	Blanco	30.103875	-98.425744	Occurrence Download https://doi.org/10.15468/dl.3bxkun
Dioprosopa clavatus	Argentina	Entre Ríos	Diamante	-32.122733	-60.632678	Occurrence Download https://doi.org/10.15468/dl.3bxkun
Dioprosopa clavatus	United States of America	Florida	Collier County	26.164635	-81.19353	Occurrence Download https://doi.org/10.15468/dl.3bxkun
Dioprosopa clavatus	Argentina	Córdoba	Bialet Massé	-31.315767	-64.498793	Occurrence Download https://doi.org/10.15468/dl.3bxkun
Dioprosopa clavatus	United States of America	Texas	Rosenberg	29.522937	-95.803545	Occurrence Download https://doi.org/10.15468/dl.3bxkun
Dioprosopa clavatus	Brazil	Distrito Federal	Park Way	-15.886936	-47.906966	Occurrence Download https://doi.org/10.15468/dl.3bxkun
Dioprosopa clavatus	United States of America	Texas	Bell County	30.977097	-97.58997	Occurrence Download https://doi.org/10.15468/dl.3bxkun
Dioprosopa clavatus	United States of America	Texas	San Antonio	29.362139	-98.589914	Occurrence Download https://doi.org/10.15468/dl.3bxkun
Dioprosopa clavatus	United States of America	Florida	Levy County	29.042112	-82.569319	Occurrence Download https://doi.org/10.15468/dl.3bxkun
Dioprosopa clavatus	United States of America	Texas	Austin	30.313663	-97.820587	Occurrence Download https://doi.org/10.15468/dl.3bxkun
Dioprosopa clavatus	Argentina	Córdoba	Oliva	-32.041433	-63.571906	Occurrence Download https://doi.org/10.15468/dl.3bxkun
Dioprosopa clavatus	Argentina	Córdoba	San Lorenzo	-31.670887	-65.02554	Occurrence Download https://doi.org/10.15468/dl.3bxkun
Dioprosopa clavatus	Argentina	Córdoba	Río Ceballos	-31.162492	-64.346931	Occurrence Download https://doi.org/10.15468/dl.3bxkun
Dioprosopa clavatus	Argentina	Córdoba	Pedanía Rosario	-31.254455	-64.603194	Occurrence Download https://doi.org/10.15468/dl.3bxkun
Dioprosopa clavatus	United States of America	Hawaii	Hawai‘i County	19.995195	-155.826	Occurrence Download https://doi.org/10.15468/dl.3bxkun

Dioprosopa clavatus	Nicaragua	Rivas	Monte Filis	11.452617	-86.1017	Occurrence Download https://doi.org/10.15468/dl.3bxkun
Dioprosopa clavatus	Argentina	Santa Fe	Santa Teresa	-33.441655	-60.794168	Occurrence Download https://doi.org/10.15468/dl.3bxkun
Dioprosopa clavatus	Argentina	Córdoba	Bialet Massé	-31.317922	-64.500196	Occurrence Download https://doi.org/10.15468/dl.3bxkun
Dioprosopa clavatus	Argentina	Santa Fe	Municipio de Tostado	-29.160193	-61.765402	Occurrence Download https://doi.org/10.15468/dl.3bxkun
Dioprosopa clavatus		Florida	Miami	25.785475	-80.187042	Occurrence Download https://doi.org/10.15468/dl.3bxkun
Dioprosopa clavatus	United States of America	Florida	Sarasota	27.333934	-82.575931	Occurrence Download https://doi.org/10.15468/dl.3bxkun
Dioprosopa clavatus	Argentina	Mendoza	Distrito Eugenio Bustos	-33.772279	-69.107244	Occurrence Download https://doi.org/10.15468/dl.3bxkun
Dioprosopa clavatus	Argentina	Mendoza	Distrito Gualtallary	-33.392163	-69.334071	Occurrence Download https://doi.org/10.15468/dl.3bxkun
Dioprosopa clavatus	Argentina	Santa Fe		-31.550905	-61.067578	Occurrence Download https://doi.org/10.15468/dl.3bxkun
Dioprosopa clavatus	Argentina	Santa Fe		-31.479773	-60.90563	Occurrence Download https://doi.org/10.15468/dl.3bxkun
Dioprosopa clavatus	United States of America	Florida	Marion County	28.976693	-82.223925	Occurrence Download https://doi.org/10.15468/dl.3bxkun
Dioprosopa clavatus	Argentina	Buenos Aires	Pilar	-34.412999	-58.916287	Occurrence Download https://doi.org/10.15468/dl.3bxkun
Dioprosopa clavatus	United States of America	Texas	Rio Grande City	26.385651	-98.832619	Occurrence Download https://doi.org/10.15468/dl.3bxkun
Dioprosopa clavatus	Peru	Lambayeque	Lambayeque	-6.707427	-79.906189	Occurrence Download https://doi.org/10.15468/dl.3bxkun
Dioprosopa clavatus	Argentina	Córdoba	Pedanía Río Cuarto	-33.210276	-64.299638	Occurrence Download https://doi.org/10.15468/dl.3bxkun
Dioprosopa clavatus	United States of America	Florida	Hillsborough County	28.044861	-82.17669	Occurrence Download https://doi.org/10.15468/dl.3bxkun
Dioprosopa clavatus	Peru	Lambayeque	Lambayeque	-6.707741	-79.904223	Occurrence Download https://doi.org/10.15468/dl.3bxkun
Dioprosopa clavatus	Mexico	Coahuila	Saltillo	25.334525	-100.992596	Occurrence Download https://doi.org/10.15468/dl.3bxkun
Dioprosopa clavatus	United States of America	Florida	Sarasota County	27.263354	-82.460213	Occurrence Download https://doi.org/10.15468/dl.3bxkun
Dioprosopa clavatus	United States of America	Texas	Lavaca County	29.455836	-97.197417	Occurrence Download https://doi.org/10.15468/dl.3bxkun
Dioprosopa clavatus	United States of America	Florida	Jacksonville	30.14386	-81.64908	Occurrence Download https://doi.org/10.15468/dl.3bxkun
Dioprosopa clavatus	United States of America	Florida	South Venice	27.040721	-82.421767	Occurrence Download https://doi.org/10.15468/dl.3bxkun
Dioprosopa clavatus	United States of America	Florida	Fairview	29.717235	-81.604377	Occurrence Download https://doi.org/10.15468/dl.3bxkun
Dioprosopa clavatus	United States of America	Florida	Wellington	26.632334	-80.300682	Occurrence Download https://doi.org/10.15468/dl.3bxkun
Dioprosopa clavatus	United States of America	Florida	Broward County	26.183382	-80.855613	Occurrence Download https://doi.org/10.15468/dl.3bxkun
Dioprosopa clavatus	United States of America	Texas	Rusk County	32.275256	-94.565565	Occurrence Download https://doi.org/10.15468/dl.3bxkun
Dioprosopa clavatus	United States of America	Texas	Alvin	29.44036	-95.225	Occurrence Download https://doi.org/10.15468/dl.3bxkun
Dioprosopa clavatus	United States of America	Texas	Weslaco	26.147692	-97.98871	Occurrence Download https://doi.org/10.15468/dl.3bxkun
Dioprosopa clavatus	United States of America	Hawaii	Hawaiian Ocean View	19.087231	-155.777061	Occurrence Download https://doi.org/10.15468/dl.3bxkun
Dioprosopa clavatus	Argentina	Santa Fe		-31.511261	-60.996259	Occurrence Download https://doi.org/10.15468/dl.3bxkun
Dioprosopa clavatus	Argentina	La Pampa	Municipio de General Acha	-37.368794	-64.599867	Occurrence Download https://doi.org/10.15468/dl.3bxkun
Dioprosopa clavatus	Mexico	Chihuahua	Meoqui	28.261424	-105.496974	Occurrence Download https://doi.org/10.15468/dl.3bxkun
Dioprosopa clavatus	Mexico	Chihuahua	Meoqui	28.263615	-105.492132	Occurrence Download https://doi.org/10.15468/dl.3bxkun
Dioprosopa clavatus	United States of America	Florida	Fort Peyton	29.821699	-81.347683	Occurrence Download https://doi.org/10.15468/dl.3bxkun
Dioprosopa clavatus	United States of America	Oklahoma	Washington	35.060683	-97.491049	Occurrence Download https://doi.org/10.15468/dl.3bxkun
Dioprosopa clavatus	United States of America	Florida	Yulee	30.627977	-81.584586	Occurrence Download https://doi.org/10.15468/dl.3bxkun
Dioprosopa clavatus	Mexico	Guerrero	Zihuatanejo de Azueta	17.649343	-101.519584	Occurrence Download https://doi.org/10.15468/dl.3bxkun
Dioprosopa clavatus	Mexico	Nuevo León	Juárez	25.637106	-100.164688	Occurrence Download https://doi.org/10.15468/dl.3bxkun
Dioprosopa clavatus	Mexico	Chihuahua	Gran Morelos (Los Cisneros)	28.289861	-105.438248	Occurrence Download https://doi.org/10.15468/dl.3bxkun

Dioprosopa clavatus	Argentina	Santa Fe	Municipio de Sauce Viejo	-31.762411	-60.831036	Occurrence Download https://doi.org/10.15468/dl.3bxkun
Dioprosopa clavatus	United States of America	Florida	North Port	27.088354	-82.235806	Occurrence Download https://doi.org/10.15468/dl.3bxkun
Dioprosopa clavatus	United States of America	Texas	Bryan	30.676636	-96.417542	Occurrence Download https://doi.org/10.15468/dl.3bxkun
Dioprosopa clavatus	United States of America	Texas	Bryan	30.676605	-96.417519	Occurrence Download https://doi.org/10.15468/dl.3bxkun
Dioprosopa clavatus	United States of America	Florida	Tampa	27.822599	-82.540613	Occurrence Download https://doi.org/10.15468/dl.3bxkun
Dioprosopa clavatus	United States of America	Florida	Orlando	28.409019	-81.315297	Occurrence Download https://doi.org/10.15468/dl.3bxkun
Dioprosopa clavatus	United States of America	Arizona	Chandler	33.23698	-111.769508	Occurrence Download https://doi.org/10.15468/dl.3bxkun
Dioprosopa clavatus	United States of America	Texas	Williamson County	30.684293	-97.64419	Occurrence Download https://doi.org/10.15468/dl.3bxkun
Dioprosopa clavatus	United States of America	Hawaii	Pahala	19.201919	-155.477753	Occurrence Download https://doi.org/10.15468/dl.3bxkun
Dioprosopa clavatus	United States of America	Louisiana	Lafayette	30.131614	-92.032824	Occurrence Download https://doi.org/10.15468/dl.3bxkun
Dioprosopa clavatus	United States of America	Arkansas	Franklin County	35.456899	-93.76159	Occurrence Download https://doi.org/10.15468/dl.3bxkun
Dioprosopa clavatus	United States of America	Texas	Pearland	29.581531	-95.41552	Occurrence Download https://doi.org/10.15468/dl.3bxkun
Dioprosopa clavatus	United States of America	Florida	Polk County	28.062719	-81.880052	Occurrence Download https://doi.org/10.15468/dl.3bxkun
Dioprosopa clavatus	United States of America	Oklahoma	Slaughterville	35.076694	-97.331266	Occurrence Download https://doi.org/10.15468/dl.3bxkun
Dioprosopa clavatus	United States of America	Florida	Yulee	30.62714	-81.583542	Occurrence Download https://doi.org/10.15468/dl.3bxkun
Dioprosopa clavatus	United States of America	Texas	Waco	31.482531	-97.282777	Occurrence Download https://doi.org/10.15468/dl.3bxkun
Dioprosopa clavatus	United States of America	Arkansas	Stone County	35.844565	-92.377552	Occurrence Download https://doi.org/10.15468/dl.3bxkun
Dioprosopa clavatus	United States of America	Florida	DeLand	29.093983	-81.349617	Occurrence Download https://doi.org/10.15468/dl.3bxkun
Dioprosopa clavatus	United States of America	Louisiana	Bossier City	32.458995	-93.649728	Occurrence Download https://doi.org/10.15468/dl.3bxkun
Dioprosopa clavatus	United States of America	Florida	Jacksonville	30.139922	-81.605713	Occurrence Download https://doi.org/10.15468/dl.3bxkun
Dioprosopa clavatus	United States of America	Florida	Blue Spring Landing	28.94635	-81.332996	Occurrence Download https://doi.org/10.15468/dl.3bxkun
Dioprosopa clavatus	United States of America	Maryland	Anne Arundel County	39.072786	-76.77583	Occurrence Download https://doi.org/10.15468/dl.3bxkun
Dioprosopa clavatus	United States of America	New York	Palenville	42.178906	-74.000501	Occurrence Download https://doi.org/10.15468/dl.3bxkun
Dioprosopa clavatus	United States of America	Florida	Polk County	28.295539	-82.002726	Occurrence Download https://doi.org/10.15468/dl.3bxkun
Dioprosopa clavatus	United States of America	Texas	Manvel	29.480286	-95.352674	Occurrence Download https://doi.org/10.15468/dl.3bxkun
Dioprosopa clavatus	United States of America	Texas	Baylor County	33.5524	-99.275132	Occurrence Download https://doi.org/10.15468/dl.3bxkun
Dioprosopa clavatus	United States of America	Nebraska	Lancaster County	40.665942	-96.608623	Occurrence Download https://doi.org/10.15468/dl.3bxkun
Dioprosopa clavatus	United States of America	Oklahoma	Norman	35.205494	-97.421834	Occurrence Download https://doi.org/10.15468/dl.3bxkun
Dioprosopa clavatus	Mexico	Durango	Gómez Palacio	25.598377	-103.454534	Occurrence Download https://doi.org/10.15468/dl.3bxkun
Dioprosopa clavatus	Mexico	Durango	Gómez Palacio	25.598462	-103.454625	Occurrence Download https://doi.org/10.15468/dl.3bxkun
Dioprosopa clavatus	United States of America	Texas	Bandera County	29.823536	-99.568212	Occurrence Download https://doi.org/10.15468/dl.3bxkun
Dioprosopa clavatus	United States of America	Tennessee	Tremont	35.640062	-83.689099	Occurrence Download https://doi.org/10.15468/dl.3bxkun
Dioprosopa clavatus	United States of America	Georgia	Jones County	32.942447	-83.419927	Occurrence Download https://doi.org/10.15468/dl.3bxkun
Dioprosopa clavatus	Peru	Lima	Papa León X III	-12.475535	-76.738086	Occurrence Download https://doi.org/10.15468/dl.3bxkun
Dioprosopa clavatus	United States of America	Florida	Deltona	28.877744	-81.259437	Occurrence Download https://doi.org/10.15468/dl.3bxkun
Dioprosopa clavatus	United States of America	Florida	Citrus County	28.749672	-82.509024	Occurrence Download https://doi.org/10.15468/dl.3bxkun
Dioprosopa clavatus	United States of America	Nebraska	Saunders County	41.103031	-96.445305	Occurrence Download https://doi.org/10.15468/dl.3bxkun
Dioprosopa clavatus	United States of America	Missouri	Polk County	37.587041	-93.242386	Occurrence Download https://doi.org/10.15468/dl.3bxkun
Dioprosopa clavatus	United States of America	Arkansas	Conway	35.046955	-92.496776	Occurrence Download https://doi.org/10.15468/dl.3bxkun

Dioprosopa clavatus	United States of America	Texas	Lubbock	33.502941	-101.851095	Occurrence Download https://doi.org/10.15468/dl.3bxkun
Dioprosopa clavatus	United States of America	Texas	Grayson County	33.819447	-96.859056	Occurrence Download https://doi.org/10.15468/dl.3bxkun
Dioprosopa clavatus	United States of America	Florida	Palm Beach County	26.795303	-80.244102	Occurrence Download https://doi.org/10.15468/dl.3bxkun
Dioprosopa clavatus	United States of America	Louisiana	Bossier City	32.457811	-93.649653	Occurrence Download https://doi.org/10.15468/dl.3bxkun
Dioprosopa clavatus	United States of America	Texas	Lubbock County	33.413035	-101.984304	Occurrence Download https://doi.org/10.15468/dl.3bxkun
Dioprosopa clavatus	United States of America	Texas	Grayson County	33.785117	-96.905008	Occurrence Download https://doi.org/10.15468/dl.3bxkun
Dioprosopa clavatus	United States of America	Texas	Brownsboro	32.29913	-95.614075	Occurrence Download https://doi.org/10.15468/dl.3bxkun
Dioprosopa clavatus	United States of America	Florida	Sarasota County	27.316455	-82.391602	Occurrence Download https://doi.org/10.15468/dl.3bxkun
Dioprosopa clavatus	United States of America	Nevada	Beatty	36.906338	-116.761841	Occurrence Download https://doi.org/10.15468/dl.3bxkun
Dioprosopa clavatus	Peru	Lima	Papa León X III	-12.475897	-76.738013	Occurrence Download https://doi.org/10.15468/dl.3bxkun
Dioprosopa clavatus	United States of America	Florida	Cumpressco	28.34002	-82.035774	Occurrence Download https://doi.org/10.15468/dl.3bxkun
Dioprosopa clavatus	United States of America	Texas	Benbrook	32.663846	-97.457645	Occurrence Download https://doi.org/10.15468/dl.3bxkun
Dioprosopa clavatus	United States of America	Mississippi	Hernando	34.817496	-89.983709	Occurrence Download https://doi.org/10.15468/dl.3bxkun
Dioprosopa clavatus	United States of America	Kansas	Johnson County	38.890364	-94.878321	Occurrence Download https://doi.org/10.15468/dl.3bxkun
Dioprosopa clavatus	United States of America	Kentucky	Glasgow	36.992097	-85.92012	Occurrence Download https://doi.org/10.15468/dl.3bxkun
Dioprosopa clavatus	United States of America	Kentucky	Louisville	38.3255	-85.56471	Occurrence Download https://doi.org/10.15468/dl.3bxkun
Dioprosopa clavatus	United States of America	California	San Diego County	32.973265	-116.58384	Occurrence Download https://doi.org/10.15468/dl.3bxkun
Dioprosopa clavatus	United States of America	Nebraska	Kearney	40.716244	-99.078382	Occurrence Download https://doi.org/10.15468/dl.3bxkun
Dioprosopa clavatus	United States of America	Georgia	Chatham County	31.954253	-81.046469	Occurrence Download https://doi.org/10.15468/dl.3bxkun
Dioprosopa clavatus	United States of America	Texas	Grayson County	33.785131	-96.905089	Occurrence Download https://doi.org/10.15468/dl.3bxkun
Dioprosopa clavatus	United States of America	Florida	Polk County	28.055308	-81.884661	Occurrence Download https://doi.org/10.15468/dl.3bxkun
Dioprosopa clavatus	United States of America	Nebraska	Kearney	40.716303	-99.078532	Occurrence Download https://doi.org/10.15468/dl.3bxkun
Dioprosopa clavatus	Mexico	Chihuahua	Meoqui	28.263024	-105.493858	Occurrence Download https://doi.org/10.15468/dl.3bxkun
Dioprosopa clavatus	Mexico	Puebla	San Francisco Totimehuacán	18.9942	-98.169085	Occurrence Download https://doi.org/10.15468/dl.3bxkun
Dioprosopa clavatus	United States of America	California	Butte County	39.489754	-121.447538	Occurrence Download https://doi.org/10.15468/dl.3bxkun
Dioprosopa clavatus	United States of America	Missouri	Boone County	38.833476	-92.338995	Occurrence Download https://doi.org/10.15468/dl.3bxkun
Dioprosopa clavatus	Mexico	Querétaro	Corregidora	20.521878	-100.388051	Occurrence Download https://doi.org/10.15468/dl.3bxkun
Dioprosopa clavatus	United States of America	Kansas	Louisburg	38.609343	-94.677761	Occurrence Download https://doi.org/10.15468/dl.3bxkun
Dioprosopa clavatus	United States of America	Kansas	Olathe	38.891062	-94.863678	Occurrence Download https://doi.org/10.15468/dl.3bxkun
Dioprosopa clavatus	Mexico	Chihuahua	Meoqui	28.259802	-105.500105	Occurrence Download https://doi.org/10.15468/dl.3bxkun
Dioprosopa clavatus	Brazil	Paraíba	Juarez Távora	-7.123889	-35.528333	Occurrence Download https://doi.org/10.15468/dl.3bxkun
Dioprosopa clavatus	United States of America	Florida	Florida Ridge	27.575571	-80.411941	Occurrence Download https://doi.org/10.15468/dl.3bxkun
Dioprosopa clavatus	United States of America	California	Riverside	33.90809	-117.394472	Occurrence Download https://doi.org/10.15468/dl.3bxkun
Dioprosopa clavatus	United States of America	Georgia	Jones County	32.942752	-83.419558	Occurrence Download https://doi.org/10.15468/dl.3bxkun
Dioprosopa clavatus	Mexico	Michoacán	Tenencia Morelos	19.670912	-101.233131	Occurrence Download https://doi.org/10.15468/dl.3bxkun
Dioprosopa clavatus	Mexico	Guanajuato	Acámbaro	19.95585	-100.700755	Occurrence Download https://doi.org/10.15468/dl.3bxkun
Dioprosopa clavatus	United States of America	Kentucky	Highland Heights	39.033558	-84.462685	Occurrence Download https://doi.org/10.15468/dl.3bxkun
Dioprosopa clavatus	United States of America	California	Merced County	37.388286	-120.400635	Occurrence Download https://doi.org/10.15468/dl.3bxkun
Dioprosopa clavatus	United States of America	Arizona	Sierra Vista	31.489848	-110.185068	Occurrence Download https://doi.org/10.15468/dl.3bxkun

Dioprosopa clavatus	Mexico	Chihuahua	Meoqui	28.262904	-105.49428	Occurrence Download https://doi.org/10.15468/dl.3bxkun
Dioprosopa clavatus	United States of America	California	Merced County	37.393775	-120.441558	Occurrence Download https://doi.org/10.15468/dl.3bxkun
Dioprosopa clavatus	United States of America	North Carolina	Elizabeth City	36.295258	-76.216817	Occurrence Download https://doi.org/10.15468/dl.3bxkun
Dioprosopa clavatus	United States of America	Mississippi	Monroe County	33.922722	-88.516086	Occurrence Download https://doi.org/10.15468/dl.3bxkun
Dioprosopa clavatus	United States of America	Georgia	Grady County	30.90233	-84.228588	Occurrence Download https://doi.org/10.15468/dl.3bxkun
Dioprosopa clavatus	United States of America	Georgia	Euchee Creek	33.465894	-82.230339	Occurrence Download https://doi.org/10.15468/dl.3bxkun
Dioprosopa clavatus	United States of America	Florida	Rockledge	28.313935	-80.711417	Occurrence Download https://doi.org/10.15468/dl.3bxkun
Dioprosopa clavatus	United States of America	Louisiana	Jackson	30.83616	-91.2107	Occurrence Download https://doi.org/10.15468/dl.3bxkun
Dioprosopa clavatus	United States of America	Oklahoma	Cole	35.020021	-97.564211	Occurrence Download https://doi.org/10.15468/dl.3bxkun
Dioprosopa clavatus	United States of America	North Carolina	Robeson County	34.752818	-79.326946	Occurrence Download https://doi.org/10.15468/dl.3bxkun
Dioprosopa clavatus	United States of America	Arkansas	Caraway	35.759526	-90.326966	Occurrence Download https://doi.org/10.15468/dl.3bxkun
Dioprosopa clavatus	United States of America	Oklahoma	Norman	35.190338	-97.271873	Occurrence Download https://doi.org/10.15468/dl.3bxkun
Dioprosopa clavatus	United States of America	Oklahoma	Norman	35.219254	-97.402246	Occurrence Download https://doi.org/10.15468/dl.3bxkun
Dioprosopa clavatus	United States of America	New Jersey	Hamilton Township	39.546465	-74.751535	Occurrence Download https://doi.org/10.15468/dl.3bxkun
Dioprosopa clavatus	United States of America	California	Merced County	37.207402	-120.522593	Occurrence Download https://doi.org/10.15468/dl.3bxkun
Dioprosopa clavatus	United States of America	North Carolina	Pitt County	35.405185	-77.207501	Occurrence Download https://doi.org/10.15468/dl.3bxkun
Dioprosopa clavatus	Mexico	Jalisco	El Terrero	20.760133	-103.262748	Occurrence Download https://doi.org/10.15468/dl.3bxkun
Dioprosopa clavatus	United States of America	California	Santa Barbara County	34.51779	-119.620764	Occurrence Download https://doi.org/10.15468/dl.3bxkun
Dioprosopa clavatus	United States of America	Minnesota	Northfield	44.459231	-93.181973	Occurrence Download https://doi.org/10.15468/dl.3bxkun
Dioprosopa clavatus	United States of America	California	Solano County	38.50871	-122.050841	Occurrence Download https://doi.org/10.15468/dl.3bxkun
Dioprosopa clavatus	United States of America	Georgia	Jones County	32.942376	-83.420432	Occurrence Download https://doi.org/10.15468/dl.3bxkun
Dioprosopa clavatus	United States of America	Georgia	Gray	33.000754	-83.529359	Occurrence Download https://doi.org/10.15468/dl.3bxkun
Dioprosopa clavatus	Mexico	Sinaloa	Chilillos	22.997783	-105.848394	Occurrence Download https://doi.org/10.15468/dl.3bxkun
Dioprosopa clavatus	United States of America	California	Merced County	37.169552	-120.616992	Occurrence Download https://doi.org/10.15468/dl.3bxkun
Dioprosopa clavatus	United States of America	Oklahoma	Newcastle	35.140295	-97.557451	Occurrence Download https://doi.org/10.15468/dl.3bxkun
Dioprosopa clavatus	United States of America	Florida	Osceola County	28.042232	-81.039202	Occurrence Download https://doi.org/10.15468/dl.3bxkun
Dioprosopa clavatus	United States of America	Texas	Abilene	32.440363	-99.692428	Occurrence Download https://doi.org/10.15468/dl.3bxkun
Dioprosopa clavatus	United States of America	Florida	Sarasota County	27.19519	-82.441861	Occurrence Download https://doi.org/10.15468/dl.3bxkun
Dioprosopa clavatus	Mexico	Chihuahua	Meoqui	28.260687	-105.484857	Occurrence Download https://doi.org/10.15468/dl.3bxkun
Dioprosopa clavatus	United States of America	Texas	Flower Mound	33.023857	-97.145695	Occurrence Download https://doi.org/10.15468/dl.3bxkun
Dioprosopa clavatus	Mexico	Yucatán	Dzityá	21.045242	-89.662188	Occurrence Download https://doi.org/10.15468/dl.3bxkun
Dioprosopa clavatus	Mexico	Yucatán	Dzityá	21.045145	-89.662112	Occurrence Download https://doi.org/10.15468/dl.3bxkun
Dioprosopa clavatus	Mexico	Chihuahua	Colonia Francisco Portillo (Los Jáquez)	28.268887	-105.462844	Occurrence Download https://doi.org/10.15468/dl.3bxkun
Dioprosopa clavatus	United States of America	Virginia	Loudoun Valley Estates	38.981903	-77.512588	Occurrence Download https://doi.org/10.15468/dl.3bxkun
Dioprosopa clavatus	United States of America	California	Coachella	33.65786	-116.19372	Occurrence Download https://doi.org/10.15468/dl.3bxkun
Dioprosopa clavatus	United States of America	Florida	Martin County	26.972131	-80.564287	Occurrence Download https://doi.org/10.15468/dl.3bxkun
Dioprosopa clavatus	United States of America	Texas	Rosenberg	29.524084	-95.801004	Occurrence Download https://doi.org/10.15468/dl.3bxkun
Dioprosopa clavatus	United States of America	Florida	Groveland	28.481152	-81.876511	Occurrence Download https://doi.org/10.15468/dl.3bxkun
Dioprosopa clavatus	United States of America	Texas	Denton	33.1744	-97.125328	Occurrence Download https://doi.org/10.15468/dl.3bxkun

Dioprosopa clavatus	United States of America	Oklahoma	Moore	35.300887	-97.423891	Occurrence Download https://doi.org/10.15468/dl.3bxkun
Dioprosopa clavatus	United States of America	Florida	Osprey	27.175612	-82.460492	Occurrence Download https://doi.org/10.15468/dl.3bxkun
Dioprosopa clavatus	Mexico	Yucatán	Xmatkuil	20.869385	-89.624101	Occurrence Download https://doi.org/10.15468/dl.3bxkun
Dioprosopa clavatus	United States of America	Texas	Rosenberg	29.524049	-95.796253	Occurrence Download https://doi.org/10.15468/dl.3bxkun
Dioprosopa clavatus	United States of America	Texas	College Station	30.547738	-96.298858	Occurrence Download https://doi.org/10.15468/dl.3bxkun
Dioprosopa clavatus	United States of America	Missouri	Joplin	37.032992	-94.541528	Occurrence Download https://doi.org/10.15468/dl.3bxkun
Dioprosopa clavatus	United States of America	California	Yuba County	39.314358	-121.332662	Occurrence Download https://doi.org/10.15468/dl.3bxkun
Dioprosopa clavatus	United States of America	Kansas	Cherokee County	37.181624	-94.649589	Occurrence Download https://doi.org/10.15468/dl.3bxkun
Dioprosopa clavatus	United States of America	Texas	Milam County	30.789488	-96.951545	Occurrence Download https://doi.org/10.15468/dl.3bxkun
Dioprosopa clavatus	United States of America	Florida	Ethel	28.804053	-81.452452	Occurrence Download https://doi.org/10.15468/dl.3bxkun
Dioprosopa clavatus	United States of America	South Carolina	James Island	32.742662	-79.948608	Occurrence Download https://doi.org/10.15468/dl.3bxkun
Dioprosopa clavatus	Mexico	Chihuahua	Meoqui	28.262555	-105.494739	Occurrence Download https://doi.org/10.15468/dl.3bxkun
Dioprosopa clavatus		Texas	Fulton	28.07395	-97.041476	Occurrence Download https://doi.org/10.15468/dl.3bxkun
Dioprosopa clavatus	United States of America	Florida	Sarasota County	27.192967	-82.189758	Occurrence Download https://doi.org/10.15468/dl.3bxkun
Dioprosopa clavatus	United States of America	Texas	Mission	26.179978	-98.366804	Occurrence Download https://doi.org/10.15468/dl.3bxkun
Dioprosopa clavatus	United States of America	Texas	Mission	26.179918	-98.366051	Occurrence Download https://doi.org/10.15468/dl.3bxkun
Dioprosopa clavatus	United States of America	Texas	Rosenberg	29.5268	-95.801087	Occurrence Download https://doi.org/10.15468/dl.3bxkun
Dioprosopa clavatus	United States of America	South Carolina	Charleston	32.724593	-79.947355	Occurrence Download https://doi.org/10.15468/dl.3bxkun
Dioprosopa clavatus	United States of America	Texas	Frio County	28.933268	-99.064273	Occurrence Download https://doi.org/10.15468/dl.3bxkun
Dioprosopa clavatus		Texas	Fulton	28.073935	-97.041484	Occurrence Download https://doi.org/10.15468/dl.3bxkun
Dioprosopa clavatus	United States of America	Texas	Limestone County	31.464768	-96.365449	Occurrence Download https://doi.org/10.15468/dl.3bxkun
Dioprosopa clavatus	Brazil	Rio Grande do Sul	Santa Maria	-29.724664	-53.712769	Occurrence Download https://doi.org/10.15468/dl.3bxkun
Dioprosopa clavatus	United States of America	Florida	DeSoto County	27.214527	-81.900276	Occurrence Download https://doi.org/10.15468/dl.3bxkun
Dioprosopa clavatus	United States of America	South Carolina	Hilton Head Island	32.127365	-80.780029	Occurrence Download https://doi.org/10.15468/dl.3bxkun
Dioprosopa clavatus	United States of America	Florida	Tallahassee	30.425944	-84.316003	Occurrence Download https://doi.org/10.15468/dl.3bxkun
Dioprosopa clavatus	United States of America	Texas	Mission	26.179118	-98.36614	Occurrence Download https://doi.org/10.15468/dl.3bxkun
Dioprosopa clavatus	United States of America	Florida	Fort De Soto	27.614539	-82.736669	Occurrence Download https://doi.org/10.15468/dl.3bxkun
Dioprosopa clavatus	Argentina	Corrientes	Municipio de Ituzaingó	-27.54952	-56.643864	Occurrence Download https://doi.org/10.15468/dl.3bxkun
Dioprosopa clavatus	Peru	Lima	Surco	-11.887959	-76.442514	Occurrence Download https://doi.org/10.15468/dl.3bxkun
Dioprosopa clavatus	Mexico	Tamaulipas	Hidalgo County	26.1789	-98.386336	Occurrence Download https://doi.org/10.15468/dl.3bxkun
Dioprosopa clavatus	Mexico	Tamaulipas	Hidalgo County	26.17885	-98.386458	Occurrence Download https://doi.org/10.15468/dl.3bxkun
Dioprosopa clavatus	United States of America	Texas	Mission	26.179169	-98.366189	Occurrence Download https://doi.org/10.15468/dl.3bxkun
Dioprosopa clavatus	United States of America	Florida	Polk County	27.969127	-81.646834	Occurrence Download https://doi.org/10.15468/dl.3bxkun
Dioprosopa clavatus	United States of America	Florida	Manatee County	27.453554	-82.139246	Occurrence Download https://doi.org/10.15468/dl.3bxkun
Dioprosopa clavatus	United States of America	Louisiana	St. George	30.356691	-91.095027	Occurrence Download https://doi.org/10.15468/dl.3bxkun
Dioprosopa clavatus	United States of America	Texas	Waco	31.590549	-97.249212	Occurrence Download https://doi.org/10.15468/dl.3bxkun
Dioprosopa clavatus	United States of America	Texas	Montgomery County	30.143313	-95.775312	Occurrence Download https://doi.org/10.15468/dl.3bxkun
Dioprosopa clavatus	Mexico	Tamaulipas	Hidalgo County	26.178751	-98.386398	Occurrence Download https://doi.org/10.15468/dl.3bxkun
Dioprosopa clavatus	United States of America	California	Willis Palms	33.824943	-116.317426	Occurrence Download https://doi.org/10.15468/dl.3bxkun

Dioprosopa clavatus	United States of America	Texas	Indigo Lake Estates	30.166675	-95.76308	Occurrence Download https://doi.org/10.15468/dl.3bxkun
Dioprosopa clavatus	United States of America	Texas	Mission	26.179873	-98.366466	Occurrence Download https://doi.org/10.15468/dl.3bxkun
Dioprosopa clavatus	United States of America	Louisiana	Henderson	30.343342	-91.71997	Occurrence Download https://doi.org/10.15468/dl.3bxkun
Dioprosopa clavatus	United States of America	Texas	Brownsville	25.996487	-97.568528	Occurrence Download https://doi.org/10.15468/dl.3bxkun
Dioprosopa clavatus	United States of America	Texas	Maypearl	32.311782	-97.01597	Occurrence Download https://doi.org/10.15468/dl.3bxkun
Dioprosopa clavatus	United States of America	Florida	Laurel	27.17403	-82.461852	Occurrence Download https://doi.org/10.15468/dl.3bxkun
Dioprosopa clavatus	United States of America	Florida	Sarasota County	27.12304	-82.352203	Occurrence Download https://doi.org/10.15468/dl.3bxkun
Dioprosopa clavatus	United States of America	Texas	Mission	26.179467	-98.366316	Occurrence Download https://doi.org/10.15468/dl.3bxkun
Dioprosopa clavatus	United States of America	Texas	Garden Ridge	29.641608	-98.321262	Occurrence Download https://doi.org/10.15468/dl.3bxkun
Dioprosopa clavatus	United States of America	Texas	Comal County	29.890849	-98.295269	Occurrence Download https://doi.org/10.15468/dl.3bxkun
Dioprosopa clavatus	United States of America	Texas	Aransas County	28.303264	-96.804376	Occurrence Download https://doi.org/10.15468/dl.3bxkun
Dioprosopa clavatus	United States of America	Texas	Weslaco	26.127121	-97.957975	Occurrence Download https://doi.org/10.15468/dl.3bxkun
Dioprosopa clavatus	United States of America	Texas	Weslaco	26.126889	-97.958017	Occurrence Download https://doi.org/10.15468/dl.3bxkun
Dioprosopa clavatus	Argentina	Córdoba	Camilo Aldao	-33.121053	-62.094635	Occurrence Download https://doi.org/10.15468/dl.3bxkun
Dioprosopa clavatus	United States of America	Texas	Aransas County	28.303236	-96.804408	Occurrence Download https://doi.org/10.15468/dl.3bxkun
Dioprosopa clavatus	United States of America	Texas	Weslaco	26.126832	-97.957975	Occurrence Download https://doi.org/10.15468/dl.3bxkun
Dioprosopa clavatus	United States of America	Texas	Weslaco	26.127121	-97.958082	Occurrence Download https://doi.org/10.15468/dl.3bxkun
Dioprosopa clavatus	United States of America	Texas	Weslaco	26.127101	-97.957953	Occurrence Download https://doi.org/10.15468/dl.3bxkun
Dioprosopa clavatus	United States of America	Texas	Weslaco	26.127121	-97.957996	Occurrence Download https://doi.org/10.15468/dl.3bxkun
Dioprosopa clavatus	United States of America	Texas	Weslaco	26.127082	-97.95791	Occurrence Download https://doi.org/10.15468/dl.3bxkun
Dioprosopa clavatus	United States of America	Florida	Tallahassee	30.407661	-84.309899	Occurrence Download https://doi.org/10.15468/dl.3bxkun
Dioprosopa clavatus	United States of America	Texas	Jim Wells County	28.040845	-97.86631	Occurrence Download https://doi.org/10.15468/dl.3bxkun
Dioprosopa clavatus	United States of America	Texas	Garden Ridge	29.641348	-98.321359	Occurrence Download https://doi.org/10.15468/dl.3bxkun
Dioprosopa clavatus	United States of America	Texas	Denton County	33.136161	-97.151764	Occurrence Download https://doi.org/10.15468/dl.3bxkun
Dioprosopa clavatus	Mexico	Chihuahua	Meoqui	28.263067	-105.493846	Occurrence Download https://doi.org/10.15468/dl.3bxkun
Dioprosopa clavatus	Mexico	Chihuahua	Meoqui	28.263086	-105.493731	Occurrence Download https://doi.org/10.15468/dl.3bxkun
Dioprosopa clavatus	Mexico	Chihuahua	Meoqui	28.263062	-105.494016	Occurrence Download https://doi.org/10.15468/dl.3bxkun
Dioprosopa clavatus	Argentina	Córdoba	Pedanía Los Reartes	-31.989104	-64.730741	Occurrence Download https://doi.org/10.15468/dl.3bxkun
Dioprosopa clavatus	United States of America	Texas	Fort Bend County	29.660264	-95.715556	Occurrence Download https://doi.org/10.15468/dl.3bxkun
Dioprosopa clavatus	United States of America	Texas	Campbellton	28.76267	-98.301255	Occurrence Download https://doi.org/10.15468/dl.3bxkun
Dioprosopa clavatus	United States of America	Texas	Jackson County	28.809989	-96.573915	Occurrence Download https://doi.org/10.15468/dl.3bxkun
Dioprosopa clavatus	United States of America	Texas	Jackson County	28.913888	-96.428358	Occurrence Download https://doi.org/10.15468/dl.3bxkun
Dioprosopa clavatus	United States of America	Texas	Jackson County	28.894036	-96.402124	Occurrence Download https://doi.org/10.15468/dl.3bxkun
Dioprosopa clavatus	Argentina	Córdoba	Pedanía San Roque	-31.45512	-64.542572	Occurrence Download https://doi.org/10.15468/dl.3bxkun
Dioprosopa clavatus	Argentina	Córdoba	Pedanía San Roque	-31.455167	-64.542347	Occurrence Download https://doi.org/10.15468/dl.3bxkun
Dioprosopa clavatus	Argentina	Córdoba	Intiyaco	-31.961366	-64.71844	Occurrence Download https://doi.org/10.15468/dl.3bxkun
Dioprosopa clavatus	United States of America	Tamaulipas	Mission	26.191333	-98.374681	Occurrence Download https://doi.org/10.15468/dl.3bxkun
Dioprosopa clavatus	Mexico	Tamaulipas	Hidalgo County	26.174831	-98.383544	Occurrence Download https://doi.org/10.15468/dl.3bxkun
Dioprosopa clavatus	United States of America	Texas	Houston	29.939076	-95.170648	Occurrence Download https://doi.org/10.15468/dl.3bxkun

Dioprosopa clavatus	Brazil	Minas Gerais	Santa Luzia	-19.812854	-43.865624	Occurrence Download https://doi.org/10.15468/dl.3bxkun
Dioprosopa clavatus	Mexico	Sinaloa	Concordia	23.479297	-106.202967	Occurrence Download https://doi.org/10.15468/dl.3bxkun
Dioprosopa clavatus	Chile	Coquimbo	Salamanca	-31.775636	-70.972393	Occurrence Download https://doi.org/10.15468/dl.3bxkun
Dioprosopa clavatus	United States of America	Texas	Hidalgo County	26.125986	-97.952239	Occurrence Download https://doi.org/10.15468/dl.3bxkun
Dioprosopa clavatus	Argentina	Córdoba	Pedanía Rosario	-31.231739	-64.501602	Occurrence Download https://doi.org/10.15468/dl.3bxkun
Dioprosopa clavatus	United States of America	Mississippi	Jackson County	30.369377	-88.767837	Occurrence Download https://doi.org/10.15468/dl.3bxkun
Dioprosopa clavatus	Brazil	São Paulo	Avaré	-23.140388	-48.899477	Occurrence Download https://doi.org/10.15468/dl.3bxkun
Dioprosopa clavatus	United States of America	Texas	Montgomery County	30.146686	-95.168638	Occurrence Download https://doi.org/10.15468/dl.3bxkun
Dioprosopa clavatus	United States of America	Hawaii	Kihei	20.795476	-156.485331	Occurrence Download https://doi.org/10.15468/dl.3bxkun
Dioprosopa clavatus	Mexico	Veracruz	Xalapa	19.527096	-96.886214	Occurrence Download https://doi.org/10.15468/dl.3bxkun
Dioprosopa clavatus	Argentina	Córdoba	Pedanía Santa Rosa	-32.060452	-64.64442	Occurrence Download https://doi.org/10.15468/dl.3bxkun
Dioprosopa clavatus	United States of America	Texas	Mission	26.176461	-98.366194	Occurrence Download https://doi.org/10.15468/dl.3bxkun
Dioprosopa clavatus	United States of America	Texas	Progreso Lakes	26.063229	-97.965919	Occurrence Download https://doi.org/10.15468/dl.3bxkun
Dioprosopa clavatus	Costa Rica	Heredia	San Juan	10.0248	-84.1436	Occurrence Download https://doi.org/10.15468/dl.3bxkun
Dioprosopa clavatus	Argentina	Córdoba	Los Chañares	-31.715403	-64.443722	Occurrence Download https://doi.org/10.15468/dl.3bxkun
Dioprosopa clavatus	Argentina	Mendoza	Papagayos	-32.812008	-68.906419	Occurrence Download https://doi.org/10.15468/dl.3bxkun
Dioprosopa clavatus	Argentina	Buenos Aires	Rincón de Milberg	-34.391092	-58.622467	Occurrence Download https://doi.org/10.15468/dl.3bxkun
Dioprosopa clavatus	United States of America	Hawaii	Mākaha Valley	21.499242	-158.199925	Occurrence Download https://doi.org/10.15468/dl.3bxkun
Dioprosopa clavatus	United States of America	Florida	West Melbourne	28.100674	-80.714891	Occurrence Download https://doi.org/10.15468/dl.3bxkun
Dioprosopa clavatus	Argentina	Buenos Aires	González Moreno	-35.568539	-63.379949	Occurrence Download https://doi.org/10.15468/dl.3bxkun
Dioprosopa clavatus	United States of America	Florida	Charlotte Park	26.897009	-82.05465	Occurrence Download https://doi.org/10.15468/dl.3bxkun
Dioprosopa clavatus	Argentina	Entre Ríos	Rocamora	-32.348395	-58.956807	Occurrence Download https://doi.org/10.15468/dl.3bxkun
Dioprosopa clavatus	United States of America	Florida	Homestead	25.520675	-80.476417	Occurrence Download https://doi.org/10.15468/dl.3bxkun
Dioprosopa clavatus	Peru	Lima	Jita	-12.970758	-76.150728	Occurrence Download https://doi.org/10.15468/dl.3bxkun
Dioprosopa clavatus	United States of America	Arizona	Santa Cruz County	31.562217	-111.046602	Occurrence Download https://doi.org/10.15468/dl.3bxkun
Dioprosopa clavatus	Argentina	Buenos Aires	Hudson	-34.754868	-58.124617	Occurrence Download https://doi.org/10.15468/dl.3bxkun
Dioprosopa clavatus	Argentina	Mendoza	Distrito Las Barrancas	-33.078681	-68.657766	Occurrence Download https://doi.org/10.15468/dl.3bxkun
Dioprosopa clavatus	Argentina	Mendoza	Distrito Las Barrancas	-33.056629	-68.761214	Occurrence Download https://doi.org/10.15468/dl.3bxkun
Dioprosopa clavatus	Argentina	Mendoza	Distrito Las Barrancas	-33.061995	-68.741454	Occurrence Download https://doi.org/10.15468/dl.3bxkun
Dioprosopa clavatus	Argentina	Mendoza	Distrito Las Barrancas	-33.077756	-68.635137	Occurrence Download https://doi.org/10.15468/dl.3bxkun
Dioprosopa clavatus	Argentina	Mendoza	Distrito Las Barrancas	-33.087817	-68.652262	Occurrence Download https://doi.org/10.15468/dl.3bxkun
Dioprosopa clavatus	Peru	Lambayeque	Pueblo Nuevo	-6.643573	-79.799798	Occurrence Download https://doi.org/10.15468/dl.3bxkun
Dioprosopa clavatus	United States of America	Texas	Anderson County	31.97491	-95.882887	Occurrence Download https://doi.org/10.15468/dl.3bxkun
Dioprosopa clavatus	United States of America	Texas	Corpus Christi	27.648981	-97.393552	Occurrence Download https://doi.org/10.15468/dl.3bxkun
Dioprosopa clavatus	United States of America	Florida	Brevard County	28.094061	-80.750676	Occurrence Download https://doi.org/10.15468/dl.3bxkun
Dioprosopa clavatus	United States of America	Texas	Waco	31.607172	-97.304081	Occurrence Download https://doi.org/10.15468/dl.3bxkun
Dioprosopa clavatus	United States of America	Texas	Vilas	30.837506	-97.304986	Occurrence Download https://doi.org/10.15468/dl.3bxkun
Dioprosopa clavatus	United States of America	Texas	Vilas	30.8382	-97.304864	Occurrence Download https://doi.org/10.15468/dl.3bxkun
Dioprosopa clavatus	United States of America	Florida	Miami-Dade County	25.393351	-80.689415	Occurrence Download https://doi.org/10.15468/dl.3bxkun

Dioprosopa clavatus	United States of America	Texas	Wimberley	29.999745	-98.101137	Occurrence Download	https://doi.org/10.15468/dl.3bxkun
Dioprosopa clavatus	United States of America	Florida	Sarasota County	27.249121	-82.474501	Occurrence Download	https://doi.org/10.15468/dl.3bxkun
Dioprosopa clavatus	United States of America	Arkansas	Washington County	35.782107	-94.250719	Occurrence Download	https://doi.org/10.15468/dl.3bxkun
Dioprosopa clavatus	Argentina	Santa Fe	San Jerónimo Norte	-31.550886	-61.067805	Occurrence Download	https://doi.org/10.15468/dl.3bxkun
Dioprosopa clavatus	Argentina	Córdoba	Cabalango	-31.395587	-64.551869	Occurrence Download	https://doi.org/10.15468/dl.3bxkun
Dioprosopa clavatus	United States of America	Hawaii	Olowalu	20.820801	-156.612813	Occurrence Download	https://doi.org/10.15468/dl.3bxkun
Dioprosopa clavatus	Brazil	Rio Grande do Sul	Silveira Martins	-29.696244	-53.541931	Occurrence Download	https://doi.org/10.15468/dl.3bxkun
Dioprosopa clavatus	United States of America	Florida	Miccosukee Indian Village	25.758568	-80.766479	Occurrence Download	https://doi.org/10.15468/dl.3bxkun
Dioprosopa clavatus	United States of America	Texas	Pearsall	28.932794	-99.096622	Occurrence Download	https://doi.org/10.15468/dl.3bxkun
Dioprosopa clavatus	United States of America	Texas	Comanche County	32.034369	-98.620766	Occurrence Download	https://doi.org/10.15468/dl.3bxkun
Dioprosopa clavatus	United States of America	Florida	Hillsborough County	28.044949	-82.176724	Occurrence Download	https://doi.org/10.15468/dl.3bxkun
Dioprosopa clavatus	United States of America	Texas	Georgetown	30.686177	-97.646703	Occurrence Download	https://doi.org/10.15468/dl.3bxkun
Dioprosopa clavatus	United States of America	Texas	Vilas	30.837492	-97.304987	Occurrence Download	https://doi.org/10.15468/dl.3bxkun
Dioprosopa clavatus	United States of America	Texas	Harlingen	26.158642	-97.701894	Occurrence Download	https://doi.org/10.15468/dl.3bxkun
Dioprosopa clavatus	United States of America	Florida	Orange County	28.454695	-81.079346	Occurrence Download	https://doi.org/10.15468/dl.3bxkun
Dioprosopa clavatus	United States of America	Hawaii	Hawaiʻi County	19.921207	-155.871198	Occurrence Download	https://doi.org/10.15468/dl.3bxkun
Dioprosopa clavatus	Belize	Orange Walk		17.761141	-88.63176	Occurrence Download	https://doi.org/10.15468/dl.3bxkun
Dioprosopa clavatus	United States of America	Arizona	Pima County	32.232301	-110.625004	Occurrence Download	https://doi.org/10.15468/dl.3bxkun
Dioprosopa clavatus	United States of America	Florida	Jacksonville	30.143846	-81.649095	Occurrence Download	https://doi.org/10.15468/dl.3bxkun
Dioprosopa clavatus	United States of America	Ohio	Lake County	41.659243	-81.133825	Occurrence Download	https://doi.org/10.15468/dl.3bxkun
Dioprosopa clavatus	United States of America	Texas	Fort Bend County	29.641014	-95.952431	Occurrence Download	https://doi.org/10.15468/dl.3bxkun
Dioprosopa clavatus	United States of America	Texas	Baytown	29.825354	-94.828675	Occurrence Download	https://doi.org/10.15468/dl.3bxkun
Dioprosopa clavatus	United States of America	Florida	Navarre	30.404662	-86.887428	Occurrence Download	https://doi.org/10.15468/dl.3bxkun
Dioprosopa clavatus	United States of America	Arizona	Cornville	34.761708	-111.889378	Occurrence Download	https://doi.org/10.15468/dl.3bxkun
Dioprosopa clavatus	Mexico	Guanajuato	Acámbaro	20.034718	-100.719858	Occurrence Download	https://doi.org/10.15468/dl.3bxkun
Dioprosopa clavatus	Mexico	Oaxaca	Vista Hermosa	16.534172	-98.175095	Occurrence Download	https://doi.org/10.15468/dl.3bxkun
Dioprosopa clavatus	United States of America	Florida	Levy County	29.042018	-82.569533	Occurrence Download	https://doi.org/10.15468/dl.3bxkun
Dioprosopa clavatus	United States of America	Mississippi	Gulfport	30.461092	-89.021077	Occurrence Download	https://doi.org/10.15468/dl.3bxkun
Dioprosopa clavatus	United States of America	Texas	Erath County	32.164443	-98.286442	Occurrence Download	https://doi.org/10.15468/dl.3bxkun
Dioprosopa clavatus	United States of America	Texas	Flower Mound	33.043311	-97.179476	Occurrence Download	https://doi.org/10.15468/dl.3bxkun
Dioprosopa clavatus	United States of America	Florida	Miami	25.615255	-80.306524	Occurrence Download	https://doi.org/10.15468/dl.3bxkun
Dioprosopa clavatus	United States of America	California	Fallbrook	33.310876	-117.288041	Occurrence Download	https://doi.org/10.15468/dl.3bxkun
Dioprosopa clavatus	United States of America	Texas	Dripping Springs	30.204035	-98.08005	Occurrence Download	https://doi.org/10.15468/dl.3bxkun
Dioprosopa clavatus	United States of America	Florida	DeLand	29.093815	-81.349531	Occurrence Download	https://doi.org/10.15468/dl.3bxkun
Dioprosopa clavatus	United States of America	Arkansas	Hagarville	35.513171	-93.326354	Occurrence Download	https://doi.org/10.15468/dl.3bxkun
Dioprosopa clavatus	United States of America	Oklahoma	Norman	35.205322	-97.421791	Occurrence Download	https://doi.org/10.15468/dl.3bxkun
Dioprosopa clavatus	United States of America	Texas	Harris County	29.866962	-95.778472	Occurrence Download	https://doi.org/10.15468/dl.3bxkun
Dioprosopa clavatus	United States of America	Oklahoma	Newcastle	35.271381	-97.588458	Occurrence Download	https://doi.org/10.15468/dl.3bxkun
Dioprosopa clavatus	United States of America	California	Laguna Beach	33.489056	-117.749742	Occurrence Download	https://doi.org/10.15468/dl.3bxkun

Dioprosopa clavatus	United States of America	Oklahoma	Hughes County	35.140171	-96.442746	Occurrence Download https://doi.org/10.15468/dl.3bxkun
Dioprosopa clavatus	United States of America	Florida	Nassauville	30.566523	-81.52252	Occurrence Download https://doi.org/10.15468/dl.3bxkun
Dioprosopa clavatus	United States of America	Georgia	Skidaway Island	31.957359	-81.025397	Occurrence Download https://doi.org/10.15468/dl.3bxkun
Dioprosopa clavatus	United States of America	Oklahoma	Moore	35.300867	-97.423795	Occurrence Download https://doi.org/10.15468/dl.3bxkun
Dioprosopa clavatus	United States of America	California	Los Angeles County	34.658831	-118.39779	Occurrence Download https://doi.org/10.15468/dl.3bxkun
Dioprosopa clavatus	United States of America	Florida	Leon County	30.443687	-84.139785	Occurrence Download https://doi.org/10.15468/dl.3bxkun
Dioprosopa clavatus	United States of America	Florida	Collier County	25.929188	-80.942845	Occurrence Download https://doi.org/10.15468/dl.3bxkun
Dioprosopa clavatus	United States of America	Texas	Denison	33.739228	-96.752062	Occurrence Download https://doi.org/10.15468/dl.3bxkun
Dioprosopa clavatus	Mexico	Guanajuato	Dolores Hidalgo	21.156956	-100.954786	Occurrence Download https://doi.org/10.15468/dl.3bxkun
Dioprosopa clavatus	United States of America	South Carolina	Port Royal	32.38123	-80.689742	Occurrence Download https://doi.org/10.15468/dl.3bxkun
Dioprosopa clavatus	United States of America	California	Dana Point	33.44448	-117.736355	Occurrence Download https://doi.org/10.15468/dl.3bxkun
Dioprosopa clavatus	United States of America	Florida	Santa Rosa County	30.662451	-87.201714	Occurrence Download https://doi.org/10.15468/dl.3bxkun
Dioprosopa clavatus	United States of America	Oklahoma	Oklahoma City	35.313058	-97.546813	Occurrence Download https://doi.org/10.15468/dl.3bxkun
Dioprosopa clavatus	Mexico	Puebla	San Gabriel Chilac	18.317241	-97.344478	Occurrence Download https://doi.org/10.15468/dl.3bxkun
Dioprosopa clavatus	United States of America	Arizona	Tumacacori	31.567715	-111.051189	Occurrence Download https://doi.org/10.15468/dl.3bxkun
Dioprosopa clavatus	United States of America	California	Dana Point	33.450999	-117.687993	Occurrence Download https://doi.org/10.15468/dl.3bxkun
Dioprosopa clavatus	United States of America	Texas	Hays County	30.043119	-97.813337	Occurrence Download https://doi.org/10.15468/dl.3bxkun
Dioprosopa clavatus	United States of America	Texas	Galveston County	29.403862	-95.119233	Occurrence Download https://doi.org/10.15468/dl.3bxkun
Dioprosopa clavatus	Peru	Lima	Papa León X III	-12.476046	-76.737712	Occurrence Download https://doi.org/10.15468/dl.3bxkun
Dioprosopa clavatus	United States of America	Louisiana	Bossier Parish	32.612168	-93.73298	Occurrence Download https://doi.org/10.15468/dl.3bxkun
Dioprosopa clavatus	United States of America	Texas	Montgomery County	30.262022	-95.502113	Occurrence Download https://doi.org/10.15468/dl.3bxkun
Dioprosopa clavatus	United States of America	Delaware	Kent County	38.971705	-75.386118	Occurrence Download https://doi.org/10.15468/dl.3bxkun
Dioprosopa clavatus	United States of America	Arkansas	Fayetteville	36.107489	-94.201658	Occurrence Download https://doi.org/10.15468/dl.3bxkun
Dioprosopa clavatus	United States of America	Arkansas	Fayetteville	36.107456	-94.201614	Occurrence Download https://doi.org/10.15468/dl.3bxkun
Dioprosopa clavatus	United States of America	Tennessee	Bedford County	35.594238	-86.420865	Occurrence Download https://doi.org/10.15468/dl.3bxkun
Dioprosopa clavatus	United States of America	Arizona	Tumacacori	31.567731	-111.05087	Occurrence Download https://doi.org/10.15468/dl.3bxkun
Dioprosopa clavatus	United States of America	California	Lake Elsinore	33.672291	-117.334017	Occurrence Download https://doi.org/10.15468/dl.3bxkun
Dioprosopa clavatus	United States of America	California	El Cajon	32.742767	-116.939588	Occurrence Download https://doi.org/10.15468/dl.3bxkun
Dioprosopa clavatus	United States of America	Oklahoma	Norman	35.194336	-97.461311	Occurrence Download https://doi.org/10.15468/dl.3bxkun
Dioprosopa clavatus	United States of America	Nebraska	Denton	40.693626	-96.853412	Occurrence Download https://doi.org/10.15468/dl.3bxkun
Dioprosopa clavatus	United States of America	Mississippi	Jones County	31.613057	-89.26965	Occurrence Download https://doi.org/10.15468/dl.3bxkun
Dioprosopa clavatus	United States of America	Florida	Port Saint Lucie	27.273049	-80.358226	Occurrence Download https://doi.org/10.15468/dl.3bxkun
Dioprosopa clavatus	United States of America	Florida	Levy County	29.04192	-82.569516	Occurrence Download https://doi.org/10.15468/dl.3bxkun
Dioprosopa clavatus	United States of America	Florida	Escambia County	30.364806	-87.404307	Occurrence Download https://doi.org/10.15468/dl.3bxkun
Dioprosopa clavatus	United States of America	South Carolina	Charleston	32.722482	-79.955391	Occurrence Download https://doi.org/10.15468/dl.3bxkun
Dioprosopa clavatus	United States of America	Texas	Sweeny	29.0598	-95.7162	Occurrence Download https://doi.org/10.15468/dl.3bxkun
Dioprosopa clavatus	United States of America	Arkansas	Fayetteville	36.136725	-94.1205	Occurrence Download https://doi.org/10.15468/dl.3bxkun
Dioprosopa clavatus	United States of America	California	Mariposa County	37.504678	-119.962745	Occurrence Download https://doi.org/10.15468/dl.3bxkun
Dioprosopa clavatus	United States of America	Florida	Spring Hill	28.500345	-82.520667	Occurrence Download https://doi.org/10.15468/dl.3bxkun

Dioprosopa clavatus	United States of America	Tennessee	Montgomery County	36.615413	-87.272408	Occurrence Download https://doi.org/10.15468/dl.3bxkun
Dioprosopa clavatus	United States of America	California	San Luis Obispo County	35.497814	-120.071773	Occurrence Download https://doi.org/10.15468/dl.3bxkun
Dioprosopa clavatus	United States of America	Arkansas	Bryant	34.592549	-92.515162	Occurrence Download https://doi.org/10.15468/dl.3bxkun
Dioprosopa clavatus	Mexico	Chihuahua	Meoqui	28.261985	-105.496005	Occurrence Download https://doi.org/10.15468/dl.3bxkun
Dioprosopa clavatus	United States of America	Georgia	Euchee Creek	33.466102	-82.230091	Occurrence Download https://doi.org/10.15468/dl.3bxkun
Dioprosopa clavatus	United States of America	California	Lake Elsinore	33.660886	-117.385567	Occurrence Download https://doi.org/10.15468/dl.3bxkun
Dioprosopa clavatus	United States of America	California	Ellykwanan	33.155189	-116.67315	Occurrence Download https://doi.org/10.15468/dl.3bxkun
Dioprosopa clavatus	United States of America	Texas	Midland	31.9617	-102.1237	Occurrence Download https://doi.org/10.15468/dl.3bxkun
Dioprosopa clavatus	United States of America	Tennessee	Mineral Springs	35.092768	-85.527338	Occurrence Download https://doi.org/10.15468/dl.3bxkun
Dioprosopa clavatus	Mexico	Querétaro	El Progreso (Las Trojas)	20.514398	-100.386467	Occurrence Download https://doi.org/10.15468/dl.3bxkun
Dioprosopa clavatus	United States of America	California	Fallbrook	33.3217	-117.237653	Occurrence Download https://doi.org/10.15468/dl.3bxkun
Dioprosopa clavatus	United States of America	Florida	Horizon West	28.484739	-81.622167	Occurrence Download https://doi.org/10.15468/dl.3bxkun
Dioprosopa clavatus	United States of America	Arizona	Sonoita	31.656793	-110.712699	Occurrence Download https://doi.org/10.15468/dl.3bxkun
Dioprosopa clavatus	United States of America	Texas	Collin County	33.321681	-96.660025	Occurrence Download https://doi.org/10.15468/dl.3bxkun
Dioprosopa clavatus	United States of America	Florida	DeLand	29.09433	-81.34951	Occurrence Download https://doi.org/10.15468/dl.3bxkun
Dioprosopa clavatus	United States of America	Texas	Corpus Christi	27.773772	-97.526944	Occurrence Download https://doi.org/10.15468/dl.3bxkun
Dioprosopa clavatus	United States of America	Florida	Jupiter	26.925877	-80.17024	Occurrence Download https://doi.org/10.15468/dl.3bxkun
Dioprosopa clavatus	United States of America	Mississippi	Sunflower County	33.496766	-90.687946	Occurrence Download https://doi.org/10.15468/dl.3bxkun
Dioprosopa clavatus	United States of America	Oklahoma	Norman	35.205389	-97.422019	Occurrence Download https://doi.org/10.15468/dl.3bxkun
Dioprosopa clavatus	United States of America	South Carolina	Congaree	33.910638	-80.810061	Occurrence Download https://doi.org/10.15468/dl.3bxkun
Dioprosopa clavatus	United States of America	Texas	Lubbock	33.622598	-101.891048	Occurrence Download https://doi.org/10.15468/dl.3bxkun
Dioprosopa clavatus	United States of America	Arizona	Maricopa County	33.518564	-111.860529	Occurrence Download https://doi.org/10.15468/dl.3bxkun
Dioprosopa clavatus	United States of America	Texas	Lubbock	33.632578	-101.888221	Occurrence Download https://doi.org/10.15468/dl.3bxkun
Dioprosopa clavatus	United States of America	Florida	Jacksonville	30.143996	-81.649006	Occurrence Download https://doi.org/10.15468/dl.3bxkun
Dioprosopa clavatus	United States of America	Texas	Abilene	32.374122	-99.750983	Occurrence Download https://doi.org/10.15468/dl.3bxkun
Dioprosopa clavatus	United States of America	Texas	Kerr County	29.999398	-99.023399	Occurrence Download https://doi.org/10.15468/dl.3bxkun
Dioprosopa clavatus	United States of America	Texas	Falcon Ranch	29.75074	-95.777985	Occurrence Download https://doi.org/10.15468/dl.3bxkun
Dioprosopa clavatus	United States of America	Tennessee	Montgomery County	36.615269	-87.271422	Occurrence Download https://doi.org/10.15468/dl.3bxkun
Dioprosopa clavatus	United States of America	Texas	Vilas	30.837408	-97.304978	Occurrence Download https://doi.org/10.15468/dl.3bxkun
Dioprosopa clavatus	United States of America	Texas	Vilas	30.837414	-97.304961	Occurrence Download https://doi.org/10.15468/dl.3bxkun
Dioprosopa clavatus	United States of America	Florida	DeLand	29.093918	-81.349821	Occurrence Download https://doi.org/10.15468/dl.3bxkun
Dioprosopa clavatus	United States of America	Nebraska	Lincoln	40.778572	-96.633111	Occurrence Download https://doi.org/10.15468/dl.3bxkun
Dioprosopa clavatus	United States of America	Texas	Georgetown	30.65105	-97.81243	Occurrence Download https://doi.org/10.15468/dl.3bxkun
Dioprosopa clavatus	United States of America	Texas	Cinco Ranch	29.718331	-95.745422	Occurrence Download https://doi.org/10.15468/dl.3bxkun
Dioprosopa clavatus	United States of America	Texas	Sugar Land	29.553352	-95.641323	Occurrence Download https://doi.org/10.15468/dl.3bxkun
Dioprosopa clavatus	United States of America	New Mexico	Bernalillo County	35.156679	-106.439283	Occurrence Download https://doi.org/10.15468/dl.3bxkun
Dioprosopa clavatus	United States of America	Texas	Johnson County	32.303606	-97.225756	Occurrence Download https://doi.org/10.15468/dl.3bxkun
Dioprosopa clavatus	United States of America	Texas	Grey Forest	29.620117	-98.674888	Occurrence Download https://doi.org/10.15468/dl.3bxkun
Dioprosopa clavatus	United States of America	Missouri	Anderson	36.646088	-94.433852	Occurrence Download https://doi.org/10.15468/dl.3bxkun

Dioprosopa clavatus	Peru	Ayacucho	Ahuaruchayocc	-12.669425	-73.814981	Occurrence Download https://doi.org/10.15468/dl.3bxkun
Dioprosopa clavatus	United States of America	Texas	Corinth	33.153955	-97.04096	Occurrence Download https://doi.org/10.15468/dl.3bxkun
Dioprosopa clavatus	United States of America	California	Long Beach	33.761994	-118.196495	Occurrence Download https://doi.org/10.15468/dl.3bxkun
Dioprosopa clavatus	United States of America	Texas	Lubbock	33.620036	-101.892866	Occurrence Download https://doi.org/10.15468/dl.3bxkun
Dioprosopa clavatus	United States of America	Texas	Georgetown	30.619995	-97.702863	Occurrence Download https://doi.org/10.15468/dl.3bxkun
Dioprosopa clavatus	United States of America	Texas	Newt	33.838311	-95.879328	Occurrence Download https://doi.org/10.15468/dl.3bxkun
Dioprosopa clavatus	United States of America	Florida	Port Charlotte	26.999161	-82.076721	Occurrence Download https://doi.org/10.15468/dl.3bxkun
Dioprosopa clavatus	Mexico	Chihuahua	Meoqui	28.261777	-105.499204	Occurrence Download https://doi.org/10.15468/dl.3bxkun
Dioprosopa clavatus	United States of America	Oklahoma	Norman	35.252516	-97.432064	Occurrence Download https://doi.org/10.15468/dl.3bxkun
Dioprosopa clavatus	United States of America	Texas	Indigo Lake Estates	30.166775	-95.763146	Occurrence Download https://doi.org/10.15468/dl.3bxkun
Dioprosopa clavatus	United States of America	Illinois	McLean County	40.550347	-89.124668	Occurrence Download https://doi.org/10.15468/dl.3bxkun
Dioprosopa clavatus	United States of America	South Carolina	Travelers Rest	34.927474	-82.440068	Occurrence Download https://doi.org/10.15468/dl.3bxkun
Dioprosopa clavatus	United States of America	California	San Joaquin County	37.708037	-121.488628	Occurrence Download https://doi.org/10.15468/dl.3bxkun
Dioprosopa clavatus	United States of America	California	Riverside County	33.816104	-117.384025	Occurrence Download https://doi.org/10.15468/dl.3bxkun
Dioprosopa clavatus	United States of America	California	Lincoln	38.878725	-121.257769	Occurrence Download https://doi.org/10.15468/dl.3bxkun
Dioprosopa clavatus	Mexico	Coahuila	Saltillo	25.333578	-100.992886	Occurrence Download https://doi.org/10.15468/dl.3bxkun
Dioprosopa clavatus	United States of America	California	Ventura County	34.086258	-119.036663	Occurrence Download https://doi.org/10.15468/dl.3bxkun
Dioprosopa clavatus	United States of America	Florida	Orange County	28.493573	-81.124071	Occurrence Download https://doi.org/10.15468/dl.3bxkun
Dioprosopa clavatus	United States of America	Kansas	Leavenworth	39.326824	-94.935757	Occurrence Download https://doi.org/10.15468/dl.3bxkun
Dioprosopa clavatus	United States of America	California	Camarillo	34.210756	-118.983757	Occurrence Download https://doi.org/10.15468/dl.3bxkun
Dioprosopa clavatus	United States of America	Texas	Burnet County	30.859319	-98.412621	Occurrence Download https://doi.org/10.15468/dl.3bxkun
Dioprosopa clavatus	United States of America	Florida	Saint Petersburg	27.71352	-82.68705	Occurrence Download https://doi.org/10.15468/dl.3bxkun
Dioprosopa clavatus	United States of America	Texas	Indigo Lake Estates	30.166614	-95.763135	Occurrence Download https://doi.org/10.15468/dl.3bxkun
Dioprosopa clavatus	United States of America	Texas	San Antonio	29.548638	-98.759072	Occurrence Download https://doi.org/10.15468/dl.3bxkun
Dioprosopa clavatus	United States of America	Texas	Indigo Lake Estates	30.166583	-95.763222	Occurrence Download https://doi.org/10.15468/dl.3bxkun
Dioprosopa clavatus	United States of America	Texas	Iowa Colony	29.407978	-95.428797	Occurrence Download https://doi.org/10.15468/dl.3bxkun
Dioprosopa clavatus	United States of America	Texas	Sweeny	29.059472	-95.716206	Occurrence Download https://doi.org/10.15468/dl.3bxkun
Dioprosopa clavatus	United States of America	Texas	Alvin	29.440044	-95.224959	Occurrence Download https://doi.org/10.15468/dl.3bxkun
Dioprosopa clavatus		Texas	Rockport	28.010065	-97.059405	Occurrence Download https://doi.org/10.15468/dl.3bxkun
Dioprosopa clavatus	United States of America	Texas	Prairie Dell	30.912919	-97.553846	Occurrence Download https://doi.org/10.15468/dl.3bxkun
Dioprosopa clavatus	United States of America	Texas	Cameron County	26.053862	-97.629781	Occurrence Download https://doi.org/10.15468/dl.3bxkun
Dioprosopa clavatus	United States of America	Texas	Williamson County	30.663332	-97.381307	Occurrence Download https://doi.org/10.15468/dl.3bxkun
Dioprosopa clavatus	Mexico	Chihuahua	Meoqui	28.262657	-105.498098	Occurrence Download https://doi.org/10.15468/dl.3bxkun
Dioprosopa clavatus	United States of America	Texas	Indigo Lake Estates	30.166629	-95.763151	Occurrence Download https://doi.org/10.15468/dl.3bxkun
Dioprosopa clavatus	United States of America	California	Clovis	36.858817	-119.661728	Occurrence Download https://doi.org/10.15468/dl.3bxkun
Dioprosopa clavatus	United States of America	Texas	Kelliwood Terrace	29.737255	-95.748648	Occurrence Download https://doi.org/10.15468/dl.3bxkun
Dioprosopa clavatus	United States of America	Texas	San Benito	26.104191	-97.626981	Occurrence Download https://doi.org/10.15468/dl.3bxkun
Dioprosopa clavatus	United States of America	Texas	Comal County	29.85984	-98.140809	Occurrence Download https://doi.org/10.15468/dl.3bxkun
Dioprosopa clavatus	United States of America	Arizona	Maricopa County	33.778002	-111.940935	Occurrence Download https://doi.org/10.15468/dl.3bxkun

Dioprosopa clavatus	United States of America	Texas	Frio County	28.932283	-99.060144	Occurrence Download https://doi.org/10.15468/dl.3bxkun
Dioprosopa clavatus	United States of America	Texas	Pearland	29.581544	-95.401167	Occurrence Download https://doi.org/10.15468/dl.3bxkun
Dioprosopa clavatus	United States of America	Texas	Taylor	30.494349	-97.411333	Occurrence Download https://doi.org/10.15468/dl.3bxkun
Dioprosopa clavatus	United States of America	Texas	Mesquite	32.733468	-96.591081	Occurrence Download https://doi.org/10.15468/dl.3bxkun
Dioprosopa clavatus	United States of America	Texas	Edwards County	29.632803	-100.439396	Occurrence Download https://doi.org/10.15468/dl.3bxkun
Dioprosopa clavatus	United States of America	Texas	Jackson County	29.215054	-96.710463	Occurrence Download https://doi.org/10.15468/dl.3bxkun
Dioprosopa clavatus	United States of America	Texas	Calaveras	29.215866	-98.262675	Occurrence Download https://doi.org/10.15468/dl.3bxkun
Dioprosopa clavatus	United States of America	Texas	Hidalgo County	26.078496	-98.137421	Occurrence Download https://doi.org/10.15468/dl.3bxkun
Dioprosopa clavatus	United States of America	Texas	Waco	31.488097	-97.286163	Occurrence Download https://doi.org/10.15468/dl.3bxkun
Dioprosopa clavatus	United States of America	New Mexico	Doña Ana County	32.336804	-106.602465	Occurrence Download https://doi.org/10.15468/dl.3bxkun
Dioprosopa clavatus	United States of America	Texas	Mission	26.179714	-98.366539	Occurrence Download https://doi.org/10.15468/dl.3bxkun
Dioprosopa clavatus	United States of America	California	Fowler	36.632829	-119.630076	Occurrence Download https://doi.org/10.15468/dl.3bxkun
Dioprosopa clavatus	United States of America	New Mexico	Boyd	32.327785	-106.588007	Occurrence Download https://doi.org/10.15468/dl.3bxkun
Dioprosopa clavatus	United States of America	Mississippi	Grenada County	33.745901	-89.997082	Occurrence Download https://doi.org/10.15468/dl.3bxkun
Dioprosopa clavatus	Mexico	Aguascalientes	Municipio de Aguascalientes	21.979916	-102.19662	Occurrence Download https://doi.org/10.15468/dl.3bxkun
Dioprosopa clavatus	United States of America	Texas	Montgomery County	30.124823	-95.687863	Occurrence Download https://doi.org/10.15468/dl.3bxkun
Dioprosopa clavatus	United States of America	Texas	Harris County	29.912795	-95.109773	Occurrence Download https://doi.org/10.15468/dl.3bxkun
Dioprosopa clavatus	United States of America	Texas	Cibolo	29.551033	-98.230738	Occurrence Download https://doi.org/10.15468/dl.3bxkun
Dioprosopa clavatus	Mexico	Chihuahua	Juárez	31.618497	-106.556863	Occurrence Download https://doi.org/10.15468/dl.3bxkun
Dioprosopa clavatus	United States of America	Arizona	Pima County	31.763828	-110.888272	Occurrence Download https://doi.org/10.15468/dl.3bxkun
Dioprosopa clavatus	United States of America	Texas	Denton	33.258268	-97.055871	Occurrence Download https://doi.org/10.15468/dl.3bxkun
Dioprosopa clavatus	United States of America	Texas	Rendon	32.580252	-97.245873	Occurrence Download https://doi.org/10.15468/dl.3bxkun
Dioprosopa clavatus	United States of America	Texas	Pearsall	28.898005	-99.105492	Occurrence Download https://doi.org/10.15468/dl.3bxkun
Dioprosopa clavatus	United States of America	Florida	Alachua County	29.592222	-82.06655	Occurrence Download https://doi.org/10.15468/dl.3bxkun
Dioprosopa clavatus	United States of America	Florida	Miami-Dade County	25.389055	-80.628532	Occurrence Download https://doi.org/10.15468/dl.3bxkun
Dioprosopa clavatus	United States of America	Texas	Bandera County	29.780321	-99.432178	Occurrence Download https://doi.org/10.15468/dl.3bxkun
Dioprosopa clavatus	United States of America	Texas	Mission	26.17991	-98.366807	Occurrence Download https://doi.org/10.15468/dl.3bxkun
Dioprosopa clavatus	United States of America	Texas	Frio County	28.988332	-99.113114	Occurrence Download https://doi.org/10.15468/dl.3bxkun
Dioprosopa clavatus	United States of America	South Carolina	Charleston	32.728442	-79.962255	Occurrence Download https://doi.org/10.15468/dl.3bxkun
Dioprosopa clavatus	United States of America	Texas	Weslaco	26.127102	-97.958093	Occurrence Download https://doi.org/10.15468/dl.3bxkun
Dioprosopa clavatus	United States of America	Texas	Mission	26.179679	-98.366171	Occurrence Download https://doi.org/10.15468/dl.3bxkun
Dioprosopa clavatus	United States of America	Florida	Homestead	25.534485	-80.492923	Occurrence Download https://doi.org/10.15468/dl.3bxkun
Dioprosopa clavatus	United States of America	Texas	Starr County	26.584519	-99.149343	Occurrence Download https://doi.org/10.15468/dl.3bxkun
Dioprosopa clavatus	United States of America	Texas	Mission	26.179774	-98.366011	Occurrence Download https://doi.org/10.15468/dl.3bxkun
Dioprosopa clavatus	Mexico	Chihuahua	Meoqui	28.262499	-105.494854	Occurrence Download https://doi.org/10.15468/dl.3bxkun
Dioprosopa clavatus	Argentina	Córdoba	Pedanía Los Reartes	-31.994941	-64.76257	Occurrence Download https://doi.org/10.15468/dl.3bxkun
Dioprosopa clavatus	Argentina	Misiones	Municipio de San Ignacio	-27.278728	-55.578592	Occurrence Download https://doi.org/10.15468/dl.3bxkun
Dioprosopa clavatus	United States of America	Arizona	Pima County	31.795436	-111.016144	Occurrence Download https://doi.org/10.15468/dl.3bxkun
Dioprosopa clavatus	United States of America	Hawaii	Waipahu	21.356737	-158.021289	Occurrence Download https://doi.org/10.15468/dl.3bxkun

Dioprosopa clavatus	United States of America	Florida	Broward County	26.103314	-80.845559	Occurrence Download https://doi.org/10.15468/dl.3bxkun
Dioprosopa clavatus	United States of America	Arizona	Pima County	32.310329	-110.810662	Occurrence Download https://doi.org/10.15468/dl.3bxkun
Dioprosopa clavatus	Argentina	Chubut	Chacra 252	-43.324676	-65.634674	Occurrence Download https://doi.org/10.15468/dl.3bxkun
Dioprosopa clavatus	Argentina	Córdoba	Cuesta Blanca	-31.479081	-64.58616	Occurrence Download https://doi.org/10.15468/dl.3bxkun
Dioprosopa clavatus		Florida	Florida Ridge	27.604605	-80.38997	Occurrence Download https://doi.org/10.15468/dl.3bxkun
Dioprosopa clavatus	United States of America	Texas	Weslaco	26.126849	-97.957937	Occurrence Download https://doi.org/10.15468/dl.3bxkun
Dioprosopa clavatus	United States of America	Texas	Mission	26.179813	-98.366731	Occurrence Download https://doi.org/10.15468/dl.3bxkun
Dioprosopa clavatus	United States of America	California	Corona	33.853856	-117.481319	Occurrence Download https://doi.org/10.15468/dl.3bxkun
Dioprosopa clavatus	United States of America	California	Los Angeles County	34.067003	-118.594418	Occurrence Download https://doi.org/10.15468/dl.3bxkun
Dioprosopa clavatus	United States of America	Florida	Liberty County	30.45592	-84.971048	Occurrence Download https://doi.org/10.15468/dl.3bxkun
Dioprosopa clavatus	United States of America	Texas	Mission	26.179714	-98.3666	Occurrence Download https://doi.org/10.15468/dl.3bxkun
Dioprosopa clavatus	Colombia	Valle del Cauca	El Dovio	4.508107	-76.237186	Occurrence Download https://doi.org/10.15468/dl.3bxkun
Dioprosopa clavatus	United States of America	Texas	Indigo Lake Estates	30.166567	-95.763072	Occurrence Download https://doi.org/10.15468/dl.3bxkun
Dioprosopa clavatus	Argentina	Córdoba	El Durazno	-31.367499	-64.62852	Occurrence Download https://doi.org/10.15468/dl.3bxkun
Dioprosopa clavatus	Argentina	Córdoba	Pedanía Los Reartes	-32.002669	-64.774021	Occurrence Download https://doi.org/10.15468/dl.3bxkun
Dioprosopa clavatus	United States of America	Florida	Wedgefield	28.509727	-81.071	Occurrence Download https://doi.org/10.15468/dl.3bxkun
Dioprosopa clavatus	United States of America	Florida	Sarasota County	27.192647	-82.441485	Occurrence Download https://doi.org/10.15468/dl.3bxkun
Dioprosopa clavatus	Mexico	Jalisco	Zapopan	20.718543	-103.526712	Occurrence Download https://doi.org/10.15468/dl.3bxkun
Dioprosopa clavatus	Argentina	Santa Fe	Bigand	-33.383041	-61.183938	Occurrence Download https://doi.org/10.15468/dl.3bxkun
Dioprosopa clavatus	Mexico	Querétaro	Corregidora	20.522039	-100.388136	Occurrence Download https://doi.org/10.15468/dl.3bxkun
Dioprosopa clavatus	Argentina	La Pampa	Barrio Sur	-35.702795	-63.738249	Occurrence Download https://doi.org/10.15468/dl.3bxkun
Dioprosopa clavatus	Brazil	Tocantins	Monte Santo do Tocantins	-10.123407	-48.951769	Occurrence Download https://doi.org/10.15468/dl.3bxkun
Dioprosopa clavatus	Argentina	Santa Fe	San Jerónimo Norte	-31.545546	-61.065616	Occurrence Download https://doi.org/10.15468/dl.3bxkun
Dioprosopa clavatus	Argentina	Córdoba	Pedanía Rosario	-31.254497	-64.603426	Occurrence Download https://doi.org/10.15468/dl.3bxkun
Dioprosopa clavatus	Brazil	São Paulo	Extrema	-22.904597	-46.344305	Occurrence Download https://doi.org/10.15468/dl.3bxkun
Dioprosopa clavatus	Argentina	Córdoba	Cuesta Blanca	-31.490257	-64.604088	Occurrence Download https://doi.org/10.15468/dl.3bxkun
Dioprosopa clavatus	Argentina	Córdoba	Cuesta Blanca	-31.490258	-64.604175	Occurrence Download https://doi.org/10.15468/dl.3bxkun
Dioprosopa clavatus	Argentina	Córdoba	Cuesta Blanca	-31.479072	-64.586323	Occurrence Download https://doi.org/10.15468/dl.3bxkun
Dioprosopa clavatus	Argentina	Buenos Aires	San Miguel del Monte	-35.441125	-58.807094	Occurrence Download https://doi.org/10.15468/dl.3bxkun
Dioprosopa clavatus	Argentina	Quilmes	Buenos Aires	-34.633429	-58.203751	Occurrence Download https://doi.org/10.15468/dl.3bxkun
Dioprosopa clavatus	Mexico	México	Santa María Cuevas	19.869216	-99.09556	Occurrence Download https://doi.org/10.15468/dl.3bxkun
Dioprosopa clavatus	Mexico	México	Santa María Cuevas	19.869247	-99.095548	Occurrence Download https://doi.org/10.15468/dl.3bxkun
Dioprosopa clavatus	Colombia	Cesar	Pueblo Bello	10.418336	-73.55	Occurrence Download https://doi.org/10.15468/dl.3bxkun
Dioprosopa clavatus	Argentina	Santa Fe		-31.550832	-61.067664	Occurrence Download https://doi.org/10.15468/dl.3bxkun
Dioprosopa clavatus	Costa Rica	Heredia	Varablanca	10.194272	-84.155832	Occurrence Download https://doi.org/10.15468/dl.3bxkun
Dioprosopa clavatus	Argentina	Córdoba	Pedanía Los Reartes	-32.000495	-64.75516	Occurrence Download https://doi.org/10.15468/dl.3bxkun
Dioprosopa clavatus		Florida	Longboat Key	27.368673	-82.625161	Occurrence Download https://doi.org/10.15468/dl.3bxkun
Dioprosopa clavatus	United States of America	Florida	Miami-Dade County	25.76272	-80.498443	Occurrence Download https://doi.org/10.15468/dl.3bxkun
Dioprosopa clavatus	United States of America	Texas	Jeff Davis County	30.541192	-103.835258	Occurrence Download https://doi.org/10.15468/dl.3bxkun

Dioprosopa clavatus	Mexico	Chihuahua	Meoqui	28.273103	-105.471538	Occurrence Download https://doi.org/10.15468/dl.3bxkun
Dioprosopa clavatus	Argentina	Mendoza	Distrito San Isidro	-33.519431	-68.663405	Occurrence Download https://doi.org/10.15468/dl.3bxkun
Dioprosopa clavatus	United States of America	Florida	Homestead	25.72902	-80.49277	Occurrence Download https://doi.org/10.15468/dl.3bxkun
Dioprosopa clavatus	Mexico	Chihuahua	Meoqui	28.262304	-105.495107	Occurrence Download https://doi.org/10.15468/dl.3bxkun
Dioprosopa clavatus	Argentina	Quilmes	Buenos Aires	-34.529056	-58.457292	Occurrence Download https://doi.org/10.15468/dl.3bxkun
Dioprosopa clavatus	United States of America	Florida	Broward County	26.161896	-80.38749	Occurrence Download https://doi.org/10.15468/dl.3bxkun
Dioprosopa clavatus	Mexico	Chihuahua	Meoqui	28.265717	-105.489533	Occurrence Download https://doi.org/10.15468/dl.3bxkun
Dioprosopa clavatus	Mexico	Chihuahua	Meoqui	28.273083	-105.471505	Occurrence Download https://doi.org/10.15468/dl.3bxkun
Dioprosopa clavatus	Mexico	Querétaro	Corregidora	20.522169	-100.387938	Occurrence Download https://doi.org/10.15468/dl.3bxkun
Dioprosopa clavatus	Mexico	Chihuahua	Meoqui	28.273092	-105.47154	Occurrence Download https://doi.org/10.15468/dl.3bxkun
Dioprosopa clavatus	Mexico	Veracruz	Xalapa	19.519953	-96.943402	Occurrence Download https://doi.org/10.15468/dl.3bxkun
Dioprosopa clavatus	Chile	Región Metropolitana de Santiago	San José de Maipo	-33.765838	-70.279092	Occurrence Download https://doi.org/10.15468/dl.3bxkun
Dioprosopa clavatus	United States of America	Texas	Bastrop County	30.0855	-97.17087	Occurrence Download https://doi.org/10.15468/dl.3bxkun
Dioprosopa clavatus	United States of America	Florida	Lee County	26.534935	-81.828525	Occurrence Download https://doi.org/10.15468/dl.3bxkun
Dioprosopa clavatus	United States of America	Texas	Clay County	33.759718	-98.387596	Occurrence Download https://doi.org/10.15468/dl.3bxkun
Dioprosopa clavatus	United States of America	Florida	Fortymile Bend	25.765014	-80.830559	Occurrence Download https://doi.org/10.15468/dl.3bxkun
Dioprosopa clavatus	Trinidad and Tobago	Chaguanas	Chaguanas	10.519955	-61.409882	Occurrence Download https://doi.org/10.15468/dl.3bxkun
Dioprosopa clavatus	Mexico	Sinaloa	Mazatlán	23.270388	-106.457663	Occurrence Download https://doi.org/10.15468/dl.3bxkun
Dioprosopa clavatus	United States of America	Florida	Wellington	26.631192	-80.232445	Occurrence Download https://doi.org/10.15468/dl.3bxkun
Dioprosopa clavatus	United States of America	Texas	Brownsville	25.996717	-97.568528	Occurrence Download https://doi.org/10.15468/dl.3bxkun
Dioprosopa clavatus	United States of America	Arizona	Yavapai County	34.829735	-111.897282	Occurrence Download https://doi.org/10.15468/dl.3bxkun
Dioprosopa clavatus	United States of America	Texas	Texas City	29.449517	-94.92843	Occurrence Download https://doi.org/10.15468/dl.3bxkun
Dioprosopa clavatus	United States of America	Florida	Alachua County	29.681751	-82.487652	Occurrence Download https://doi.org/10.15468/dl.3bxkun
Dioprosopa clavatus	United States of America	Florida	Wellington	26.631112	-80.2325	Occurrence Download https://doi.org/10.15468/dl.3bxkun
Dioprosopa clavatus	Mexico	Chihuahua	Meoqui	28.269892	-105.468931	Occurrence Download https://doi.org/10.15468/dl.3bxkun
Dioprosopa clavatus	United States of America	California	Calabasas	34.136619	-118.631142	Occurrence Download https://doi.org/10.15468/dl.3bxkun
Dioprosopa clavatus	United States of America	Arizona	Pima County	32.281422	-110.7307	Occurrence Download https://doi.org/10.15468/dl.3bxkun
Dioprosopa clavatus	United States of America	Texas	Buda	30.092642	-97.837604	Occurrence Download https://doi.org/10.15468/dl.3bxkun
Dioprosopa clavatus	Mexico	Jalisco	El Romereño (INFONAVIT)	20.379665	-102.960528	Occurrence Download https://doi.org/10.15468/dl.3bxkun
Dioprosopa clavatus	United States of America	Florida	Yulee	30.625949	-81.578522	Occurrence Download https://doi.org/10.15468/dl.3bxkun
Dioprosopa clavatus	United States of America	Florida	Broward County	26.199273	-80.345581	Occurrence Download https://doi.org/10.15468/dl.3bxkun
Dioprosopa clavatus	United States of America	Texas	Blanco County	30.278771	-98.306491	Occurrence Download https://doi.org/10.15468/dl.3bxkun
Dioprosopa clavatus	Mexico	Chihuahua	Loreto	28.315919	-105.426935	Occurrence Download https://doi.org/10.15468/dl.3bxkun
Dioprosopa clavatus	Costa Rica	Cartago	San Rafael	9.906917	-83.975789	Occurrence Download https://doi.org/10.15468/dl.3bxkun
Dioprosopa clavatus	United States of America	Texas	Grey Forest	29.619959	-98.674559	Occurrence Download https://doi.org/10.15468/dl.3bxkun
Dioprosopa clavatus	United States of America	Texas	Vilas	30.838281	-97.304572	Occurrence Download https://doi.org/10.15468/dl.3bxkun
Dioprosopa clavatus	United States of America	Texas	Comal County	29.859828	-98.140587	Occurrence Download https://doi.org/10.15468/dl.3bxkun
Dioprosopa clavatus	Mexico	Nayarit	Tepic	21.479417	-104.863972	Occurrence Download https://doi.org/10.15468/dl.3bxkun
Dioprosopa clavatus	Mexico	Tamaulipas	Los Troncones	23.771667	-99.263889	Occurrence Download https://doi.org/10.15468/dl.3bxkun

Dioprosopa clavatus	United States of America	Texas	Pearland	29.535565	-95.289457	Occurrence Download https://doi.org/10.15468/dl.3bxkun
Dioprosopa clavatus	United States of America	Texas	Blanco County	30.430358	-98.17383	Occurrence Download https://doi.org/10.15468/dl.3bxkun
Dioprosopa clavatus	United States of America	Texas	New Braunfels	29.675742	-98.109422	Occurrence Download https://doi.org/10.15468/dl.3bxkun
Dioprosopa clavatus	United States of America	Texas	Dripping Springs	30.203994	-98.08012	Occurrence Download https://doi.org/10.15468/dl.3bxkun
Dioprosopa clavatus	United States of America	Arkansas	Hagarville	35.513164	-93.32621	Occurrence Download https://doi.org/10.15468/dl.3bxkun
Dioprosopa clavatus	United States of America	Oklahoma	Norman	35.253519	-97.43518	Occurrence Download https://doi.org/10.15468/dl.3bxkun
Dioprosopa clavatus	United States of America	Texas	Georgetown	30.696471	-97.823675	Occurrence Download https://doi.org/10.15468/dl.3bxkun
Dioprosopa clavatus	United States of America	Florida	Okaloosa County	30.575012	-86.607388	Occurrence Download https://doi.org/10.15468/dl.3bxkun
Dioprosopa clavatus	United States of America	Texas	Jones County	32.711435	-99.786223	Occurrence Download https://doi.org/10.15468/dl.3bxkun
Dioprosopa clavatus	United States of America	Texas	Fort Bend County	29.498686	-95.504302	Occurrence Download https://doi.org/10.15468/dl.3bxkun
Dioprosopa clavatus	United States of America	Texas	Fort Bend County	29.494029	-95.518297	Occurrence Download https://doi.org/10.15468/dl.3bxkun
Dioprosopa clavatus	United States of America	Texas	Bell County	31.226359	-97.473895	Occurrence Download https://doi.org/10.15468/dl.3bxkun
Dioprosopa clavatus	United States of America	Texas	Denton	33.146832	-97.096041	Occurrence Download https://doi.org/10.15468/dl.3bxkun
Dioprosopa clavatus	United States of America	Texas	Bandera County	29.75211	-99.439732	Occurrence Download https://doi.org/10.15468/dl.3bxkun
Dioprosopa clavatus	United States of America	Texas	Alvin	29.439722	-95.224722	Occurrence Download https://doi.org/10.15468/dl.3bxkun
Dioprosopa clavatus	United States of America	Texas	Georgetown	30.632791	-97.67475	Occurrence Download https://doi.org/10.15468/dl.3bxkun
Dioprosopa clavatus	United States of America	Oklahoma	Osage County	36.425021	-96.14132	Occurrence Download https://doi.org/10.15468/dl.3bxkun
Dioprosopa clavatus	United States of America	North Carolina	Durham	35.903562	-78.95053	Occurrence Download https://doi.org/10.15468/dl.3bxkun
Dioprosopa clavatus	United States of America	Florida	Yulee	30.631879	-81.606487	Occurrence Download https://doi.org/10.15468/dl.3bxkun
Dioprosopa clavatus	United States of America	Texas	Murphy	33.024746	-96.598896	Occurrence Download https://doi.org/10.15468/dl.3bxkun
Dioprosopa clavatus	United States of America	Florida	Narcoossee	28.302538	-81.231737	Occurrence Download https://doi.org/10.15468/dl.3bxkun
Dioprosopa clavatus	United States of America	Texas	McKinney	33.160092	-96.615233	Occurrence Download https://doi.org/10.15468/dl.3bxkun
Dioprosopa clavatus	United States of America	Texas	Travis County	30.454918	-97.83667	Occurrence Download https://doi.org/10.15468/dl.3bxkun
Dioprosopa clavatus	Mexico	Chihuahua	Meoqui	28.263158	-105.473473	Occurrence Download https://doi.org/10.15468/dl.3bxkun
Dioprosopa clavatus	United States of America	Texas	Mission	26.180404	-98.3308	Occurrence Download https://doi.org/10.15468/dl.3bxkun
Dioprosopa clavatus	Mexico	Querétaro	Corregidora	20.522095	-100.387923	Occurrence Download https://doi.org/10.15468/dl.3bxkun
Dioprosopa clavatus	Mexico	Querétaro	Cadereyta de Montes	20.686051	-99.803628	Occurrence Download https://doi.org/10.15468/dl.3bxkun
Dioprosopa clavatus	Mexico	Guanajuato	Acámbaro	20.028964	-100.721361	Occurrence Download https://doi.org/10.15468/dl.3bxkun
Dioprosopa clavatus	United States of America	Texas	Brazoria County	29.540881	-95.378144	Occurrence Download https://doi.org/10.15468/dl.3bxkun
Dioprosopa clavatus	Mexico	Michoacán	Jacona de Plancarte	19.942807	-102.293685	Occurrence Download https://doi.org/10.15468/dl.3bxkun
Dioprosopa clavatus	United States of America	Texas	Tivoli	28.456974	-96.888024	Occurrence Download https://doi.org/10.15468/dl.3bxkun
Dioprosopa clavatus	Mexico	Chihuahua	Meoqui	28.262627	-105.481847	Occurrence Download https://doi.org/10.15468/dl.3bxkun
Dioprosopa clavatus	Mexico	Chihuahua	Meoqui	28.262357	-105.482218	Occurrence Download https://doi.org/10.15468/dl.3bxkun
Dioprosopa clavatus	United States of America	California	Golden Hills	35.138651	-118.483507	Occurrence Download https://doi.org/10.15468/dl.3bxkun
Dioprosopa clavatus	United States of America	Texas	Refugio County	28.469901	-96.986026	Occurrence Download https://doi.org/10.15468/dl.3bxkun
Dioprosopa clavatus	United States of America	Texas	Bastrop County	30.140093	-97.459093	Occurrence Download https://doi.org/10.15468/dl.3bxkun
Dioprosopa clavatus	United States of America	Texas	Baytown	29.810408	-94.893647	Occurrence Download https://doi.org/10.15468/dl.3bxkun
Dioprosopa clavatus	United States of America	Texas	Murphy	33.02513	-96.599295	Occurrence Download https://doi.org/10.15468/dl.3bxkun
Dioprosopa clavatus	United States of America	Texas	Frisco	33.177016	-96.817578	Occurrence Download https://doi.org/10.15468/dl.3bxkun

Dioprosopa clavatus	United States of America	Oklahoma	Norman	35.253778	-97.434639	Occurrence Download https://doi.org/10.15468/dl.3bxkun
Dioprosopa clavatus	United States of America	Texas	Vilas	30.837458	-97.305039	Occurrence Download https://doi.org/10.15468/dl.3bxkun
Dioprosopa clavatus	United States of America	Arizona	Pima County	32.204045	-111.114942	Occurrence Download https://doi.org/10.15468/dl.3bxkun
Dioprosopa clavatus	United States of America	Texas	Rosenberg	29.537437	-95.748567	Occurrence Download https://doi.org/10.15468/dl.3bxkun
Dioprosopa clavatus	United States of America	California	Golden Hills	35.133733	-118.492643	Occurrence Download https://doi.org/10.15468/dl.3bxkun
Dioprosopa clavatus	United States of America	Oklahoma	Norman	35.252939	-97.433314	Occurrence Download https://doi.org/10.15468/dl.3bxkun
Dioprosopa clavatus	United States of America	Florida	Durant	27.906091	-82.192615	Occurrence Download https://doi.org/10.15468/dl.3bxkun
Dioprosopa clavatus	United States of America	Texas	Iowa Colony	29.414873	-95.416008	Occurrence Download https://doi.org/10.15468/dl.3bxkun
Dioprosopa clavatus	United States of America	Florida	Brevard County	28.046703	-80.788289	Occurrence Download https://doi.org/10.15468/dl.3bxkun
Dioprosopa clavatus	United States of America	Oklahoma	Moore	35.300812	-97.423849	Occurrence Download https://doi.org/10.15468/dl.3bxkun
Dioprosopa clavatus	United States of America	Arizona	Tucson	32.172845	-110.824847	Occurrence Download https://doi.org/10.15468/dl.3bxkun
Dioprosopa clavatus	United States of America	Texas	Uvalde County	29.187774	-99.833363	Occurrence Download https://doi.org/10.15468/dl.3bxkun
Dioprosopa clavatus	United States of America	Texas	Georgetown	30.616658	-97.672677	Occurrence Download https://doi.org/10.15468/dl.3bxkun
Dioprosopa clavatus	United States of America	Arizona	Sahuarita	31.963059	-110.945265	Occurrence Download https://doi.org/10.15468/dl.3bxkun
Dioprosopa clavatus	United States of America	Maryland	Clarksburg	39.24172	-77.239587	Occurrence Download https://doi.org/10.15468/dl.3bxkun
Dioprosopa clavatus	United States of America	Texas	Lubbock County	33.421833	-101.994333	Occurrence Download https://doi.org/10.15468/dl.3bxkun
Dioprosopa clavatus	Mexico	Jalisco	Poncitlán	20.380425	-102.930666	Occurrence Download https://doi.org/10.15468/dl.3bxkun
Dioprosopa clavatus	United States of America	California	Unincorporated Santa Monica Mountains	34.102547	-118.706653	Occurrence Download https://doi.org/10.15468/dl.3bxkun
Dioprosopa clavatus	United States of America	Arizona	Pima County	32.246855	-111.135479	Occurrence Download https://doi.org/10.15468/dl.3bxkun
Dioprosopa clavatus	United States of America	Arizona	Picture Rocks	32.368795	-111.1624	Occurrence Download https://doi.org/10.15468/dl.3bxkun
Dioprosopa clavatus	United States of America	Colorado	Colorado Springs	38.799719	-104.859148	Occurrence Download https://doi.org/10.15468/dl.3bxkun
Dioprosopa clavatus	United States of America	Virginia	Culpeper County	38.476909	-77.780907	Occurrence Download https://doi.org/10.15468/dl.3bxkun
Dioprosopa clavatus	United States of America	Texas	Woodway	31.489726	-97.230241	Occurrence Download https://doi.org/10.15468/dl.3bxkun
Dioprosopa clavatus	United States of America	Arizona	Pima County	32.291825	-111.097825	Occurrence Download https://doi.org/10.15468/dl.3bxkun
Dioprosopa clavatus	United States of America	Texas	Briscoe County	34.416177	-101.065696	Occurrence Download https://doi.org/10.15468/dl.3bxkun
Dioprosopa clavatus	United States of America	Tennessee	Northaven	35.260376	-90.063355	Occurrence Download https://doi.org/10.15468/dl.3bxkun
Dioprosopa clavatus	United States of America	Texas	Saginaw	32.873633	-97.356575	Occurrence Download https://doi.org/10.15468/dl.3bxkun
Dioprosopa clavatus	United States of America	Texas	Fairchilds	29.46255	-95.758267	Occurrence Download https://doi.org/10.15468/dl.3bxkun
Dioprosopa clavatus	United States of America	New Hampshire	East Kingston	42.927616	-70.990151	Occurrence Download https://doi.org/10.15468/dl.3bxkun
Dioprosopa clavatus	United States of America	Arizona	Pima County	32.318873	-111.134379	Occurrence Download https://doi.org/10.15468/dl.3bxkun
Dioprosopa clavatus	United States of America	Texas	Bexar County	29.6438	-98.700378	Occurrence Download https://doi.org/10.15468/dl.3bxkun
Dioprosopa clavatus	United States of America	Maryland	Upper Marlboro	38.815416	-76.822394	Occurrence Download https://doi.org/10.15468/dl.3bxkun
Dioprosopa clavatus	United States of America	Texas	Bexar County	29.403395	-98.745073	Occurrence Download https://doi.org/10.15468/dl.3bxkun
Dioprosopa clavatus	United States of America	Texas	Hays County	30.134985	-97.977028	Occurrence Download https://doi.org/10.15468/dl.3bxkun
Dioprosopa clavatus	United States of America	Florida	Woods	30.355864	-84.985718	Occurrence Download https://doi.org/10.15468/dl.3bxkun
Dioprosopa clavatus	United States of America	Florida	Miami Beach	25.851018	-80.134351	Occurrence Download https://doi.org/10.15468/dl.3bxkun
Dioprosopa clavatus	Mexico	Chihuahua	Meoqui	28.273072	-105.471537	Occurrence Download https://doi.org/10.15468/dl.3bxkun
Dioprosopa clavatus	Mexico	Baja California Sur	San José del Cabo	23.057851	-109.715145	Occurrence Download https://doi.org/10.15468/dl.3bxkun
Dioprosopa clavatus	Mexico	Chihuahua	Meoqui	28.262827	-105.481375	Occurrence Download https://doi.org/10.15468/dl.3bxkun

Dioprosopa clavatus	Mexico	Chihuahua	Meoqui	28.273069	-105.471595	Occurrence Download https://doi.org/10.15468/dl.3bxkun
Dioprosopa clavatus	Mexico	Querétaro	Cadereyta de Montes	20.686666	-99.804928	Occurrence Download https://doi.org/10.15468/dl.3bxkun
Dioprosopa clavatus	Mexico	Chihuahua	Meoqui	28.261011	-105.497517	Occurrence Download https://doi.org/10.15468/dl.3bxkun
Dioprosopa clavatus	United States of America	Nebraska	Cass County	41.024061	-96.312368	Occurrence Download https://doi.org/10.15468/dl.3bxkun
Dioprosopa clavatus	United States of America	Texas	Manvel	29.532203	-95.402183	Occurrence Download https://doi.org/10.15468/dl.3bxkun
Dioprosopa clavatus	United States of America	Florida	Pinellas County	27.938452	-82.678806	Occurrence Download https://doi.org/10.15468/dl.3bxkun
Dioprosopa clavatus	United States of America	Texas	Rosenberg	29.533586	-95.828961	Occurrence Download https://doi.org/10.15468/dl.3bxkun
Dioprosopa clavatus	United States of America	Texas	Tom Green County	31.537356	-100.541058	Occurrence Download https://doi.org/10.15468/dl.3bxkun
Dioprosopa clavatus	United States of America	Texas	Little Elm	33.217636	-96.943401	Occurrence Download https://doi.org/10.15468/dl.3bxkun
Dioprosopa clavatus	United States of America	California	Riverside	33.897301	-117.297148	Occurrence Download https://doi.org/10.15468/dl.3bxkun
Dioprosopa clavatus	United States of America	Texas	Kerr County	30.064913	-99.048408	Occurrence Download https://doi.org/10.15468/dl.3bxkun
Dioprosopa clavatus	United States of America	Missouri	Lee's Summit	38.911766	-94.345634	Occurrence Download https://doi.org/10.15468/dl.3bxkun
Dioprosopa clavatus	United States of America	Texas	Arlington	32.639094	-97.070467	Occurrence Download https://doi.org/10.15468/dl.3bxkun
Dioprosopa clavatus	United States of America	Arizona	Pima County	32.302863	-110.486503	Occurrence Download https://doi.org/10.15468/dl.3bxkun
Dioprosopa clavatus	United States of America	California	Golden Hills	35.144775	-118.480611	Occurrence Download https://doi.org/10.15468/dl.3bxkun
Dioprosopa clavatus	United States of America	California	Butte County	39.335918	-121.845241	Occurrence Download https://doi.org/10.15468/dl.3bxkun
Dioprosopa clavatus	United States of America	California	Riverside County	33.798334	-117.377875	Occurrence Download https://doi.org/10.15468/dl.3bxkun
Dioprosopa clavatus	United States of America	Florida	Destin	30.407576	-86.50043	Occurrence Download https://doi.org/10.15468/dl.3bxkun
Dioprosopa clavatus	United States of America	Indiana	Hendricks County	39.848789	-86.329768	Occurrence Download https://doi.org/10.15468/dl.3bxkun
Dioprosopa clavatus	United States of America	California	Santee	32.890797	-116.846215	Occurrence Download https://doi.org/10.15468/dl.3bxkun
Dioprosopa clavatus	United States of America	Florida	Osprey	27.1704	-82.473233	Occurrence Download https://doi.org/10.15468/dl.3bxkun
Dioprosopa clavatus	United States of America	Texas	Odessa	31.957682	-102.27966	Occurrence Download https://doi.org/10.15468/dl.3bxkun
Dioprosopa clavatus	United States of America	Illinois	Chicago	41.96412	-87.633683	Occurrence Download https://doi.org/10.15468/dl.3bxkun
Dioprosopa clavatus	United States of America	Texas	Austin	30.159763	-97.845985	Occurrence Download https://doi.org/10.15468/dl.3bxkun
Dioprosopa clavatus	United States of America	Florida	Yulee	30.637038	-81.568409	Occurrence Download https://doi.org/10.15468/dl.3bxkun
Dioprosopa clavatus	United States of America	Kansas	Gardner	38.816191	-94.95598	Occurrence Download https://doi.org/10.15468/dl.3bxkun
Dioprosopa clavatus	United States of America	Florida	Yulee	30.627684	-81.610477	Occurrence Download https://doi.org/10.15468/dl.3bxkun
Dioprosopa clavatus	United States of America	Alabama	Double Springs	34.164707	-87.397964	Occurrence Download https://doi.org/10.15468/dl.3bxkun
Dioprosopa clavatus	United States of America	Texas	Kelliwood Terrace	29.737223	-95.748431	Occurrence Download https://doi.org/10.15468/dl.3bxkun
Dioprosopa clavatus	United States of America	Florida	Destin	30.407133	-86.500121	Occurrence Download https://doi.org/10.15468/dl.3bxkun
Dioprosopa clavatus	United States of America	Arizona	Goodyear	33.295585	-112.436022	Occurrence Download https://doi.org/10.15468/dl.3bxkun
Dioprosopa clavatus	United States of America	California	Santee	32.892247	-116.846842	Occurrence Download https://doi.org/10.15468/dl.3bxkun
Dioprosopa clavatus	United States of America	California	Perris	33.766946	-117.207857	Occurrence Download https://doi.org/10.15468/dl.3bxkun
Dioprosopa clavatus	United States of America	Texas	Lago Vista	30.444863	-98.014131	Occurrence Download https://doi.org/10.15468/dl.3bxkun
Dioprosopa clavatus	United States of America	Texas	Grapeland	31.517528	-95.499687	Occurrence Download https://doi.org/10.15468/dl.3bxkun
Dioprosopa clavatus	United States of America	Texas	Midland	31.965467	-102.120423	Occurrence Download https://doi.org/10.15468/dl.3bxkun
Dioprosopa clavatus	United States of America	Mississippi	Magenta	33.427296	-90.958482	Occurrence Download https://doi.org/10.15468/dl.3bxkun
Dioprosopa clavatus	United States of America	Arizona	Maricopa County	33.534939	-111.783228	Occurrence Download https://doi.org/10.15468/dl.3bxkun
Dioprosopa clavatus	United States of America	Arizona	Pima County	32.338334	-110.688242	Occurrence Download https://doi.org/10.15468/dl.3bxkun

Dioprosopa clavatus	United States of America	Florida	Miami-Dade County	25.76214	-80.499216	Occurrence Download https://doi.org/10.15468/dl.3bxkun
Dioprosopa clavatus	United States of America	Florida	Spring Hill	28.49565	-82.547713	Occurrence Download https://doi.org/10.15468/dl.3bxkun
Dioprosopa clavatus	Mexico	Chihuahua	El Torreón	28.319852	-105.425781	Occurrence Download https://doi.org/10.15468/dl.3bxkun
Dioprosopa clavatus	Mexico	Jalisco	Zapotlán del Rey	20.409218	-102.928288	Occurrence Download https://doi.org/10.15468/dl.3bxkun
Dioprosopa clavatus	Mexico	Sonora	Heroica Nogales	31.257586	-110.961301	Occurrence Download https://doi.org/10.15468/dl.3bxkun
Dioprosopa clavatus	United States of America	California	Butte County	39.33546	-121.845271	Occurrence Download https://doi.org/10.15468/dl.3bxkun
Dioprosopa clavatus	United States of America	Texas	Hearne	30.885558	-96.583747	Occurrence Download https://doi.org/10.15468/dl.3bxkun
Dioprosopa clavatus	United States of America	Texas	Manvel	29.532215	-95.402108	Occurrence Download https://doi.org/10.15468/dl.3bxkun
Dioprosopa clavatus	United States of America	Texas	Georgetown	30.630912	-97.643604	Occurrence Download https://doi.org/10.15468/dl.3bxkun
Dioprosopa clavatus	United States of America	Oklahoma	Stony Point	35.845466	-94.678889	Occurrence Download https://doi.org/10.15468/dl.3bxkun
Dioprosopa clavatus	United States of America	Texas	Corpus Christi	27.661235	-97.353478	Occurrence Download https://doi.org/10.15468/dl.3bxkun
Dioprosopa clavatus	United States of America	Texas	Cameron County	26.142014	-97.742215	Occurrence Download https://doi.org/10.15468/dl.3bxkun
Dioprosopa clavatus	United States of America	Texas	Lucas	33.087612	-96.57622	Occurrence Download https://doi.org/10.15468/dl.3bxkun
Dioprosopa clavatus	United States of America	Texas	Cameron County	26.085765	-97.714744	Occurrence Download https://doi.org/10.15468/dl.3bxkun
Dioprosopa clavatus	United States of America	Texas	Cameron County	26.114847	-97.699463	Occurrence Download https://doi.org/10.15468/dl.3bxkun
Dioprosopa clavatus	United States of America	Texas	Mount Sharp	30.15741	-98.179032	Occurrence Download https://doi.org/10.15468/dl.3bxkun
Dioprosopa clavatus	United States of America	Oklahoma	Owasso	36.272696	-95.835384	Occurrence Download https://doi.org/10.15468/dl.3bxkun
Dioprosopa clavatus	United States of America	California	Rancho Santa Fe	33.019736	-117.161299	Occurrence Download https://doi.org/10.15468/dl.3bxkun
Dioprosopa clavatus	Argentina	Santa Fe	Municipio de Villa Constitución	-33.281129	-60.291439	Occurrence Download https://doi.org/10.15468/dl.3bxkun
Dioprosopa clavatus	United States of America	Texas	Vilas	30.838172	-97.304864	Occurrence Download https://doi.org/10.15468/dl.3bxkun
Dioprosopa clavatus	United States of America	Arkansas	Hagarville	35.507948	-93.320993	Occurrence Download https://doi.org/10.15468/dl.3bxkun
Dioprosopa clavatus	Mexico	Chihuahua	Meoqui	28.27307	-105.47157	Occurrence Download https://doi.org/10.15468/dl.3bxkun
Dioprosopa clavatus	United States of America	South Carolina	Charleston	32.727479	-79.953872	Occurrence Download https://doi.org/10.15468/dl.3bxkun
Dioprosopa clavatus	Brazil	Minas Gerais	Janaúba	-15.837777	-43.304302	Occurrence Download https://doi.org/10.15468/dl.3bxkun
Dioprosopa clavatus	Mexico	Chihuahua	Meoqui	28.273066	-105.471604	Occurrence Download https://doi.org/10.15468/dl.3bxkun
Dioprosopa clavatus	United States of America	Virginia	Culpeper County	38.426192	-77.866909	Occurrence Download https://doi.org/10.15468/dl.3bxkun
Dioprosopa clavatus	United States of America	California	Montecito	34.457884	-119.654399	Occurrence Download https://doi.org/10.15468/dl.3bxkun
Dioprosopa clavatus	United States of America	Florida	Dummit Grove	28.705927	-80.720471	Occurrence Download https://doi.org/10.15468/dl.3bxkun
Dioprosopa clavatus	Mexico	Chihuahua	Colonia Francisco Portillo (Los Jáquez)	28.275553	-105.453821	Occurrence Download https://doi.org/10.15468/dl.3bxkun
Dioprosopa clavatus	United States of America	Louisiana	St. George	30.357307	-91.095489	Occurrence Download https://doi.org/10.15468/dl.3bxkun
Dioprosopa clavatus	United States of America	Tamaulipas	Mission	26.183669	-98.380528	Occurrence Download https://doi.org/10.15468/dl.3bxkun
Dioprosopa clavatus	United States of America	Arizona	Sonoita	31.656899	-110.713148	Occurrence Download https://doi.org/10.15468/dl.3bxkun
Dioprosopa clavatus	United States of America	Texas	Kerr County	29.998239	-99.264299	Occurrence Download https://doi.org/10.15468/dl.3bxkun
Dioprosopa clavatus	United States of America	Arizona	Pima County	32.314863	-110.811287	Occurrence Download https://doi.org/10.15468/dl.3bxkun
Dioprosopa clavatus	Mexico	Puebla	Zapotitlán Salinas	18.32137	-97.48305	Occurrence Download https://doi.org/10.15468/dl.3bxkun
Dioprosopa clavatus	United States of America	Missouri	McDonald County	36.658306	-94.071994	Occurrence Download https://doi.org/10.15468/dl.3bxkun
Dioprosopa clavatus	United States of America	Florida	Polk County	28.25604	-81.595352	Occurrence Download https://doi.org/10.15468/dl.3bxkun
Dioprosopa clavatus	United States of America	California	Rancho San Diego	32.724278	-116.945161	Occurrence Download https://doi.org/10.15468/dl.3bxkun
Dioprosopa clavatus	United States of America	California	Ventura County	34.331365	-118.833801	Occurrence Download https://doi.org/10.15468/dl.3bxkun

Dioprosopa clavatus	United States of America	Arizona	Pima County	32.322561	-110.778096	Occurrence Download https://doi.org/10.15468/dl.3bxkun
Dioprosopa clavatus	United States of America	Texas	Mission	26.176633	-98.365417	Occurrence Download https://doi.org/10.15468/dl.3bxkun
Dioprosopa clavatus	United States of America	Texas	Indigo Lake Estates	30.166762	-95.763113	Occurrence Download https://doi.org/10.15468/dl.3bxkun
Dioprosopa clavatus	United States of America	Florida	Polk County	28.256742	-81.595412	Occurrence Download https://doi.org/10.15468/dl.3bxkun
Dioprosopa clavatus	United States of America	Texas	Mission	26.180042	-98.366792	Occurrence Download https://doi.org/10.15468/dl.3bxkun
Dioprosopa clavatus	United States of America	Texas	Mission	26.179595	-98.365747	Occurrence Download https://doi.org/10.15468/dl.3bxkun
Dioprosopa clavatus	United States of America	Arizona	Tucson	32.214545	-111.05617	Occurrence Download https://doi.org/10.15468/dl.3bxkun
Dioprosopa clavatus	United States of America	Texas	Lockhart	29.883546	-97.687105	Occurrence Download https://doi.org/10.15468/dl.3bxkun
Dioprosopa clavatus	United States of America	California	Santa Barbara County	34.547465	-119.77319	Occurrence Download https://doi.org/10.15468/dl.3bxkun
Dioprosopa clavatus	United States of America	Texas	Aledo	32.683071	-97.604628	Occurrence Download https://doi.org/10.15468/dl.3bxkun
Dioprosopa clavatus	United States of America	Arizona	Chandler	33.28336	-111.842936	Occurrence Download https://doi.org/10.15468/dl.3bxkun
Dioprosopa clavatus	United States of America	Texas	Schertz	29.626208	-98.222137	Occurrence Download https://doi.org/10.15468/dl.3bxkun
Dioprosopa clavatus	United States of America	Georgia	Greene County	33.691988	-83.208954	Occurrence Download https://doi.org/10.15468/dl.3bxkun
Dioprosopa clavatus	United States of America	Arizona	Pima County	32.241294	-110.683792	Occurrence Download https://doi.org/10.15468/dl.3bxkun
Dioprosopa clavatus	Mexico	Guerrero	San Juan Teocalcingo	18.388889	-99.125833	Occurrence Download https://doi.org/10.15468/dl.3bxkun
Dioprosopa clavatus	Brazil	Distrito Federal	Lago Norte	-15.710711	-47.843769	Occurrence Download https://doi.org/10.15468/dl.3bxkun
Dioprosopa clavatus	United States of America	Texas	Mission	26.180447	-98.33076	Occurrence Download https://doi.org/10.15468/dl.3bxkun
Dioprosopa clavatus	United States of America	Arizona	Pinal County	33.185211	-111.656292	Occurrence Download https://doi.org/10.15468/dl.3bxkun
Dioprosopa clavatus	United States of America	Texas	Bacliff	29.505277	-94.977019	Occurrence Download https://doi.org/10.15468/dl.3bxkun
Dioprosopa clavatus	United States of America	Florida	Miami Beach	25.851458	-80.134037	Occurrence Download https://doi.org/10.15468/dl.3bxkun
Dioprosopa clavatus	Brazil	São Paulo	Jaboticabal	-21.238391	-48.342551	Occurrence Download https://doi.org/10.15468/dl.3bxkun
Dioprosopa clavatus	United States of America	Texas	Milam County	30.608061	-97.032051	Occurrence Download https://doi.org/10.15468/dl.3bxkun
Dioprosopa clavatus	United States of America	Texas	Mission	26.180238	-98.330753	Occurrence Download https://doi.org/10.15468/dl.3bxkun
Dioprosopa clavatus	United States of America	Texas	Burnet County	30.5378	-98.17488	Occurrence Download https://doi.org/10.15468/dl.3bxkun
Dioprosopa clavatus	United States of America	California	Fallbrook	33.344147	-117.211267	Occurrence Download https://doi.org/10.15468/dl.3bxkun
Dioprosopa clavatus	United States of America	Louisiana	St. George	30.35752	-91.095354	Occurrence Download https://doi.org/10.15468/dl.3bxkun
Dioprosopa clavatus	United States of America	Texas	San Antonio	29.338933	-98.460375	Occurrence Download https://doi.org/10.15468/dl.3bxkun
Dioprosopa clavatus	Argentina	Buenos Aires	General Villegas	-35.038011	-63.054948	Occurrence Download https://doi.org/10.15468/dl.3bxkun
Dioprosopa clavatus	United States of America	Texas	Mission	26.179025	-98.365914	Occurrence Download https://doi.org/10.15468/dl.3bxkun
Dioprosopa clavatus	Argentina	Santa Fe		-31.550942	-61.067621	Occurrence Download https://doi.org/10.15468/dl.3bxkun
Dioprosopa clavatus	Argentina	Córdoba	Pedanía Los Reartes	-32.001442	-64.77832	Occurrence Download https://doi.org/10.15468/dl.3bxkun
Dioprosopa clavatus	United States of America	Florida	Wedgefield	28.509384	-81.070839	Occurrence Download https://doi.org/10.15468/dl.3bxkun
Dioprosopa clavatus	Argentina	Santa Fe	Municipio de San Guillermo	-30.358972	-61.916885	Occurrence Download https://doi.org/10.15468/dl.3bxkun
Dioprosopa clavatus	Argentina	Córdoba	Bialet Massé	-31.328682	-64.463225	Occurrence Download https://doi.org/10.15468/dl.3bxkun
Dioprosopa clavatus	Mexico	Sonora	San Carlos Nuevo Guaymas	27.989161	-111.081655	Occurrence Download https://doi.org/10.15468/dl.3bxkun
Dioprosopa clavatus	Mexico	Sonora	San Carlos Nuevo Guaymas	27.989077	-111.081653	Occurrence Download https://doi.org/10.15468/dl.3bxkun
Dioprosopa clavatus	Argentina	Córdoba	Capilla del Monte	-30.851921	-64.546112	Occurrence Download https://doi.org/10.15468/dl.3bxkun
Dioprosopa clavatus	Colombia	Caldas	Chinchiná	4.976458	-75.6604	Occurrence Download https://doi.org/10.15468/dl.3bxkun
Dioprosopa clavatus	Colombia	Caldas	Chinchiná	4.971528	-75.649389	Occurrence Download https://doi.org/10.15468/dl.3bxkun

Dioprosopa clavatus	Dominican Republic	Santo Domingo	San Cristóbal	18.525347	-70.089725	Occurrence Download https://doi.org/10.15468/dl.3bxkun
Dioprosopa clavatus	Argentina	Santa Fe	Soldini	-33.017644	-60.749167	Occurrence Download https://doi.org/10.15468/dl.3bxkun
Dioprosopa clavatus	United States of America	California	Willis Palms	33.823378	-116.325917	Occurrence Download https://doi.org/10.15468/dl.3bxkun
Dioprosopa clavatus	Brazil	Mato Grosso	Feliz Natal	-12.382073	-54.936976	Occurrence Download https://doi.org/10.15468/dl.3bxkun
Dioprosopa clavatus	Mexico	Nayarit	Tepic	21.480851	-104.889267	Occurrence Download https://doi.org/10.15468/dl.3bxkun
Dioprosopa clavatus	Brazil	Rio Grande do Sul	Rio Grande	-32.054325	-52.220763	Occurrence Download https://doi.org/10.15468/dl.3bxkun
Dioprosopa clavatus	United States of America	Texas	Austin	30.158735	-97.808244	Occurrence Download https://doi.org/10.15468/dl.3bxkun
Dioprosopa clavatus	United States of America	Florida	Frog City	25.761788	-80.671234	Occurrence Download https://doi.org/10.15468/dl.3bxkun
Dioprosopa clavatus	United States of America	Florida	Collier County	25.857157	-80.921945	Occurrence Download https://doi.org/10.15468/dl.3bxkun
Dioprosopa clavatus	United States of America	Texas	Kendall County	29.807962	-98.764609	Occurrence Download https://doi.org/10.15468/dl.3bxkun
Dioprosopa clavatus	Mexico	Chihuahua	Meoqui	28.272989	-105.471537	Occurrence Download https://doi.org/10.15468/dl.3bxkun
Dioprosopa clavatus	Honduras	Francisco Morazán	San Antonio de Oriente	14.018727	-86.998059	Occurrence Download https://doi.org/10.15468/dl.3bxkun
Dioprosopa clavatus	Costa Rica	Heredia	San Rafael	10.016205	-84.095304	Occurrence Download https://doi.org/10.15468/dl.3bxkun
Dioprosopa clavatus	United States of America	Texas	Boerne	29.793148	-98.736913	Occurrence Download https://doi.org/10.15468/dl.3bxkun
Dioprosopa clavatus	United States of America	Texas	Boerne	29.781688	-98.712516	Occurrence Download https://doi.org/10.15468/dl.3bxkun
Dioprosopa clavatus	Argentina	Córdoba	Oncativo	-31.913429	-63.682063	Occurrence Download https://doi.org/10.15468/dl.3bxkun
Dioprosopa clavatus	United States of America	Oklahoma	Lawton	34.632992	-98.479483	Occurrence Download https://doi.org/10.15468/dl.3bxkun
Dioprosopa clavatus	Mexico	Sinaloa	Téroque Viejo	25.926906	-108.969137	Occurrence Download https://doi.org/10.15468/dl.3bxkun
Dioprosopa clavatus	United States of America	Texas	Brownsboro	32.298997	-95.614358	Occurrence Download https://doi.org/10.15468/dl.3bxkun
Dioprosopa clavatus	United States of America	California	Riverside County	33.809493	-117.265046	Occurrence Download https://doi.org/10.15468/dl.3bxkun
Dioprosopa clavatus	Brazil	Goiás	Aparecida de Goiânia	-16.790509	-49.253866	Occurrence Download https://doi.org/10.15468/dl.3bxkun
Dioprosopa clavatus	United States of America	Florida	Collier County	25.831531	-81.100404	Occurrence Download https://doi.org/10.15468/dl.3bxkun
Dioprosopa clavatus	United States of America	Texas	Comal County	29.879222	-98.204369	Occurrence Download https://doi.org/10.15468/dl.3bxkun
Dioprosopa clavatus	United States of America	Georgia	Skidaway Island	31.956231	-81.02466	Occurrence Download https://doi.org/10.15468/dl.3bxkun
Dioprosopa clavatus	Mexico	Chihuahua	Meoqui	28.273051	-105.471575	Occurrence Download https://doi.org/10.15468/dl.3bxkun
Dioprosopa clavatus	United States of America	Oklahoma	Lawton	34.632916	-98.479531	Occurrence Download https://doi.org/10.15468/dl.3bxkun
Dioprosopa clavatus	United States of America	Oklahoma	Lawton	34.63295	-98.479592	Occurrence Download https://doi.org/10.15468/dl.3bxkun
Dioprosopa clavatus	Mexico	Querétaro	Corregidora	20.522132	-100.387982	Occurrence Download https://doi.org/10.15468/dl.3bxkun
Dioprosopa clavatus	United States of America	Arizona	Yavapai County	34.258981	-112.057756	Occurrence Download https://doi.org/10.15468/dl.3bxkun
Dioprosopa clavatus	United States of America	Texas	Comal County	29.821837	-98.103551	Occurrence Download https://doi.org/10.15468/dl.3bxkun
Dioprosopa clavatus	Argentina	Santa Fe	Soldini	-33.017417	-60.74931	Occurrence Download https://doi.org/10.15468/dl.3bxkun
Dioprosopa clavatus	Mexico	Nuevo León	Guadalupe	25.639288	-100.207153	Occurrence Download https://doi.org/10.15468/dl.3bxkun
Dioprosopa clavatus	United States of America	Texas	Hays County	30.26674	-98.17803	Occurrence Download https://doi.org/10.15468/dl.3bxkun
Dioprosopa clavatus	United States of America	Texas	Frisco	33.121173	-96.840282	Occurrence Download https://doi.org/10.15468/dl.3bxkun
Dioprosopa clavatus	United States of America	California	San Diego County	33.0365	-116.6749	Occurrence Download https://doi.org/10.15468/dl.3bxkun
Dioprosopa clavatus	Cuba	Santiago de Cuba	Santiago de Cuba	20.012662	-75.64726	Occurrence Download https://doi.org/10.15468/dl.3bxkun
Dioprosopa clavatus	United States of America	Texas	Gonzales County	29.483117	-97.449295	Occurrence Download https://doi.org/10.15468/dl.3bxkun
Dioprosopa clavatus	United States of America	Texas	Midland	32.048392	-102.105978	Occurrence Download https://doi.org/10.15468/dl.3bxkun
Dioprosopa clavatus	United States of America	California	Riverside	33.889262	-117.324311	Occurrence Download https://doi.org/10.15468/dl.3bxkun

Dioprosopa clavatus	United States of America	Texas	Azle	32.877983	-97.535697	Occurrence Download https://doi.org/10.15468/dl.3bxkun
Dioprosopa clavatus	United States of America	Texas	Harris County	29.917502	-95.712526	Occurrence Download https://doi.org/10.15468/dl.3bxkun
Dioprosopa clavatus	Guatemala	Jalapa	Jalapa	14.597107	-89.983175	Occurrence Download https://doi.org/10.15468/dl.3bxkun
Dioprosopa clavatus	Dominican Republic	Puerto Plata	Bisonó	19.643548	-70.857473	Occurrence Download https://doi.org/10.15468/dl.3bxkun
Dioprosopa clavatus	Mexico	Guanajuato	Acámbaro	20.040252	-100.723883	Occurrence Download https://doi.org/10.15468/dl.3bxkun
Dioprosopa clavatus	United States of America	Texas	Katy	29.796024	-95.843917	Occurrence Download https://doi.org/10.15468/dl.3bxkun
Dioprosopa clavatus	Mexico	Guerrero	Chilpancingo	17.523243	-99.501374	Occurrence Download https://doi.org/10.15468/dl.3bxkun
Dioprosopa clavatus	Mexico	Nuevo León	Chipinque	25.617726	-100.357032	Occurrence Download https://doi.org/10.15468/dl.3bxkun
Dioprosopa clavatus	United States of America	Georgia	Skidaway Island	31.9564	-81.024732	Occurrence Download https://doi.org/10.15468/dl.3bxkun
Dioprosopa clavatus		Guantanamo Bay USNB	Caimanera	19.922504	-75.12769	Occurrence Download https://doi.org/10.15468/dl.3bxkun
Dioprosopa clavatus	United States of America	Florida	Wedgefield	28.508857	-81.070588	Occurrence Download https://doi.org/10.15468/dl.3bxkun
Dioprosopa clavatus	United States of America	Texas	Allen	33.129818	-96.674896	Occurrence Download https://doi.org/10.15468/dl.3bxkun
Dioprosopa clavatus	United States of America	California	Los Angeles County	34.041342	-118.581445	Occurrence Download https://doi.org/10.15468/dl.3bxkun
Dioprosopa clavatus	United States of America	California	Menifee	33.707669	-117.230384	Occurrence Download https://doi.org/10.15468/dl.3bxkun
Dioprosopa clavatus	Mexico	Aguascalientes	Municipio de Aguascalientes	21.980636	-102.195914	Occurrence Download https://doi.org/10.15468/dl.3bxkun
Dioprosopa clavatus	United States of America	California	Newport Beach	33.604309	-117.796562	Occurrence Download https://doi.org/10.15468/dl.3bxkun
Dioprosopa clavatus	United States of America	California	San Diego County	32.757716	-116.602103	Occurrence Download https://doi.org/10.15468/dl.3bxkun
Dioprosopa clavatus	United States of America	California	San Diego County	32.651938	-116.707122	Occurrence Download https://doi.org/10.15468/dl.3bxkun
Dioprosopa clavatus	United States of America	California	Ventura County	34.377113	-119.024844	Occurrence Download https://doi.org/10.15468/dl.3bxkun
Dioprosopa clavatus	United States of America	North Carolina	Avery County	36.089719	-81.822301	Occurrence Download https://doi.org/10.15468/dl.3bxkun
Dioprosopa clavatus	Argentina	Misiones		-27.039836	-54.134863	Occurrence Download https://doi.org/10.15468/dl.3bxkun
Dioprosopa clavatus	United States of America	Texas	Burkburnett	34.072661	-98.555633	Occurrence Download https://doi.org/10.15468/dl.3bxkun
Dioprosopa clavatus	United States of America	Texas	Sweeny	29.059525	-95.716107	Occurrence Download https://doi.org/10.15468/dl.3bxkun
Dioprosopa clavatus		Florida	Escambia County	30.3	-87.44	Occurrence Download https://doi.org/10.15468/dl.3bxkun
Dioprosopa clavatus	United States of America	Texas	Houston	29.931613	-95.169346	Occurrence Download https://doi.org/10.15468/dl.3bxkun
Dioprosopa clavatus	United States of America	Texas	Sweeny	29.059628	-95.716019	Occurrence Download https://doi.org/10.15468/dl.3bxkun
Dioprosopa clavatus	United States of America	Texas	Montgomery County	30.133094	-95.351244	Occurrence Download https://doi.org/10.15468/dl.3bxkun
Dioprosopa clavatus	Mexico	Chihuahua	Meoqui	28.273114	-105.471578	Occurrence Download https://doi.org/10.15468/dl.3bxkun
Dioprosopa clavatus	United States of America	Texas	Austin	30.20297	-97.70012	Occurrence Download https://doi.org/10.15468/dl.3bxkun
Dioprosopa clavatus	United States of America	Tennessee	Black Fox	35.102702	-84.931873	Occurrence Download https://doi.org/10.15468/dl.3bxkun
Dioprosopa clavatus	United States of America	California	Riverside County	33.756255	-117.346968	Occurrence Download https://doi.org/10.15468/dl.3bxkun
Dioprosopa clavatus	United States of America	California	Jayhawk	38.725782	-120.960938	Occurrence Download https://doi.org/10.15468/dl.3bxkun
Dioprosopa clavatus	United States of America	California	Santa Clara County	37.264216	-121.645084	Occurrence Download https://doi.org/10.15468/dl.3bxkun
Dioprosopa clavatus	United States of America	Texas	Flower Mound	33.023892	-97.145783	Occurrence Download https://doi.org/10.15468/dl.3bxkun
Dioprosopa clavatus	United States of America	Texas	Alvin	29.439676	-95.224905	Occurrence Download https://doi.org/10.15468/dl.3bxkun
Dioprosopa clavatus	United States of America	Texas	Fairview	33.152804	-96.605767	Occurrence Download https://doi.org/10.15468/dl.3bxkun
Dioprosopa clavatus	United States of America	California	Ventura County	34.372737	-118.786488	Occurrence Download https://doi.org/10.15468/dl.3bxkun
Dioprosopa clavatus	United States of America	Florida	Tallahassee	30.484309	-84.217939	Occurrence Download https://doi.org/10.15468/dl.3bxkun
Dioprosopa clavatus	United States of America	California	Unincorporated Chatsworth	34.341052	-118.657664	Occurrence Download https://doi.org/10.15468/dl.3bxkun

Dioprosopa clavatus	United States of America	North Carolina	Cary	35.82367	-78.890975	Occurrence Download https://doi.org/10.15468/dl.3bxkun
Dioprosopa clavatus	United States of America	Florida	Sponge Rocks	25.450195	-80.29395	Occurrence Download https://doi.org/10.15468/dl.3bxkun
Dioprosopa clavatus	United States of America	Mississippi	Pearl	32.234414	-90.101394	Occurrence Download https://doi.org/10.15468/dl.3bxkun
Dioprosopa clavatus	United States of America	Michigan	Albion	42.247624	-84.742993	Occurrence Download https://doi.org/10.15468/dl.3bxkun
Dioprosopa clavatus	United States of America	Missouri	Tallapoosa	36.456636	-89.824209	Occurrence Download https://doi.org/10.15468/dl.3bxkun
Dioprosopa clavatus	United States of America	Texas	Fairview	33.159906	-96.63057	Occurrence Download https://doi.org/10.15468/dl.3bxkun
Dioprosopa clavatus	United States of America	Florida	Gulf Breeze	30.360153	-87.177857	Occurrence Download https://doi.org/10.15468/dl.3bxkun
Dioprosopa clavatus	United States of America	Texas	Ponderosa	31.923102	-102.298612	Occurrence Download https://doi.org/10.15468/dl.3bxkun
Dioprosopa clavatus	United States of America	California	San Jose	37.361902	-121.795659	Occurrence Download https://doi.org/10.15468/dl.3bxkun
Dioprosopa clavatus	Mexico	Querétaro	Corregidora	20.52267	-100.388326	Occurrence Download https://doi.org/10.15468/dl.3bxkun
Dioprosopa clavatus	Dominican Republic	La Romana	La Romana	18.423531	-68.917723	Occurrence Download https://doi.org/10.15468/dl.3bxkun
Dioprosopa clavatus	Mexico	Chihuahua	Meoqui	28.273186	-105.471624	Occurrence Download https://doi.org/10.15468/dl.3bxkun
Dioprosopa clavatus	Mexico	Querétaro	Cadereyta de Montes	20.68462	-99.803477	Occurrence Download https://doi.org/10.15468/dl.3bxkun
Dioprosopa clavatus	Ecuador	Sucumbios	Nueva Loja	0.102279	-76.888324	Occurrence Download https://doi.org/10.15468/dl.3bxkun
Dioprosopa clavatus	Mexico	Chihuahua	Casas Grandes	30.380198	-107.952568	Occurrence Download https://doi.org/10.15468/dl.3bxkun
Dioprosopa clavatus	United States of America	Alabama	Triana	34.606062	-86.745087	Occurrence Download https://doi.org/10.15468/dl.3bxkun
Dioprosopa clavatus	United States of America	California	Laguna Beach	33.540638	-117.781447	Occurrence Download https://doi.org/10.15468/dl.3bxkun
Dioprosopa clavatus	United States of America	Alabama	Triana	34.605975	-86.745064	Occurrence Download https://doi.org/10.15468/dl.3bxkun
Dioprosopa clavatus	United States of America	Delaware	Bayside Hamlet	38.586597	-75.075458	Occurrence Download https://doi.org/10.15468/dl.3bxkun
Dioprosopa clavatus	United States of America	South Carolina	Isle of Palms	32.751003	-79.899036	Occurrence Download https://doi.org/10.15468/dl.3bxkun
Dioprosopa clavatus	United States of America	California	Matilija Hot Springs	34.490047	-119.315497	Occurrence Download https://doi.org/10.15468/dl.3bxkun
Dioprosopa clavatus	United States of America	Texas	Flower Mound	33.043647	-97.181664	Occurrence Download https://doi.org/10.15468/dl.3bxkun
Dioprosopa clavatus	United States of America	Florida	Leon County	30.427792	-84.191565	Occurrence Download https://doi.org/10.15468/dl.3bxkun
Dioprosopa clavatus	United States of America	North Carolina	Chapel Hill	35.89926	-79.033352	Occurrence Download https://doi.org/10.15468/dl.3bxkun
Dioprosopa clavatus	United States of America	Florida	Leon County	30.427914	-84.191624	Occurrence Download https://doi.org/10.15468/dl.3bxkun
Dioprosopa clavatus	United States of America	Mississippi	Choctaw County	33.477733	-89.264992	Occurrence Download https://doi.org/10.15468/dl.3bxkun
Dioprosopa clavatus	United States of America	California	Los Angeles	34.083255	-118.5827	Occurrence Download https://doi.org/10.15468/dl.3bxkun
Dioprosopa clavatus	United States of America	Florida	Collier County	26.061273	-81.404289	Occurrence Download https://doi.org/10.15468/dl.3bxkun
Dioprosopa clavatus	United States of America	California	Ventura County	34.304451	-119.097286	Occurrence Download https://doi.org/10.15468/dl.3bxkun
Dioprosopa clavatus	United States of America	Virginia	Newport News	37.014391	-76.517638	Occurrence Download https://doi.org/10.15468/dl.3bxkun
Dioprosopa clavatus	United States of America	Alabama	Fayette County	33.827719	-87.665742	Occurrence Download https://doi.org/10.15468/dl.3bxkun
Dioprosopa clavatus	United States of America	Georgia	Skidaway Island	31.95619	-81.024568	Occurrence Download https://doi.org/10.15468/dl.3bxkun
Dioprosopa clavatus	United States of America	Oklahoma	Moore	35.300896	-97.423863	Occurrence Download https://doi.org/10.15468/dl.3bxkun
Dioprosopa clavatus	United States of America	Texas	Flower Mound	33.023608	-97.145522	Occurrence Download https://doi.org/10.15468/dl.3bxkun
Dioprosopa clavatus	United States of America	Texas	Sandy Point	29.422165	-95.488093	Occurrence Download https://doi.org/10.15468/dl.3bxkun
Dioprosopa clavatus	Mexico	Tabasco	Centro	17.984645	-92.883181	Occurrence Download https://doi.org/10.15468/dl.3bxkun
Dioprosopa clavatus	Mexico	Chihuahua	Meoqui	28.273116	-105.471596	Occurrence Download https://doi.org/10.15468/dl.3bxkun
Dioprosopa clavatus	Mexico	Michoacán	Jacona de Plancarte	19.934598	-102.310689	Occurrence Download https://doi.org/10.15468/dl.3bxkun
Dioprosopa clavatus	United States of America	Alabama	Franklin County	34.455001	-88.087618	Occurrence Download https://doi.org/10.15468/dl.3bxkun

Dioprosopa clavatus	United States of America	Texas	Hood County	32.365536	-97.707616	Occurrence Download https://doi.org/10.15468/dl.3bxkun
Dioprosopa clavatus	United States of America	California	Piñon Hills	34.410487	-117.625337	Occurrence Download https://doi.org/10.15468/dl.3bxkun
Dioprosopa clavatus	United States of America	California	Piñon Hills	34.408585	-117.62808	Occurrence Download https://doi.org/10.15468/dl.3bxkun
Dioprosopa clavatus	United States of America	Texas	Dickinson	29.462438	-95.049752	Occurrence Download https://doi.org/10.15468/dl.3bxkun
Dioprosopa clavatus	United States of America	Texas	Victoria County	28.795832	-97.052913	Occurrence Download https://doi.org/10.15468/dl.3bxkun
Dioprosopa clavatus	United States of America	North Carolina	Whispering Pines	35.261086	-79.369581	Occurrence Download https://doi.org/10.15468/dl.3bxkun
Dioprosopa clavatus	United States of America	Virginia	Orange County	38.218824	-78.168457	Occurrence Download https://doi.org/10.15468/dl.3bxkun
Dioprosopa clavatus	United States of America	South Carolina	James Island	32.719973	-79.95354	Occurrence Download https://doi.org/10.15468/dl.3bxkun
Dioprosopa clavatus	United States of America	Texas	Waco	31.487188	-97.287768	Occurrence Download https://doi.org/10.15468/dl.3bxkun
Dioprosopa clavatus	United States of America	Florida	Wedgefield	28.486314	-81.09755	Occurrence Download https://doi.org/10.15468/dl.3bxkun
Dioprosopa clavatus	United States of America	Georgia	Skidaway Island	31.956277	-81.024681	Occurrence Download https://doi.org/10.15468/dl.3bxkun
Dioprosopa clavatus	United States of America	Georgia	Skidaway Island	31.956732	-81.024474	Occurrence Download https://doi.org/10.15468/dl.3bxkun
Dioprosopa clavatus	United States of America	South Carolina	Charleston	32.730081	-79.961087	Occurrence Download https://doi.org/10.15468/dl.3bxkun
Dioprosopa clavatus	United States of America	Texas	Brownsville	26.128497	-97.524724	Occurrence Download https://doi.org/10.15468/dl.3bxkun
Dioprosopa clavatus	United States of America	Florida	Punta Gorda	26.874316	-82.027664	Occurrence Download https://doi.org/10.15468/dl.3bxkun
Dioprosopa clavatus	United States of America	Texas	Mansfield	32.601409	-97.157338	Occurrence Download https://doi.org/10.15468/dl.3bxkun
Dioprosopa clavatus	United States of America	Texas	Austin	30.227453	-97.653626	Occurrence Download https://doi.org/10.15468/dl.3bxkun
Dioprosopa clavatus	United States of America	South Carolina	Charleston	32.724017	-79.954908	Occurrence Download https://doi.org/10.15468/dl.3bxkun
Dioprosopa clavatus	United States of America	Texas	Cinco Ranch	29.738915	-95.760716	Occurrence Download https://doi.org/10.15468/dl.3bxkun
Dioprosopa clavatus	United States of America	California	La Quinta	33.672186	-116.255808	Occurrence Download https://doi.org/10.15468/dl.3bxkun
Dioprosopa clavatus	United States of America	Iowa	La Porte City	42.308312	-92.191738	Occurrence Download https://doi.org/10.15468/dl.3bxkun
Dioprosopa clavatus	United States of America	California	Fairfield	38.283227	-122.029072	Occurrence Download https://doi.org/10.15468/dl.3bxkun
Dioprosopa clavatus	United States of America	North Carolina	Davidson	35.497287	-80.85287	Occurrence Download https://doi.org/10.15468/dl.3bxkun
Dioprosopa clavatus	United States of America	South Carolina	Charleston	32.973828	-79.771025	Occurrence Download https://doi.org/10.15468/dl.3bxkun
Dioprosopa clavatus	United States of America	Texas	Coryell County	31.332042	-97.469365	Occurrence Download https://doi.org/10.15468/dl.3bxkun
Dioprosopa clavatus	United States of America	Indiana	Fields	39.511681	-86.337676	Occurrence Download https://doi.org/10.15468/dl.3bxkun
Dioprosopa clavatus	United States of America	Georgia	Skidaway Island	31.956222	-81.02463	Occurrence Download https://doi.org/10.15468/dl.3bxkun
Dioprosopa clavatus	United States of America	Texas	Ward	31.914478	-95.796863	Occurrence Download https://doi.org/10.15468/dl.3bxkun
Dioprosopa clavatus	United States of America	Indiana	Hendricks County	39.848916	-86.329714	Occurrence Download https://doi.org/10.15468/dl.3bxkun
Dioprosopa clavatus	United States of America	Texas	Corpus Christi	27.648966	-97.39354	Occurrence Download https://doi.org/10.15468/dl.3bxkun
Dioprosopa clavatus	United States of America	Texas	Uvalde County	29.567871	-99.723658	Occurrence Download https://doi.org/10.15468/dl.3bxkun
Dioprosopa clavatus	United States of America	Texas	Starr County	26.584631	-99.149567	Occurrence Download https://doi.org/10.15468/dl.3bxkun
Dioprosopa clavatus	United States of America	Texas	Kerrville	29.94656	-99.237579	Occurrence Download https://doi.org/10.15468/dl.3bxkun
Dioprosopa clavatus	United States of America	Texas	Fort Bend County	29.507227	-95.44471	Occurrence Download https://doi.org/10.15468/dl.3bxkun
Dioprosopa clavatus	United States of America	Florida	Orange County	28.570974	-80.996351	Occurrence Download https://doi.org/10.15468/dl.3bxkun
Dioprosopa clavatus	Mexico	Chihuahua	Meoqui	28.273069	-105.471577	Occurrence Download https://doi.org/10.15468/dl.3bxkun
Dioprosopa clavatus	United States of America	South Carolina	Charleston	32.728637	-79.957652	Occurrence Download https://doi.org/10.15468/dl.3bxkun
Dioprosopa clavatus	Brazil	Rio Grande do Sul	Taquari	-29.800881	-51.845347	Occurrence Download https://doi.org/10.15468/dl.3bxkun
Dioprosopa clavatus	United States of America	Florida	Miami-Dade County	25.805662	-80.853065	Occurrence Download https://doi.org/10.15468/dl.3bxkun

Dioprosopa clavatus	Brazil	Rio Grande do Sul	Maquiné	-29.771263	-50.085678	Occurrence Download https://doi.org/10.15468/dl.3bxkun
Dioprosopa clavatus	Colombia	Santander	Floridablanca	7.099722	-73.067222	Occurrence Download https://doi.org/10.15468/dl.3bxkun
Dioprosopa clavatus	United States of America	Texas	Lubbock	33.56441	-101.973913	Occurrence Download https://doi.org/10.15468/dl.3bxkun
Dioprosopa clavatus	United States of America	Florida	Homestead	25.753847	-80.497814	Occurrence Download https://doi.org/10.15468/dl.3bxkun
Dioprosopa clavatus	Colombia	Caldas	Chinchiná	4.970694	-75.660222	Occurrence Download https://doi.org/10.15468/dl.3bxkun
Dioprosopa clavatus	Colombia	Quindío	Buenavista	4.397661	-75.735769	Occurrence Download https://doi.org/10.15468/dl.3bxkun
Dioprosopa clavatus	United States of America	Florida	Homestead	25.759133	-80.498005	Occurrence Download https://doi.org/10.15468/dl.3bxkun
Dioprosopa clavatus	United States of America	Texas	Cedar Park	30.513256	-97.755366	Occurrence Download https://doi.org/10.15468/dl.3bxkun
Dioprosopa clavatus	United States of America	Arizona	Pima County	32.313033	-110.741853	Occurrence Download https://doi.org/10.15468/dl.3bxkun
Dioprosopa clavatus	United States of America	Texas	Presidio County	29.483069	-103.973297	Occurrence Download https://doi.org/10.15468/dl.3bxkun
Dioprosopa clavatus		California	Moss Beach	37.529492	-122.516617	Occurrence Download https://doi.org/10.15468/dl.3bxkun
Dioprosopa clavatus	United States of America	California	San Diego County	32.734522	-116.871381	Occurrence Download https://doi.org/10.15468/dl.3bxkun
Dioprosopa clavatus	United States of America	Florida	Wedgefield	28.510099	-81.070715	Occurrence Download https://doi.org/10.15468/dl.3bxkun
Dioprosopa clavatus	United States of America	Florida	Wedgefield	28.510108	-81.070706	Occurrence Download https://doi.org/10.15468/dl.3bxkun
Dioprosopa clavatus	United States of America	Florida	Homestead	25.750058	-80.497842	Occurrence Download https://doi.org/10.15468/dl.3bxkun
Dioprosopa clavatus	United States of America	Florida	Homestead	25.758614	-80.49823	Occurrence Download https://doi.org/10.15468/dl.3bxkun
Dioprosopa clavatus	Mexico	Sinaloa	Culiacán	24.759594	-107.465083	Occurrence Download https://doi.org/10.15468/dl.3bxkun
Dioprosopa clavatus	United States of America	California	Los Angeles	34.045675	-118.558835	Occurrence Download https://doi.org/10.15468/dl.3bxkun
Dioprosopa clavatus	United States of America	Texas	Maypearl	32.3118	-97.015895	Occurrence Download https://doi.org/10.15468/dl.3bxkun
Dioprosopa clavatus	United States of America	Texas	Grimes County	30.300488	-95.804108	Occurrence Download https://doi.org/10.15468/dl.3bxkun
Dioprosopa clavatus	United States of America	Texas	Blanco	30.103569	-98.42593	Occurrence Download https://doi.org/10.15468/dl.3bxkun
Dioprosopa clavatus	United States of America	New York	Town of Bedford	41.221099	-73.643333	Occurrence Download https://doi.org/10.15468/dl.3bxkun
Dioprosopa clavatus	United States of America	Texas	Vilas	30.838275	-97.304875	Occurrence Download https://doi.org/10.15468/dl.3bxkun
Dioprosopa clavatus	United States of America	Texas	Abilene	32.466928	-99.701652	Occurrence Download https://doi.org/10.15468/dl.3bxkun
Dioprosopa clavatus	United States of America	Texas	Denton	33.146439	-97.096077	Occurrence Download https://doi.org/10.15468/dl.3bxkun
Dioprosopa clavatus	United States of America	Florida	Wedgefield	28.510074	-81.070703	Occurrence Download https://doi.org/10.15468/dl.3bxkun
Dioprosopa clavatus	United States of America	Texas	Tuscan Oaks	29.692411	-98.445821	Occurrence Download https://doi.org/10.15468/dl.3bxkun
Dioprosopa clavatus	United States of America	Florida	Orange County	28.57514	-80.996529	Occurrence Download https://doi.org/10.15468/dl.3bxkun
Dioprosopa clavatus	United States of America	Texas	Erath County	32.164851	-98.286781	Occurrence Download https://doi.org/10.15468/dl.3bxkun
Dioprosopa clavatus	United States of America	Florida	Wedgefield	28.509983	-81.07077	Occurrence Download https://doi.org/10.15468/dl.3bxkun
Dioprosopa clavatus	United States of America	Texas	Fort Bend County	29.577625	-95.433058	Occurrence Download https://doi.org/10.15468/dl.3bxkun
Dioprosopa clavatus	United States of America	Florida	Pinecrest	25.761103	-80.922997	Occurrence Download https://doi.org/10.15468/dl.3bxkun
Dioprosopa clavatus	United States of America	Nebraska	Nebraska City	40.697419	-95.893467	Occurrence Download https://doi.org/10.15468/dl.3bxkun
Dioprosopa clavatus	United States of America	New Jersey	Bridgewater Township	40.609233	-74.573616	Occurrence Download https://doi.org/10.15468/dl.3bxkun
Dioprosopa clavatus	United States of America	Texas	Indigo Lake Estates	30.166599	-95.763125	Occurrence Download https://doi.org/10.15468/dl.3bxkun
Dioprosopa clavatus	Brazil	São Paulo	Brodowski	-20.988297	-47.657974	Occurrence Download https://doi.org/10.15468/dl.3bxkun
Dioprosopa clavatus	Mexico	Michoacán	Jacona de Plancarte	19.938767	-102.298776	Occurrence Download https://doi.org/10.15468/dl.3bxkun
Dioprosopa clavatus	United States of America	Texas	Maypearl	32.311809	-97.015954	Occurrence Download https://doi.org/10.15468/dl.3bxkun
Dioprosopa clavatus	United States of America	Florida	Wedgefield	28.509304	-81.070831	Occurrence Download https://doi.org/10.15468/dl.3bxkun

Dioprosopa clavatus	United States of America	Texas	Maypearl	32.311782	-97.015959	Occurrence Download https://doi.org/10.15468/dl.3bxkun
Dioprosopa clavatus	United States of America	Texas	Maypearl	32.311777	-97.01597	Occurrence Download https://doi.org/10.15468/dl.3bxkun
Dioprosopa clavatus	United States of America	Florida	Orlando	28.559845	-81.129112	Occurrence Download https://doi.org/10.15468/dl.3bxkun
Dioprosopa clavatus	United States of America	Texas	Tool	32.290431	-96.169819	Occurrence Download https://doi.org/10.15468/dl.3bxkun
Dioprosopa clavatus	United States of America	Oklahoma	Norman	35.253245	-97.435921	Occurrence Download https://doi.org/10.15468/dl.3bxkun
Dioprosopa clavatus	United States of America	Florida	Wedgefield	28.508793	-81.071167	Occurrence Download https://doi.org/10.15468/dl.3bxkun
Dioprosopa clavatus	United States of America	Texas	San Marcos	29.906862	-97.895767	Occurrence Download https://doi.org/10.15468/dl.3bxkun
Dioprosopa clavatus	Dominican Republic	La Altagracia	Punta Cana	18.556148	-68.377424	Occurrence Download https://doi.org/10.15468/dl.3bxkun
Dioprosopa clavatus	United States of America	Illinois	Lakeview Acres	38.714374	-89.942861	Occurrence Download https://doi.org/10.15468/dl.3bxkun
Dioprosopa clavatus	United States of America	Alabama	Blountsville	34.109546	-86.621359	Occurrence Download https://doi.org/10.15468/dl.3bxkun
Dioprosopa clavatus	United States of America	Georgia	Chattahoochee Hills	33.516225	-84.740333	Occurrence Download https://doi.org/10.15468/dl.3bxkun
Dioprosopa clavatus	United States of America	Texas	Lubbock County	33.597357	-101.96105	Occurrence Download https://doi.org/10.15468/dl.3bxkun
Dioprosopa clavatus	United States of America	Texas	Flower Mound	33.047861	-97.164475	Occurrence Download https://doi.org/10.15468/dl.3bxkun
Dioprosopa clavatus	United States of America	Florida	Poinciana	28.121408	-81.44133	Occurrence Download https://doi.org/10.15468/dl.3bxkun
Dioprosopa clavatus	United States of America	Texas	Maypearl	32.311759	-97.015965	Occurrence Download https://doi.org/10.15468/dl.3bxkun
Dioprosopa clavatus	United States of America	California	Los Angeles County	34.552187	-118.515495	Occurrence Download https://doi.org/10.15468/dl.3bxkun
Dioprosopa clavatus	United States of America	Florida	Poinciana	28.141439	-81.4459	Occurrence Download https://doi.org/10.15468/dl.3bxkun
Dioprosopa clavatus	United States of America	Texas	Georgetown	30.616898	-97.611442	Occurrence Download https://doi.org/10.15468/dl.3bxkun
Dioprosopa clavatus	United States of America	South Carolina	James Island	32.725984	-79.95844	Occurrence Download https://doi.org/10.15468/dl.3bxkun
Dioprosopa clavatus	United States of America	Texas	Ellis County	32.294756	-96.690191	Occurrence Download https://doi.org/10.15468/dl.3bxkun
Dioprosopa clavatus	United States of America	Florida	Suwannee County	30.023901	-82.869704	Occurrence Download https://doi.org/10.15468/dl.3bxkun
Dioprosopa clavatus	United States of America	Florida	Wedgefield	28.510244	-81.06395	Occurrence Download https://doi.org/10.15468/dl.3bxkun
Dioprosopa clavatus	United States of America	Florida	Wedgefield	28.510086	-81.064125	Occurrence Download https://doi.org/10.15468/dl.3bxkun
Dioprosopa clavatus	United States of America	Tamaulipas	La Brigada	26.01894	-97.641844	Occurrence Download https://doi.org/10.15468/dl.3bxkun
Dioprosopa clavatus	United States of America	Texas	Los Indios	26.069702	-97.769604	Occurrence Download https://doi.org/10.15468/dl.3bxkun
Dioprosopa clavatus	United States of America	Texas	Rangerville	26.117576	-97.751502	Occurrence Download https://doi.org/10.15468/dl.3bxkun
Dioprosopa clavatus	United States of America	Texas	Saint Hedwig	29.432334	-98.226229	Occurrence Download https://doi.org/10.15468/dl.3bxkun
Dioprosopa clavatus	United States of America	Florida	Suwannee County	30.02459	-82.869639	Occurrence Download https://doi.org/10.15468/dl.3bxkun
Dioprosopa clavatus	United States of America	Georgia	Pierce County	31.307642	-82.349767	Occurrence Download https://doi.org/10.15468/dl.3bxkun
Dioprosopa clavatus	Bolivia	La Paz	Municipio Coroico	-16.194895	-67.741699	Occurrence Download https://doi.org/10.15468/dl.3bxkun
Dioprosopa clavatus	United States of America	Florida	Wedgefield	28.508539	-81.071068	Occurrence Download https://doi.org/10.15468/dl.3bxkun
Dioprosopa clavatus	United States of America	Florida	Wedgefield	28.509319	-81.070839	Occurrence Download https://doi.org/10.15468/dl.3bxkun
Dioprosopa clavatus	United States of America	Texas	Indigo Lake Estates	30.166516	-95.763109	Occurrence Download https://doi.org/10.15468/dl.3bxkun
Dioprosopa clavatus	United States of America	Florida	Wedgefield	28.50935	-81.070816	Occurrence Download https://doi.org/10.15468/dl.3bxkun
Dioprosopa clavatus	United States of America	South Carolina	Charleston	32.7321	-79.957306	Occurrence Download https://doi.org/10.15468/dl.3bxkun
Dioprosopa clavatus	United States of America	South Carolina	James Island	32.727949	-79.958275	Occurrence Download https://doi.org/10.15468/dl.3bxkun
Dioprosopa clavatus	United States of America	Texas	Mission	26.179503	-98.366337	Occurrence Download https://doi.org/10.15468/dl.3bxkun
Dioprosopa clavatus	United States of America	Texas	Schertz	29.476476	-98.268589	Occurrence Download https://doi.org/10.15468/dl.3bxkun
Dioprosopa clavatus	Mexico	Chihuahua	Meoqui	28.273056	-105.471615	Occurrence Download https://doi.org/10.15468/dl.3bxkun

Dioprosopa clavatus	Mexico	Chihuahua	Meoqui	28.273107	-105.471576	Occurrence Download https://doi.org/10.15468/dl.3bxkun
Dioprosopa clavatus	Mexico	Querétaro	Ezequiel Montes	20.754719	-99.952669	Occurrence Download https://doi.org/10.15468/dl.3bxkun
Dioprosopa clavatus	Mexico	Chihuahua	Janos	30.891422	-108.189652	Occurrence Download https://doi.org/10.15468/dl.3bxkun
Dioprosopa clavatus	United States of America	California	Temecula	33.535167	-117.111017	Occurrence Download https://doi.org/10.15468/dl.3bxkun
Dioprosopa clavatus	United States of America	Maryland	Frederick County	39.475708	-77.269981	Occurrence Download https://doi.org/10.15468/dl.3bxkun
Dioprosopa clavatus	United States of America	Florida	Wedgefield	28.509314	-81.070808	Occurrence Download https://doi.org/10.15468/dl.3bxkun
Dioprosopa clavatus	United States of America	Texas	Mission	26.176354	-98.366495	Occurrence Download https://doi.org/10.15468/dl.3bxkun
Dioprosopa clavatus	United States of America	Florida	Wedgefield	28.509275	-81.070793	Occurrence Download https://doi.org/10.15468/dl.3bxkun
Dioprosopa clavatus	United States of America	Florida	Collier County	25.857187	-81.033279	Occurrence Download https://doi.org/10.15468/dl.3bxkun
Dioprosopa clavatus	Mexico	Querétaro	Corregidora	20.522632	-100.388412	Occurrence Download https://doi.org/10.15468/dl.3bxkun
Dioprosopa clavatus	United States of America	Florida	Orange County	28.359021	-81.205615	Occurrence Download https://doi.org/10.15468/dl.3bxkun
Dioprosopa clavatus	Paraguay	Cordillera		-25.367347	-57.166527	Occurrence Download https://doi.org/10.15468/dl.3bxkun
Dioprosopa clavatus	Brazil	Rio Grande do Sul	Rio Grande	-32.041916	-52.256006	Occurrence Download https://doi.org/10.15468/dl.3bxkun
Dioprosopa clavatus	United States of America	Texas	Maypearl	32.311411	-97.014929	Occurrence Download https://doi.org/10.15468/dl.3bxkun
Dioprosopa clavatus	United States of America	Texas	Paloma Lake	30.562927	-97.621465	Occurrence Download https://doi.org/10.15468/dl.3bxkun
Dioprosopa clavatus	United States of America	Texas	Fort Worth	32.625308	-97.426996	Occurrence Download https://doi.org/10.15468/dl.3bxkun
Dioprosopa clavatus	United States of America	Florida	Enterprise	28.861208	-81.28426	Occurrence Download https://doi.org/10.15468/dl.3bxkun
Dioprosopa clavatus	United States of America	Florida	DeBary	28.865306	-81.283337	Occurrence Download https://doi.org/10.15468/dl.3bxkun
Dioprosopa clavatus	United States of America	Florida	DeBary	28.863912	-81.283718	Occurrence Download https://doi.org/10.15468/dl.3bxkun
Dioprosopa clavatus	United States of America	Texas	Lake View	29.461202	-100.9745	Occurrence Download https://doi.org/10.15468/dl.3bxkun
Dioprosopa clavatus	United States of America	California	San Diego County	32.937626	-116.963013	Occurrence Download https://doi.org/10.15468/dl.3bxkun
Dioprosopa clavatus	United States of America	Arizona	Scottsdale	33.554552	-111.875756	Occurrence Download https://doi.org/10.15468/dl.3bxkun
Dioprosopa clavatus	United States of America	Florida	Sarasota County	27.256235	-82.478078	Occurrence Download https://doi.org/10.15468/dl.3bxkun
Dioprosopa clavatus	United States of America	Arizona	Gila County	34.048928	-111.093731	Occurrence Download https://doi.org/10.15468/dl.3bxkun
Dioprosopa clavatus	United States of America	Louisiana	Vermilion Parish	30.008226	-92.048462	Occurrence Download https://doi.org/10.15468/dl.3bxkun
Dioprosopa clavatus	Mexico	Nuevo León	García	25.767275	-100.435441	Occurrence Download https://doi.org/10.15468/dl.3bxkun
Dioprosopa clavatus	Brazil	Paraíba	Conceição	-7.529662	-38.4947	Occurrence Download https://doi.org/10.15468/dl.3bxkun
Dioprosopa clavatus	United States of America	Florida	Wedgefield	28.510874	-81.070218	Occurrence Download https://doi.org/10.15468/dl.3bxkun
Dioprosopa clavatus	Cuba	Guantanamo Bay USNB	Caimanera	19.916573	-75.144085	Occurrence Download https://doi.org/10.15468/dl.3bxkun
Dioprosopa clavatus	Cuba	Boqueron	Caimanera	19.918122	-75.163797	Occurrence Download https://doi.org/10.15468/dl.3bxkun
Dioprosopa clavatus	United States of America	Illinois	Kelsey	40.224479	-90.048965	Occurrence Download https://doi.org/10.15468/dl.3bxkun
Dioprosopa clavatus	United States of America	Texas	Austin	30.172067	-97.997955	Occurrence Download https://doi.org/10.15468/dl.3bxkun
Dioprosopa clavatus	United States of America	Florida	Wedgefield	28.510028	-81.070722	Occurrence Download https://doi.org/10.15468/dl.3bxkun
Dioprosopa clavatus	United States of America	Texas	Dunlap	30.26037	-97.544603	Occurrence Download https://doi.org/10.15468/dl.3bxkun
Dioprosopa clavatus	United States of America	Arizona	Santa Cruz County	31.526722	-110.772622	Occurrence Download https://doi.org/10.15468/dl.3bxkun
Dioprosopa clavatus	United States of America	Texas	Travis County	30.382765	-97.494396	Occurrence Download https://doi.org/10.15468/dl.3bxkun
Dioprosopa clavatus	United States of America	Texas	Travis County	30.26874	-97.471701	Occurrence Download https://doi.org/10.15468/dl.3bxkun
Dioprosopa clavatus	United States of America	Texas	Travis County	30.340749	-97.424031	Occurrence Download https://doi.org/10.15468/dl.3bxkun
Dioprosopa clavatus	United States of America	Texas	Youngs Prairie	30.297321	-97.40931	Occurrence Download https://doi.org/10.15468/dl.3bxkun

Dioprosopa clavatus	United States of America	Texas	Cinco Ranch	29.741244	-95.753434	Occurrence Download https://doi.org/10.15468/dl.3bxkun
Dioprosopa clavatus	United States of America	California	Forest Falls	34.088345	-116.920305	Occurrence Download https://doi.org/10.15468/dl.3bxkun
Dioprosopa clavatus	United States of America	Florida	Broward County	26.338865	-80.628129	Occurrence Download https://doi.org/10.15468/dl.3bxkun
Dioprosopa clavatus	United States of America	Florida	Wedgefield	28.510869	-81.070167	Occurrence Download https://doi.org/10.15468/dl.3bxkun
Dioprosopa clavatus	United States of America	Arkansas	Barling	35.325647	-94.301601	Occurrence Download https://doi.org/10.15468/dl.3bxkun
Dioprosopa clavatus	United States of America	Florida	Wedgefield	28.509273	-81.070545	Occurrence Download https://doi.org/10.15468/dl.3bxkun
Dioprosopa clavatus	United States of America	Oklahoma	Rogers County	36.293346	-95.653458	Occurrence Download https://doi.org/10.15468/dl.3bxkun
Dioprosopa clavatus	United States of America	Florida	Marion County	29.080828	-82.276497	Occurrence Download https://doi.org/10.15468/dl.3bxkun
Dioprosopa clavatus	United States of America	New Mexico	Carlsbad	32.368332	-104.263336	Occurrence Download https://doi.org/10.15468/dl.3bxkun
Dioprosopa clavatus	United States of America	Oklahoma	Woodward County	36.543166	-99.554419	Occurrence Download https://doi.org/10.15468/dl.3bxkun
Dioprosopa clavatus	United States of America	Texas	Combine	32.581194	-96.514709	Occurrence Download https://doi.org/10.15468/dl.3bxkun
Dioprosopa clavatus	United States of America	Arkansas	Craighead County	35.742146	-90.668908	Occurrence Download https://doi.org/10.15468/dl.3bxkun
Dioprosopa clavatus	United States of America	Oklahoma	Edmond	35.717571	-97.429222	Occurrence Download https://doi.org/10.15468/dl.3bxkun
Dioprosopa clavatus	United States of America	Texas	Paloma Lake	30.563178	-97.621783	Occurrence Download https://doi.org/10.15468/dl.3bxkun
Dioprosopa clavatus	United States of America	Texas	Burleson	32.530578	-97.321403	Occurrence Download https://doi.org/10.15468/dl.3bxkun
Dioprosopa clavatus	United States of America	Texas	Austin	30.337312	-97.89447	Occurrence Download https://doi.org/10.15468/dl.3bxkun
Dioprosopa clavatus	Cuba	Boqueron	Caimanera	19.912162	-75.164731	Occurrence Download https://doi.org/10.15468/dl.3bxkun
Dioprosopa clavatus	United States of America	Arkansas	Bunney	35.72457	-90.419106	Occurrence Download https://doi.org/10.15468/dl.3bxkun
Dioprosopa clavatus	United States of America	Oklahoma	Altus	34.661662	-99.340675	Occurrence Download https://doi.org/10.15468/dl.3bxkun
Dioprosopa clavatus	United States of America	Arizona	Pima County	32.24302	-111.169128	Occurrence Download https://doi.org/10.15468/dl.3bxkun
Dioprosopa clavatus	United States of America	Texas	Vilas	30.837816	-97.304753	Occurrence Download https://doi.org/10.15468/dl.3bxkun
Dioprosopa clavatus	Mexico	Texas	Hidalgo County	26.077514	-98.137295	Occurrence Download https://doi.org/10.15468/dl.3bxkun
Dioprosopa clavatus	United States of America	Texas	Doffing	26.344828	-98.390124	Occurrence Download https://doi.org/10.15468/dl.3bxkun
Dioprosopa clavatus	United States of America	Oklahoma	Woodward	36.437985	-99.419577	Occurrence Download https://doi.org/10.15468/dl.3bxkun
Dioprosopa clavatus	United States of America	Texas	Keller	32.963168	-97.232642	Occurrence Download https://doi.org/10.15468/dl.3bxkun
Dioprosopa clavatus	United States of America	South Carolina	Charleston County	32.681105	-80.34324	Occurrence Download https://doi.org/10.15468/dl.3bxkun
Dioprosopa clavatus	United States of America	Texas	Johnson County	32.216765	-97.607348	Occurrence Download https://doi.org/10.15468/dl.3bxkun
Dioprosopa clavatus	United States of America	Texas	Keller	32.9631	-97.232633	Occurrence Download https://doi.org/10.15468/dl.3bxkun
Dioprosopa clavatus	United States of America	Texas	Granbury	32.508367	-97.729122	Occurrence Download https://doi.org/10.15468/dl.3bxkun
Dioprosopa clavatus	United States of America	California	Los Angeles	33.72	-118.32	Occurrence Download https://doi.org/10.15468/dl.3bxkun
Dioprosopa clavatus	United States of America	California	Los Angeles	33.72	-118.31	Occurrence Download https://doi.org/10.15468/dl.3bxkun
Dioprosopa clavatus	United States of America	New York	City of New York	40.783615	-73.918543	Occurrence Download https://doi.org/10.15468/dl.3bxkun
Dioprosopa clavatus	United States of America	Texas	Bastrop County	30.136667	-97.44562	Occurrence Download https://doi.org/10.15468/dl.3bxkun
Dioprosopa clavatus	United States of America	Texas	Lubbock	33.526962	-101.960134	Occurrence Download https://doi.org/10.15468/dl.3bxkun
Dioprosopa clavatus	United States of America	Texas	Maypearl	32.311778	-97.016066	Occurrence Download https://doi.org/10.15468/dl.3bxkun
Dioprosopa clavatus	United States of America	Texas	Flower Mound	33.023525	-97.145728	Occurrence Download https://doi.org/10.15468/dl.3bxkun
Dioprosopa clavatus	United States of America	Texas	Travis County	30.375839	-97.911383	Occurrence Download https://doi.org/10.15468/dl.3bxkun
Dioprosopa clavatus	United States of America	Texas	Mesquite	32.733642	-96.586322	Occurrence Download https://doi.org/10.15468/dl.3bxkun
Dioprosopa clavatus	United States of America	Texas	Manor	30.368492	-97.626625	Occurrence Download https://doi.org/10.15468/dl.3bxkun

Dioprosopa clavatus	United States of America	Texas	Bastrop County	29.988609	-97.293557	Occurrence Download https://doi.org/10.15468/dl.3bxkun
Dioprosopa clavatus	United States of America	Texas	Austin	30.194908	-97.61209	Occurrence Download https://doi.org/10.15468/dl.3bxkun
Dioprosopa clavatus	United States of America	Texas	Azle	32.890323	-97.559638	Occurrence Download https://doi.org/10.15468/dl.3bxkun
Dioprosopa clavatus	United States of America	Texas	Leander	30.589645	-97.874213	Occurrence Download https://doi.org/10.15468/dl.3bxkun
Dioprosopa clavatus	United States of America	Texas	Los Castillos Estates Colonia	26.12405	-97.952623	Occurrence Download https://doi.org/10.15468/dl.3bxkun
Dioprosopa clavatus	United States of America	Arizona	Pima County	32.153472	-110.608697	Occurrence Download https://doi.org/10.15468/dl.3bxkun
Dioprosopa clavatus	United States of America	Texas	Johnson City	30.274385	-98.417826	Occurrence Download https://doi.org/10.15468/dl.3bxkun
Dioprosopa clavatus	United States of America	Texas	Llano County	30.502263	-98.820105	Occurrence Download https://doi.org/10.15468/dl.3bxkun
Dioprosopa clavatus	United States of America	Texas	Austin	30.185446	-97.873211	Occurrence Download https://doi.org/10.15468/dl.3bxkun
Dioprosopa clavatus	United States of America	Texas	Bandera County	29.734288	-99.012414	Occurrence Download https://doi.org/10.15468/dl.3bxkun
Dioprosopa clavatus	United States of America	Texas	Palmhurst	26.269903	-98.338157	Occurrence Download https://doi.org/10.15468/dl.3bxkun
Dioprosopa clavatus	United States of America	Texas	Wise County	33.319517	-97.622779	Occurrence Download https://doi.org/10.15468/dl.3bxkun
Dioprosopa clavatus	United States of America	Texas	Georgetown	30.668302	-97.722145	Occurrence Download https://doi.org/10.15468/dl.3bxkun
Dioprosopa clavatus	United States of America	Texas	Vilas	30.838139	-97.304916	Occurrence Download https://doi.org/10.15468/dl.3bxkun
Dioprosopa clavatus	United States of America	Texas	Austin	30.161994	-97.844264	Occurrence Download https://doi.org/10.15468/dl.3bxkun
Dioprosopa clavatus	United States of America	Texas	Bastrop County	30.143032	-97.45822	Occurrence Download https://doi.org/10.15468/dl.3bxkun
Dioprosopa clavatus	United States of America	Texas	Georgetown	30.693184	-97.657216	Occurrence Download https://doi.org/10.15468/dl.3bxkun
Dioprosopa clavatus	United States of America	Texas	Blanco	30.103661	-98.425918	Occurrence Download https://doi.org/10.15468/dl.3bxkun
Dioprosopa clavatus	United States of America	Texas	Wise County	33.340128	-97.59682	Occurrence Download https://doi.org/10.15468/dl.3bxkun
Dioprosopa clavatus	United States of America	Texas	Powell	31.124065	-94.079602	Occurrence Download https://doi.org/10.15468/dl.3bxkun
Dioprosopa clavatus	United States of America	Texas	Citrus City	26.336419	-98.381945	Occurrence Download https://doi.org/10.15468/dl.3bxkun
Dioprosopa clavatus	United States of America	Texas	Bandera County	29.734367	-99.012458	Occurrence Download https://doi.org/10.15468/dl.3bxkun
Dioprosopa clavatus	United States of America	Texas	Gun Barrel City	32.336314	-96.117148	Occurrence Download https://doi.org/10.15468/dl.3bxkun
Dioprosopa clavatus	United States of America	Texas	Blanco	30.103768	-98.425979	Occurrence Download https://doi.org/10.15468/dl.3bxkun
Dioprosopa clavatus	United States of America	Texas	Austin	30.184735	-97.873578	Occurrence Download https://doi.org/10.15468/dl.3bxkun
Dioprosopa clavatus	United States of America	Texas	Blanco County	30.269567	-98.566669	Occurrence Download https://doi.org/10.15468/dl.3bxkun
Dioprosopa clavatus	United States of America	Texas	Johnson County	32.442625	-97.606569	Occurrence Download https://doi.org/10.15468/dl.3bxkun
Dioprosopa clavatus	United States of America	Texas	Blanco County	30.132069	-98.373349	Occurrence Download https://doi.org/10.15468/dl.3bxkun
Dioprosopa clavatus	United States of America	Texas	La Homa Road North Colonia	26.246768	-98.357367	Occurrence Download https://doi.org/10.15468/dl.3bxkun
Dioprosopa clavatus	United States of America	Texas	Hays County	30.255177	-98.040991	Occurrence Download https://doi.org/10.15468/dl.3bxkun
Dioprosopa clavatus	United States of America	Texas	Bastrop County	30.069952	-97.293219	Occurrence Download https://doi.org/10.15468/dl.3bxkun
Dioprosopa clavatus	United States of America	Texas	Hidalgo County	26.38525	-98.263125	Occurrence Download https://doi.org/10.15468/dl.3bxkun
Dioprosopa clavatus	United States of America	Texas	San Antonio	29.666248	-98.648009	Occurrence Download https://doi.org/10.15468/dl.3bxkun
Dioprosopa clavatus	United States of America	Texas	Paloma Lake	30.565154	-97.622361	Occurrence Download https://doi.org/10.15468/dl.3bxkun
Dioprosopa clavatus	United States of America	Texas	Flower Mound	33.024247	-97.12972	Occurrence Download https://doi.org/10.15468/dl.3bxkun
Dioprosopa clavatus	United States of America	Texas	Paloma Lake	30.560054	-97.612035	Occurrence Download https://doi.org/10.15468/dl.3bxkun
Dioprosopa clavatus	United States of America	Texas	Goliad	28.668325	-97.403604	Occurrence Download https://doi.org/10.15468/dl.3bxkun
Dioprosopa clavatus	United States of America	Florida	Polk County	28.007876	-81.82093	Occurrence Download https://doi.org/10.15468/dl.3bxkun
Dioprosopa clavatus	United States of America	Texas	Austin	30.37278	-97.645303	Occurrence Download https://doi.org/10.15468/dl.3bxkun

Dioprosopa clavatus	United States of America	Texas	Maypearl	32.312036	-97.016045	Occurrence Download https://doi.org/10.15468/dl.3bxkun
Dioprosopa clavatus	United States of America	Texas	Mission	26.179528	-98.365957	Occurrence Download https://doi.org/10.15468/dl.3bxkun
Dioprosopa clavatus	United States of America	Texas	Mission	26.179517	-98.366214	Occurrence Download https://doi.org/10.15468/dl.3bxkun
Dioprosopa clavatus	United States of America	Texas	Mission	26.179835	-98.366493	Occurrence Download https://doi.org/10.15468/dl.3bxkun
Dioprosopa clavatus	United States of America	Texas	Austin	30.234983	-97.641567	Occurrence Download https://doi.org/10.15468/dl.3bxkun
Dioprosopa clavatus	United States of America	Texas	Mission	26.180391	-98.330829	Occurrence Download https://doi.org/10.15468/dl.3bxkun
Dioprosopa clavatus	United States of America	California	Redlands	34.033446	-117.207899	Occurrence Download https://doi.org/10.15468/dl.3bxkun
Dioprosopa clavatus	United States of America	Texas	Lubbock	33.524433	-101.960833	Occurrence Download https://doi.org/10.15468/dl.3bxkun
Dioprosopa clavatus	United States of America	Florida	Saint Johns County	29.672067	-81.253625	Occurrence Download https://doi.org/10.15468/dl.3bxkun
Dioprosopa clavatus	United States of America	Florida	Lee Cypress	25.956988	-81.364034	Occurrence Download https://doi.org/10.15468/dl.3bxkun
Dioprosopa clavatus	United States of America	Texas	Corpus Christi	27.645639	-97.299398	Occurrence Download https://doi.org/10.15468/dl.3bxkun
Dioprosopa clavatus	United States of America	Florida	Martin County	26.991647	-80.146064	Occurrence Download https://doi.org/10.15468/dl.3bxkun
Dioprosopa clavatus	Dominican Republic	Puerto Plata	Bisonó	19.6442	-70.857352	Occurrence Download https://doi.org/10.15468/dl.3bxkun
Dioprosopa clavatus	Mexico	Nuevo León	El Cercado	25.398484	-100.134975	Occurrence Download https://doi.org/10.15468/dl.3bxkun
Dioprosopa clavatus	United States of America	California	Ramona	33.04136	-116.879255	Occurrence Download https://doi.org/10.15468/dl.3bxkun
Dioprosopa clavatus	Mexico	Nuevo León	El Cercado	25.398641	-100.13488	Occurrence Download https://doi.org/10.15468/dl.3bxkun
Dioprosopa clavatus	United States of America	Texas	Maypearl	32.313778	-97.014021	Occurrence Download https://doi.org/10.15468/dl.3bxkun
Dioprosopa clavatus	United States of America	Texas	Bastrop County	30.140838	-97.458702	Occurrence Download https://doi.org/10.15468/dl.3bxkun
Dioprosopa clavatus	United States of America	Texas	Lancaster	32.566195	-96.775664	Occurrence Download https://doi.org/10.15468/dl.3bxkun
Dioprosopa clavatus	United States of America	Texas	Lancaster	32.569034	-96.77517	Occurrence Download https://doi.org/10.15468/dl.3bxkun
Dioprosopa clavatus	United States of America	Texas	Georgetown	30.681244	-97.635734	Occurrence Download https://doi.org/10.15468/dl.3bxkun
Dioprosopa clavatus	United States of America	Arizona	Pima County	31.799676	-110.798441	Occurrence Download https://doi.org/10.15468/dl.3bxkun
Dioprosopa clavatus	United States of America	Texas	Maypearl	32.312354	-97.014895	Occurrence Download https://doi.org/10.15468/dl.3bxkun
Dioprosopa clavatus	United States of America	Texas	Maypearl	32.311193	-97.016269	Occurrence Download https://doi.org/10.15468/dl.3bxkun
Dioprosopa clavatus	United States of America	Oklahoma	Norman	35.200233	-97.41486	Occurrence Download https://doi.org/10.15468/dl.3bxkun
Dioprosopa clavatus	United States of America	Texas	Maypearl	32.311087	-97.014895	Occurrence Download https://doi.org/10.15468/dl.3bxkun
Dioprosopa clavatus	Brazil	Rio Grande Sul	Quinta	-32.038377	-52.250633	Occurrence Download https://doi.org/10.15468/dl.3bxkun
Dioprosopa clavatus	United States of America	Texas	Parker County	32.962978	-97.637672	Occurrence Download https://doi.org/10.15468/dl.3bxkun
Dioprosopa clavatus	United States of America	Texas	Maypearl	32.313514	-97.013522	Occurrence Download https://doi.org/10.15468/dl.3bxkun
Dioprosopa clavatus	Mexico	Oaxaca	Santo Domingo Tonalá	17.642262	-97.990959	Occurrence Download https://doi.org/10.15468/dl.3bxkun
Dioprosopa clavatus	Colombia	Antioquia	Ciudad Bolívar	5.792042	-76.036991	Occurrence Download https://doi.org/10.15468/dl.3bxkun
Dioprosopa clavatus	United States of America	Texas	Bastrop County	30.08849	-97.17316	Occurrence Download https://doi.org/10.15468/dl.3bxkun
Dioprosopa clavatus	United States of America	Texas	Antioch	30.09781	-97.16994	Occurrence Download https://doi.org/10.15468/dl.3bxkun
Dioprosopa clavatus	United States of America	Texas	Maypearl	32.312512	-97.012835	Occurrence Download https://doi.org/10.15468/dl.3bxkun
Dioprosopa clavatus	United States of America	Texas	Maypearl	32.310243	-97.011462	Occurrence Download https://doi.org/10.15468/dl.3bxkun
Dioprosopa clavatus	United States of America	Texas	Maypearl	32.308872	-97.01502	Occurrence Download https://doi.org/10.15468/dl.3bxkun
Dioprosopa clavatus	United States of America	Texas	Maypearl	32.311865	-97.015972	Occurrence Download https://doi.org/10.15468/dl.3bxkun
Dioprosopa clavatus	United States of America	Texas	Woodrow	33.448635	-101.980297	Occurrence Download https://doi.org/10.15468/dl.3bxkun
Dioprosopa clavatus	United States of America	Texas	Round Rock	30.560784	-97.620416	Occurrence Download https://doi.org/10.15468/dl.3bxkun

Dioprosopa clavatus	United States of America	Oklahoma	Norman	35.216877	-97.221435	Occurrence Download	https://doi.org/10.15468/dl.3bxkun
Dioprosopa clavatus	United States of America	Texas	Maypearl	32.311113	-97.010513	Occurrence Download	https://doi.org/10.15468/dl.3bxkun
Dioprosopa clavatus	United States of America	Texas	Maypearl	32.311393	-97.012024	Occurrence Download	https://doi.org/10.15468/dl.3bxkun
Dioprosopa clavatus	United States of America	Arizona	Otero	31.572447	-111.046731	Occurrence Download	https://doi.org/10.15468/dl.3bxkun
Dioprosopa clavatus	United States of America	Maryland	Crofton	39.001461	-76.712366	Occurrence Download	https://doi.org/10.15468/dl.3bxkun
Dioprosopa clavatus	United States of America	California	Santa Clara County	37.047567	-121.369086	Occurrence Download	https://doi.org/10.15468/dl.3bxkun
Dioprosopa clavatus	Colombia	Valle del Cauca	Vijes	3.71	-76.43	Occurrence Download	https://doi.org/10.15468/dl.3bxkun
Dioprosopa clavatus	United States of America	California	Galt	38.254637	-121.299948	Occurrence Download	https://doi.org/10.15468/dl.3bxkun
Dioprosopa clavatus	United States of America	Oklahoma	Tryon	35.882425	-96.958238	Occurrence Download	https://doi.org/10.15468/dl.3bxkun
Dioprosopa clavatus	United States of America	Texas	Lubbock County	33.438487	-101.912662	Occurrence Download	https://doi.org/10.15468/dl.3bxkun
Dioprosopa clavatus	United States of America	Texas	Travis County	30.194611	-97.610802	Occurrence Download	https://doi.org/10.15468/dl.3bxkun
Dioprosopa clavatus	United States of America	Texas	Mission	26.177195	-98.365912	Occurrence Download	https://doi.org/10.15468/dl.3bxkun
Dioprosopa clavatus	Mexico	Oaxaca	Santo Domingo Tonalá	17.650225	-97.975369	Occurrence Download	https://doi.org/10.15468/dl.3bxkun
Dioprosopa clavatus	United States of America	Texas	Los Castillos Estates Colonia	26.126408	-97.956389	Occurrence Download	https://doi.org/10.15468/dl.3bxkun
Dioprosopa clavatus	United States of America	Texas	Bastrop County	30.128733	-97.414628	Occurrence Download	https://doi.org/10.15468/dl.3bxkun
Dioprosopa clavatus	Mexico	Nuevo León	Guadalupe	25.639114	-100.207268	Occurrence Download	https://doi.org/10.15468/dl.3bxkun
Dioprosopa clavatus	United States of America	Texas	Mission	26.176784	-98.365681	Occurrence Download	https://doi.org/10.15468/dl.3bxkun
Dioprosopa clavatus	United States of America	Florida	Highlands County	27.298704	-81.422596	Occurrence Download	https://doi.org/10.15468/dl.3bxkun
Dioprosopa clavatus	Argentina	Córdoba	Pedanía Colonias	-32.649036	-62.138829	Occurrence Download	https://doi.org/10.15468/dl.3bxkun
Dioprosopa clavatus	United States of America	Texas	Maypearl	32.311683	-97.015869	Occurrence Download	https://doi.org/10.15468/dl.3bxkun
Dioprosopa clavatus	United States of America	Texas	Nelson	32.311556	-96.854854	Occurrence Download	https://doi.org/10.15468/dl.3bxkun
Dioprosopa clavatus	United States of America	Virginia	Rutherford Village	37.469337	-77.669603	Occurrence Download	https://doi.org/10.15468/dl.3bxkun
Dioprosopa clavatus	United States of America	Texas	Maypearl	32.311697	-97.015885	Occurrence Download	https://doi.org/10.15468/dl.3bxkun
Dioprosopa clavatus	Mexico	Morelos	Tepoztlán	18.991743	-99.116461	Occurrence Download	https://doi.org/10.15468/dl.3bxkun
Dioprosopa clavatus	United States of America	Texas	Bastrop County	30.128731	-97.414697	Occurrence Download	https://doi.org/10.15468/dl.3bxkun
Dioprosopa clavatus	United States of America	Texas	Johnson County	32.54017	-97.465321	Occurrence Download	https://doi.org/10.15468/dl.3bxkun
Dioprosopa clavatus	United States of America	Texas	Waxahachie	32.311318	-96.854353	Occurrence Download	https://doi.org/10.15468/dl.3bxkun
Dioprosopa clavatus	United States of America	Texas	Midlothian	32.497978	-96.959287	Occurrence Download	https://doi.org/10.15468/dl.3bxkun
Dioprosopa clavatus	United States of America	Texas	San Marcos	29.873096	-97.962529	Occurrence Download	https://doi.org/10.15468/dl.3bxkun
Dioprosopa clavatus	United States of America	Texas	Nelson	32.311411	-96.854683	Occurrence Download	https://doi.org/10.15468/dl.3bxkun
Dioprosopa clavatus	United States of America	Oklahoma	Kiowa County	34.745751	-98.969068	Occurrence Download	https://doi.org/10.15468/dl.3bxkun
Dioprosopa clavatus	United States of America	Texas	Georgetown	30.68182	-97.635337	Occurrence Download	https://doi.org/10.15468/dl.3bxkun
Dioprosopa clavatus	United States of America	California	Los Angeles County	34.392644	-118.655586	Occurrence Download	https://doi.org/10.15468/dl.3bxkun
Dioprosopa clavatus	United States of America	Virginia	Avondale	37.660314	-77.346863	Occurrence Download	https://doi.org/10.15468/dl.3bxkun
Dioprosopa clavatus	United States of America	Texas	Maypearl	32.311748	-97.015945	Occurrence Download	https://doi.org/10.15468/dl.3bxkun
Dioprosopa clavatus	United States of America	Virginia	Harbor View	38.672071	-77.229402	Occurrence Download	https://doi.org/10.15468/dl.3bxkun
Dioprosopa clavatus	United States of America	Texas	Georgetown	30.681137	-97.635552	Occurrence Download	https://doi.org/10.15468/dl.3bxkun
Dioprosopa clavatus	United States of America	California	Chula Vista	32.643319	-116.947765	Occurrence Download	https://doi.org/10.15468/dl.3bxkun
Dioprosopa clavatus	Mexico	Nuevo León	Los Canelos	25.429675	-100.127425	Occurrence Download	https://doi.org/10.15468/dl.3bxkun

Dioprosopa clavatus	United States of America	Texas	Weslaco	26.126124	-97.957825	Occurrence Download https://doi.org/10.15468/dl.3bxkun
Dioprosopa clavatus	United States of America	Texas	Maypearl	32.311697	-97.015955	Occurrence Download https://doi.org/10.15468/dl.3bxkun
Dioprosopa clavatus	United States of America	Texas	Texas City	29.4076	-94.89192	Occurrence Download https://doi.org/10.15468/dl.3bxkun
Dioprosopa clavatus	United States of America	Texas	Mission	26.178583	-98.366514	Occurrence Download https://doi.org/10.15468/dl.3bxkun
Dioprosopa clavatus	Peru	Lima	Santa Eulalia	-11.82	-76.6294	Occurrence Download https://doi.org/10.15468/dl.3bxkun
Dioprosopa clavatus	United States of America	Florida	Saint Johns County	29.750022	-81.289192	Occurrence Download https://doi.org/10.15468/dl.3bxkun
Dioprosopa clavatus	Mexico	Yucatán	Ek Balam	20.888	-88.128	Occurrence Download https://doi.org/10.15468/dl.3bxkun
Dioprosopa clavatus	Mexico	Yucatán	Ek Balam	20.888047	-88.128361	Occurrence Download https://doi.org/10.15468/dl.3bxkun
Dioprosopa clavatus	Argentina	Santa Fe		-31.54659	-61.059982	Occurrence Download https://doi.org/10.15468/dl.3bxkun
Dioprosopa clavatus	United States of America	Texas	Weslaco	26.12612	-97.957792	Occurrence Download https://doi.org/10.15468/dl.3bxkun
Dioprosopa clavatus	United States of America	Texas	Smithville	30.004231	-97.151925	Occurrence Download https://doi.org/10.15468/dl.3bxkun
Dioprosopa clavatus	United States of America	Texas	Frisco	33.110722	-96.853287	Occurrence Download https://doi.org/10.15468/dl.3bxkun
Dioprosopa clavatus	United States of America	Illinois	Iroquois County	40.528472	-88.071754	Occurrence Download https://doi.org/10.15468/dl.3bxkun
Dioprosopa clavatus	United States of America	Texas	Hidalgo County	26.081715	-98.135975	Occurrence Download https://doi.org/10.15468/dl.3bxkun
Dioprosopa clavatus	Brazil	Mato Grosso	Chapada dos Guimarães	-15.458295	-55.752458	Occurrence Download https://doi.org/10.15468/dl.3bxkun
Dioprosopa clavatus	United States of America	Texas	Mansfield	32.595255	-97.16046	Occurrence Download https://doi.org/10.15468/dl.3bxkun
Dioprosopa clavatus	Brazil	Paraná	Palmas	-26.5025	-51.6756	Occurrence Download https://doi.org/10.15468/dl.3bxkun
Dioprosopa clavatus	Brazil	Paraná	Palmas	-26.5008	-51.6764	Occurrence Download https://doi.org/10.15468/dl.3bxkun
Dioprosopa clavatus	United States of America	California	Saxon	38.494305	-121.662252	Occurrence Download https://doi.org/10.15468/dl.3bxkun
Dioprosopa clavatus	Brazil	Paraná	Palmas	-26.5025	-51.6739	Occurrence Download https://doi.org/10.15468/dl.3bxkun
Dioprosopa clavatus	United States of America	Missouri	Columbia	38.907803	-92.296962	Occurrence Download https://doi.org/10.15468/dl.3bxkun
Dioprosopa clavatus	United States of America	Oklahoma	Seminole	35.22452	-96.670573	Occurrence Download https://doi.org/10.15468/dl.3bxkun
Dioprosopa clavatus	United States of America	Arizona	Cochise County	31.883547	-109.206172	Occurrence Download https://doi.org/10.15468/dl.3bxkun
Dioprosopa clavatus	Brazil	Paraná	Palmas	-26.5019	-51.6764	Occurrence Download https://doi.org/10.15468/dl.3bxkun
Dioprosopa clavatus	Brazil	Paraná		-26.5586	-51.5394	Occurrence Download https://doi.org/10.15468/dl.3bxkun
Dioprosopa clavatus	Brazil	Paraná		-26.5572	-51.5422	Occurrence Download https://doi.org/10.15468/dl.3bxkun
Dioprosopa clavatus	United States of America	Florida	Archbold	27.182843	-81.352007	Occurrence Download https://doi.org/10.15468/dl.3bxkun
Dioprosopa clavatus	Costa Rica	San José	San José	10.049505	-84.015882	Occurrence Download https://doi.org/10.15468/dl.3bxkun
Dioprosopa clavatus	United States of America	Florida	Palm Beach County	26.499273	-80.212371	Occurrence Download https://doi.org/10.15468/dl.3bxkun
Dioprosopa clavatus	Mexico	Coahuila	Antiguos Mineros del Norte	26.782778	-102.005555	Occurrence Download https://doi.org/10.15468/dl.3bxkun
Dioprosopa clavatus	Mexico	Coahuila	Ocampo	27.088056	-102.401111	Occurrence Download https://doi.org/10.15468/dl.3bxkun
Dioprosopa clavatus	United States of America	California	Laguna Beach	33.542719	-117.785357	Occurrence Download https://doi.org/10.15468/dl.3bxkun
Dioprosopa clavatus	Mexico	Coahuila	Cuatro Ciénegas de Carranza	27.012222	-102.083611	Occurrence Download https://doi.org/10.15468/dl.3bxkun
Dioprosopa clavatus	Mexico	Coahuila	Ocampo	27.210278	-102.464167	Occurrence Download https://doi.org/10.15468/dl.3bxkun
Dioprosopa clavatus	United States of America	Florida	DeLand	29.038533	-81.334786	Occurrence Download https://doi.org/10.15468/dl.3bxkun
Dioprosopa clavatus	Mexico	Coahuila	Ocampo	27.221944	-102.505278	Occurrence Download https://doi.org/10.15468/dl.3bxkun
Dioprosopa clavatus	Mexico	Coahuila	Cuatro Ciénegas	27.052222	-102.226389	Occurrence Download https://doi.org/10.15468/dl.3bxkun
Dioprosopa clavatus	United States of America	Kentucky	Warren County	36.954165	-86.529416	Occurrence Download https://doi.org/10.15468/dl.3bxkun
Dioprosopa clavatus	Mexico	Coahuila	Ocampo	27.110833	-102.394722	Occurrence Download https://doi.org/10.15468/dl.3bxkun

Dioprosopa clavatus	Brazil	Paraná	Antonina	-25.3133	-48.6697	Occurrence Download https://doi.org/10.15468/dl.3bxkun
Dioprosopa clavatus	United States of America	South Carolina	Vermillion	34.392603	-82.350333	Occurrence Download https://doi.org/10.15468/dl.3bxkun
Dioprosopa clavatus	Brazil	Paraná	Antonina	-25.3647	-48.7753	Occurrence Download https://doi.org/10.15468/dl.3bxkun
Dioprosopa clavatus	Brazil	Paraná	Antonina	-25.3125	-48.6967	Occurrence Download https://doi.org/10.15468/dl.3bxkun
Dioprosopa clavatus	Brazil	Paraná		-25.2886	-48.6836	Occurrence Download https://doi.org/10.15468/dl.3bxkun
Dioprosopa clavatus	Puerto Rico	Puerto Rico	Llanos	18.013852	-67.101012	Occurrence Download https://doi.org/10.15468/dl.3bxkun
Dioprosopa clavatus	Brazil	Paraná	Antonina	-25.3161	-48.6614	Occurrence Download https://doi.org/10.15468/dl.3bxkun
Dioprosopa clavatus	Colombia	Antioquia	Cabecera Municipal	5.592065	-75.813504	Occurrence Download https://doi.org/10.15468/dl.3bxkun
Dioprosopa clavatus	Puerto Rico	Puerto Rico	Cruce de Jauca	18.1725	-66.591575	Occurrence Download https://doi.org/10.15468/dl.3bxkun
Dioprosopa clavatus	United States of America	California	Unincorporated Santa Monica Mountains	34.1	-118.79	Occurrence Download https://doi.org/10.15468/dl.3bxkun
Dioprosopa clavatus	United States of America	California	Thousand Oaks	34.13	-118.86	Occurrence Download https://doi.org/10.15468/dl.3bxkun
Dioprosopa clavatus	Brazil	Paraná	Antonina	-25.3128	-48.6692	Occurrence Download https://doi.org/10.15468/dl.3bxkun
Dioprosopa clavatus	Colombia	Antioquia	Cruces	6.54811	-75.08268	Occurrence Download https://doi.org/10.15468/dl.3bxkun
Dioprosopa clavatus	Colombia	Antioquia	La Ica	6.487796	-74.834162	Occurrence Download https://doi.org/10.15468/dl.3bxkun
Dioprosopa clavatus	United States of America	Texas	Wayside	30.3	-97.294	Occurrence Download https://doi.org/10.15468/dl.3bxkun
Dioprosopa clavatus	United States of America	Texas	Oak Hill	30.236	-97.275	Occurrence Download https://doi.org/10.15468/dl.3bxkun
Dioprosopa clavatus	United States of America	Texas	Bastrop County	30.286	-97.305	Occurrence Download https://doi.org/10.15468/dl.3bxkun
Dioprosopa clavatus	United States of America	Texas	Bastrop County	30.278	-97.277	Occurrence Download https://doi.org/10.15468/dl.3bxkun
Dioprosopa clavatus	United States of America	California	Unincorporated Santa Monica Mountains	34.1	-118.7	Occurrence Download https://doi.org/10.15468/dl.3bxkun
Dioprosopa clavatus	United States of America	Texas	Brown County	31.656	-98.949	Occurrence Download https://doi.org/10.15468/dl.3bxkun
Dioprosopa clavatus	United States of America	Texas	Pflugerville	30.437	-97.647	Occurrence Download https://doi.org/10.15468/dl.3bxkun
Dioprosopa clavatus	United States of America	Texas	Lamar County	33.79048	-95.58397	Occurrence Download https://doi.org/10.15468/dl.3bxkun
Dioprosopa clavatus	United States of America	Texas	Uvalde	29.1845	-99.792251	Occurrence Download https://doi.org/10.15468/dl.3bxkun
Dioprosopa clavatus	United States of America	Georgia	Oglethorpe County	33.878	-83.228	Occurrence Download https://doi.org/10.15468/dl.3bxkun
Dioprosopa clavatus	Colombia	Quindío	Filandia	4.686111	-75.617222	Occurrence Download https://doi.org/10.15468/dl.3bxkun
Dioprosopa clavatus	United States of America	Texas	Brown County	31.597	-98.902	Occurrence Download https://doi.org/10.15468/dl.3bxkun
Dioprosopa clavatus	United States of America	Texas	Brown County	31.628	-98.906	Occurrence Download https://doi.org/10.15468/dl.3bxkun
Dioprosopa clavatus	United States of America	Florida	Archbold	27.18333	-81.35139	Occurrence Download https://doi.org/10.15468/dl.3bxkun
Dioprosopa clavatus	United States of America	Texas	Denton	33.147376	-97.126152	Occurrence Download https://doi.org/10.15468/dl.3bxkun
Dioprosopa clavatus	United States of America	Florida	Childs	27.20167	-81.3475	Occurrence Download https://doi.org/10.15468/dl.3bxkun
Dioprosopa clavatus	United States of America	New Mexico	Chaves County	33.451967	-104.397305	Occurrence Download https://doi.org/10.15468/dl.3bxkun
Dioprosopa clavatus	Brazil	Roraima	Uiramutã	4.63055	-60.162	Occurrence Download https://doi.org/10.15468/dl.3bxkun
Dioprosopa clavatus	Mexico	Colima	Las Trancas	19.05417	-103.72472	Occurrence Download https://doi.org/10.15468/dl.3bxkun
Dioprosopa clavatus	Mexico	Colima	Las Trancas	19.0542	-103.7247	Occurrence Download https://doi.org/10.15468/dl.3bxkun
Dioprosopa clavatus	United States of America	Texas	Austin	30.3697	-97.8331	Occurrence Download https://doi.org/10.15468/dl.3bxkun
Dioprosopa clavatus	United States of America	Texas	San Saba County	31.0375	-98.4478	Occurrence Download https://doi.org/10.15468/dl.3bxkun
Dioprosopa clavatus	Colombia	Antioquia	La Pintada	5.723333	-75.620833	Occurrence Download https://doi.org/10.15468/dl.3bxkun
Dioprosopa clavatus	Colombia	Antioquia	San Lorenzo	6.372056	-75.07275	Occurrence Download https://doi.org/10.15468/dl.3bxkun
Dioprosopa clavatus	Colombia	Antioquia	El Carbón	6.34052	-75.12484	Occurrence Download https://doi.org/10.15468/dl.3bxkun

Dioprosopa clavatus	United States of America	Arizona	Cochise County	31.75889	-109.68917	Occurrence Download https://doi.org/10.15468/dl.3bxkun
Dioprosopa clavatus	United States of America	New Mexico	Grant County	32.9	-108.55	Occurrence Download https://doi.org/10.15468/dl.3bxkun
Dioprosopa clavatus	United States of America	New Mexico	Sherman	32.71667	-107.85	Occurrence Download https://doi.org/10.15468/dl.3bxkun
Dioprosopa clavatus	United States of America	Arizona	Cochise County	31.46472	-109.69944	Occurrence Download https://doi.org/10.15468/dl.3bxkun
Dioprosopa clavatus	Costa Rica	San José	Colón	9.911663	-84.281597	Occurrence Download https://doi.org/10.15468/dl.3bxkun
Dioprosopa clavatus	Costa Rica	Guanacaste	Carmona	10.023056	-85.239444	Occurrence Download https://doi.org/10.15468/dl.3bxkun
Dioprosopa clavatus	Costa Rica	Guanacaste	Santa Rita	10.044298	-85.240526	Occurrence Download https://doi.org/10.15468/dl.3bxkun
Dioprosopa clavatus	Costa Rica	Puntarenas	Sabalito	8.911111	-82.786111	Occurrence Download https://doi.org/10.15468/dl.3bxkun
Dioprosopa clavatus	Costa Rica	Guanacaste	San Pablo	10.054558	-85.235843	Occurrence Download https://doi.org/10.15468/dl.3bxkun
Dioprosopa clavatus	Mexico	Colima	Las Trancas	19.05944	-103.72278	Occurrence Download https://doi.org/10.15468/dl.3bxkun
Dioprosopa clavatus	Mexico	Colima	Las Trancas	19.0594	-103.7228	Occurrence Download https://doi.org/10.15468/dl.3bxkun
Dioprosopa clavatus	Mexico	Colima	Ixtlahuacán	19.02556	-103.71361	Occurrence Download https://doi.org/10.15468/dl.3bxkun
Dioprosopa clavatus	Mexico	Colima	Zinacamitlán (Los Chicos)	18.98611	-103.70361	Occurrence Download https://doi.org/10.15468/dl.3bxkun
Dioprosopa clavatus	Mexico	Colima	La Presa (Barranca del Rebozo)	19.01111	-103.70667	Occurrence Download https://doi.org/10.15468/dl.3bxkun
Dioprosopa clavatus	United States of America	Missouri	Columbia	38.949148	-92.393261	Occurrence Download https://doi.org/10.15468/dl.3bxkun
Dioprosopa clavatus	Mexico	Colima	Ixtlahuacán	19.0256	-103.7136	Occurrence Download https://doi.org/10.15468/dl.3bxkun
Dioprosopa clavatus	Mexico	Colima	La Presa (Barranca del Rebozo)	19.0111	-103.7067	Occurrence Download https://doi.org/10.15468/dl.3bxkun
Dioprosopa clavatus	Mexico	Colima	Zinacamitlán (Los Chicos)	18.9861	-103.7036	Occurrence Download https://doi.org/10.15468/dl.3bxkun
Dioprosopa clavatus	United States of America	Texas	Mission	26.179293	-98.366524	Occurrence Download https://doi.org/10.15468/dl.3bxkun
Dioprosopa clavatus	United States of America	Tamaulipas	Mission	26.185945	-98.379832	Occurrence Download https://doi.org/10.15468/dl.3bxkun
Dioprosopa clavatus	Mexico	Oaxaca	Santa María Huatulco	15.8494	-96.1114	Occurrence Download https://doi.org/10.15468/dl.3bxkun
Dioprosopa clavatus	Mexico	Oaxaca	Santa María Huatulco	15.84944	-96.11139	Occurrence Download https://doi.org/10.15468/dl.3bxkun
Dioprosopa clavatus	Costa Rica	Alajuela	Bijagua	10.724378	-84.997041	Occurrence Download https://doi.org/10.15468/dl.3bxkun
Dioprosopa clavatus	Costa Rica	Alajuela	Peñas Blancas	10.32112	-84.725931	Occurrence Download https://doi.org/10.15468/dl.3bxkun
Dioprosopa clavatus	Costa Rica	Guanacaste	Quebrada Nando	9.761546	-85.2263	Occurrence Download https://doi.org/10.15468/dl.3bxkun
Dioprosopa clavatus	Mexico	Oaxaca	Puente de Coyula	15.7494	-96.2978	Occurrence Download https://doi.org/10.15468/dl.3bxkun
Dioprosopa clavatus	Mexico	Oaxaca	Puente de Coyula	15.74944	-96.29778	Occurrence Download https://doi.org/10.15468/dl.3bxkun
Dioprosopa clavatus	United States of America	Texas	Kerr County	29.992	-99.388	Occurrence Download https://doi.org/10.15468/dl.3bxkun
Dioprosopa clavatus	United States of America	Texas	Travis County	30.334	-97.888	Occurrence Download https://doi.org/10.15468/dl.3bxkun
Dioprosopa clavatus	Mexico	Oaxaca	La Crucecita	15.7694	-96.1944	Occurrence Download https://doi.org/10.15468/dl.3bxkun
Dioprosopa clavatus	Mexico	Oaxaca	La Crucecita	15.76944	-96.19444	Occurrence Download https://doi.org/10.15468/dl.3bxkun
Dioprosopa clavatus	Mexico	Oaxaca	La Crucecita	15.8161	-96.1758	Occurrence Download https://doi.org/10.15468/dl.3bxkun
Dioprosopa clavatus	Mexico	Oaxaca	La Crucecita	15.81611	-96.17583	Occurrence Download https://doi.org/10.15468/dl.3bxkun
Dioprosopa clavatus	Mexico	Oaxaca	La Crucecita	15.8058	-96.1678	Occurrence Download https://doi.org/10.15468/dl.3bxkun
Dioprosopa clavatus	Mexico	Oaxaca	Santa María Huatulco	15.7725	-96.2344	Occurrence Download https://doi.org/10.15468/dl.3bxkun
Dioprosopa clavatus	Mexico	Oaxaca	La Crucecita	15.80583	-96.16778	Occurrence Download https://doi.org/10.15468/dl.3bxkun
Dioprosopa clavatus	Mexico	Oaxaca	Santa María Huatulco	15.7725	-96.23444	Occurrence Download https://doi.org/10.15468/dl.3bxkun
Dioprosopa clavatus	United States of America	Florida	Wakulla County	30.125501	-84.285539	Occurrence Download https://doi.org/10.15468/dl.3bxkun
Dioprosopa clavatus	Costa Rica	Puntarenas	Golfito	8.649823	-83.16058	Occurrence Download https://doi.org/10.15468/dl.3bxkun

Dioprosopa clavatus	Costa Rica	Puntarenas	Porvenir	9.94944	-85.272625	Occurrence Download https://doi.org/10.15468/dl.3bxkun
Dioprosopa clavatus	Mexico	Sonora	San Javier	28.6042	-109.7717	Occurrence Download https://doi.org/10.15468/dl.3bxkun
Dioprosopa clavatus	Mexico	Sonora	San Javier	28.5444	-109.6906	Occurrence Download https://doi.org/10.15468/dl.3bxkun
Dioprosopa clavatus	Mexico	Sonora	Lo de Campa	28.5381	-109.7433	Occurrence Download https://doi.org/10.15468/dl.3bxkun
Dioprosopa clavatus	Mexico	Sonora	El Entronque	28.5522	-109.7258	Occurrence Download https://doi.org/10.15468/dl.3bxkun
Dioprosopa clavatus	Mexico	Sonora	San Javier	28.5733	-109.665	Occurrence Download https://doi.org/10.15468/dl.3bxkun
Dioprosopa clavatus	Mexico	Sonora	San Javier	28.5392	-109.6917	Occurrence Download https://doi.org/10.15468/dl.3bxkun
Dioprosopa clavatus	Mexico	Sonora	San Javier	28.544444	-109.690556	Occurrence Download https://doi.org/10.15468/dl.3bxkun
Dioprosopa clavatus	Mexico	Sonora	El Entronque	28.552222	-109.725833	Occurrence Download https://doi.org/10.15468/dl.3bxkun
Dioprosopa clavatus	Mexico	Sonora	San Javier	28.539167	-109.691667	Occurrence Download https://doi.org/10.15468/dl.3bxkun
Dioprosopa clavatus	Mexico	Sonora	Lo de Campa	28.538056	-109.743333	Occurrence Download https://doi.org/10.15468/dl.3bxkun
Dioprosopa clavatus	Mexico	Sonora	San Javier	28.573333	-109.665	Occurrence Download https://doi.org/10.15468/dl.3bxkun
Dioprosopa clavatus	Mexico	Sonora	San Javier	28.604167	-109.771667	Occurrence Download https://doi.org/10.15468/dl.3bxkun
Dioprosopa clavatus	United States of America	Oklahoma	Hughes County	34.885692	-96.250634	Occurrence Download https://doi.org/10.15468/dl.3bxkun
Dioprosopa clavatus	Mexico	Sonora	San Javier	28.6114	-109.7564	Occurrence Download https://doi.org/10.15468/dl.3bxkun
Dioprosopa clavatus	Mexico	Sonora	San Javier	28.611389	-109.756389	Occurrence Download https://doi.org/10.15468/dl.3bxkun
Dioprosopa clavatus	Mexico	Sonora	San Javier	28.5939	-109.7394	Occurrence Download https://doi.org/10.15468/dl.3bxkun
Dioprosopa clavatus	Mexico	Sonora	San Javier	28.5517	-109.7056	Occurrence Download https://doi.org/10.15468/dl.3bxkun
Dioprosopa clavatus	Mexico	Sonora	San Javier	28.593889	-109.739444	Occurrence Download https://doi.org/10.15468/dl.3bxkun
Dioprosopa clavatus	Mexico	Sonora	San Javier	28.551667	-109.705556	Occurrence Download https://doi.org/10.15468/dl.3bxkun
Dioprosopa clavatus	United States of America	Texas	Bastrop County	30.264	-97.296	Occurrence Download https://doi.org/10.15468/dl.3bxkun
Dioprosopa clavatus	United States of America	Texas	Bastrop County	30.284	-97.32	Occurrence Download https://doi.org/10.15468/dl.3bxkun
Dioprosopa clavatus	United States of America	New Mexico	Chaves County	33.602	-104.412	Occurrence Download https://doi.org/10.15468/dl.3bxkun
Dioprosopa clavatus	United States of America	California	Los Angeles	33.736062	-118.292246	Occurrence Download https://doi.org/10.15468/dl.3bxkun
Dioprosopa clavatus	Costa Rica	Guanacaste	Santa Cruz	10.134259	-85.633597	Occurrence Download https://doi.org/10.15468/dl.3bxkun
Dioprosopa clavatus	Costa Rica	Guanacaste	Tierras Morenas	10.580475	-84.987588	Occurrence Download https://doi.org/10.15468/dl.3bxkun
Dioprosopa clavatus	Costa Rica	Guanacaste	Santa Elena	10.83641	-85.615491	Occurrence Download https://doi.org/10.15468/dl.3bxkun
Dioprosopa clavatus	Costa Rica	Guanacaste	Tierras Morenas	10.579924	-84.987587	Occurrence Download https://doi.org/10.15468/dl.3bxkun
Dioprosopa clavatus	Costa Rica	Cartago	Chirripó	9.968015	-83.468769	Occurrence Download https://doi.org/10.15468/dl.3bxkun
Dioprosopa clavatus	Costa Rica	Guanacaste	Liberia	10.772778	-85.311944	Occurrence Download https://doi.org/10.15468/dl.3bxkun
Dioprosopa clavatus	Costa Rica	Alajuela	Bijagua	10.712528	-85.04089	Occurrence Download https://doi.org/10.15468/dl.3bxkun
Dioprosopa clavatus	Costa Rica	Guanacaste	San Antonio	10.170061	-85.362712	Occurrence Download https://doi.org/10.15468/dl.3bxkun
Dioprosopa clavatus	United States of America	Texas	Bastrop County	30.08463	-97.1682	Occurrence Download https://doi.org/10.15468/dl.3bxkun
Dioprosopa clavatus	Costa Rica	Alajuela	Bijagua	10.718881	-85.029934	Occurrence Download https://doi.org/10.15468/dl.3bxkun
Dioprosopa clavatus	Costa Rica	Alajuela	Dos Ríos	10.876985	-85.399802	Occurrence Download https://doi.org/10.15468/dl.3bxkun
Dioprosopa clavatus	United States of America	Texas	Bastrop County	30.08877	-97.17023	Occurrence Download https://doi.org/10.15468/dl.3bxkun
Dioprosopa clavatus	Costa Rica	Puntarenas	Sabalito	8.89549	-82.771106	Occurrence Download https://doi.org/10.15468/dl.3bxkun
Dioprosopa clavatus	Costa Rica	Puntarenas	Sabalito	8.903123	-82.750167	Occurrence Download https://doi.org/10.15468/dl.3bxkun
Dioprosopa clavatus	Costa Rica	Puntarenas	Sabalito	8.920853	-82.789501	Occurrence Download https://doi.org/10.15468/dl.3bxkun

Dioprosopa clavatus	Costa Rica	Alajuela	La Fortuna	10.463489	-84.755336	Occurrence Download https://doi.org/10.15468/dl.3bxkun
Dioprosopa clavatus	Costa Rica	Guanacaste	Santa Cruz	10.162491	-85.583068	Occurrence Download https://doi.org/10.15468/dl.3bxkun
Dioprosopa clavatus	Costa Rica	Puntarenas	Sabalito	8.908164	-82.777431	Occurrence Download https://doi.org/10.15468/dl.3bxkun
Dioprosopa clavatus	Brazil	Paraná		-25.3	-50.3	Occurrence Download https://doi.org/10.15468/dl.3bxkun
Dioprosopa clavatus	Costa Rica	Puntarenas	Gutiérrez Braun	8.951251	-82.835532	Occurrence Download https://doi.org/10.15468/dl.3bxkun
Dioprosopa clavatus	Costa Rica	Puntarenas	Guacimal	10.276723	-84.79662	Occurrence Download https://doi.org/10.15468/dl.3bxkun
Dioprosopa clavatus	Brazil	Rio Grande do Sul	São Francisco de Paula	-29.480833	-50.174444	Occurrence Download https://doi.org/10.15468/dl.3bxkun
Dioprosopa clavatus	Costa Rica	Puntarenas	Gutiérrez Braun	8.955806	-82.850526	Occurrence Download https://doi.org/10.15468/dl.3bxkun
Dioprosopa clavatus	Costa Rica	Puntarenas	Sabalito	8.924486	-82.796494	Occurrence Download https://doi.org/10.15468/dl.3bxkun
Dioprosopa clavatus	Costa Rica	Puntarenas	Gutiérrez Braun	8.985606	-82.832725	Occurrence Download https://doi.org/10.15468/dl.3bxkun
Dioprosopa clavatus	Brazil	Rio Grande do Sul	Porto Alegre	-30.019139	-51.237222	Occurrence Download https://doi.org/10.15468/dl.3bxkun
Dioprosopa clavatus	Venezuela	Lara	Sanare	9.7	-69.585	Occurrence Download https://doi.org/10.15468/dl.3bxkun
Dioprosopa clavatus	Venezuela	Aragua	Cansamacho La Trilla	10.35	-67.6833	Occurrence Download https://doi.org/10.15468/dl.3bxkun
Dioprosopa clavatus	Costa Rica	San José	Rivas	9.463958	-83.557392	Occurrence Download https://doi.org/10.15468/dl.3bxkun
Dioprosopa clavatus	Costa Rica	Guanacaste	Mayorga	10.92909	-85.495119	Occurrence Download https://doi.org/10.15468/dl.3bxkun
Dioprosopa clavatus	Costa Rica	Guanacaste	Mayorga	10.929156	-85.476824	Occurrence Download https://doi.org/10.15468/dl.3bxkun
Dioprosopa clavatus	Costa Rica	Guanacaste	Santa Elena	10.955326	-85.491568	Occurrence Download https://doi.org/10.15468/dl.3bxkun
Dioprosopa clavatus	Costa Rica	Puntarenas	Sabalito	8.913153	-82.783792	Occurrence Download https://doi.org/10.15468/dl.3bxkun
Dioprosopa clavatus	Costa Rica	Puntarenas	Sabalito	8.916343	-82.79424	Occurrence Download https://doi.org/10.15468/dl.3bxkun
Dioprosopa clavatus	Costa Rica	Alajuela	Dos Ríos	10.882909	-85.401652	Occurrence Download https://doi.org/10.15468/dl.3bxkun
Dioprosopa clavatus	Costa Rica	Puntarenas	Sabalito	8.915892	-82.794696	Occurrence Download https://doi.org/10.15468/dl.3bxkun
Dioprosopa clavatus	United States of America	Georgia	Jefferson	34.133883	-83.566348	Occurrence Download https://doi.org/10.15468/dl.3bxkun
Dioprosopa clavatus	Costa Rica	Puntarenas	Golfito	8.69152	-83.238901	Occurrence Download https://doi.org/10.15468/dl.3bxkun
Dioprosopa clavatus	Costa Rica	Puntarenas	Tárcoles	9.774233	-84.608124	Occurrence Download https://doi.org/10.15468/dl.3bxkun
Dioprosopa clavatus	Costa Rica	San José	Rivas	9.473911	-83.585619	Occurrence Download https://doi.org/10.15468/dl.3bxkun
Dioprosopa clavatus	Costa Rica	Puntarenas	San Vito	8.830339	-82.957619	Occurrence Download https://doi.org/10.15468/dl.3bxkun
Dioprosopa clavatus	United States of America	Georgia	Long County	31.67612	-81.779793	Occurrence Download https://doi.org/10.15468/dl.3bxkun
Dioprosopa clavatus	Costa Rica	Puntarenas	Pittier	8.970465	-82.941886	Occurrence Download https://doi.org/10.15468/dl.3bxkun
Dioprosopa clavatus	Puerto Rico	Puerto Rico	Guerrero	18.46285	-67.051482	Occurrence Download https://doi.org/10.15468/dl.3bxkun
Dioprosopa clavatus	Puerto Rico	Puerto Rico	Isabela	18.501022	-67.023583	Occurrence Download https://doi.org/10.15468/dl.3bxkun
Dioprosopa clavatus	Puerto Rico	Puerto Rico	Villalba	18.128772	-66.492508	Occurrence Download https://doi.org/10.15468/dl.3bxkun
Dioprosopa clavatus	Costa Rica	Guanacaste	Tierras Morenas	10.571062	-85.025949	Occurrence Download https://doi.org/10.15468/dl.3bxkun
Dioprosopa clavatus	Mexico	Baixa Califórnia Sur	Municipio de La Paz	24.25	-110.3333	Occurrence Download https://doi.org/10.15468/dl.3bxkun
Dioprosopa clavatus	Mexico	Baixa Califórnia Sur	La Paz	24.1569	-110.3333	Occurrence Download https://doi.org/10.15468/dl.3bxkun
Dioprosopa clavatus	Costa Rica	Guanacaste	Santa Cecilia	10.992609	-85.429477	Occurrence Download https://doi.org/10.15468/dl.3bxkun
Dioprosopa clavatus	United States of America	Texas	Blanco County	30.34	-98.25	Occurrence Download https://doi.org/10.15468/dl.3bxkun
Dioprosopa clavatus	Nicaragua	Masaya	Masaya	12.004444	-86.01972	Occurrence Download https://doi.org/10.15468/dl.3bxkun
Dioprosopa clavatus	United States of America	California	Topanga	34.08	-118.6	Occurrence Download https://doi.org/10.15468/dl.3bxkun
Dioprosopa clavatus	Nicaragua	Matagalpa	Fuente Pura	13.010834	-85.92083	Occurrence Download https://doi.org/10.15468/dl.3bxkun

Dioprosopa clavatus	Costa Rica	Guanacaste	La Garita	11.034298	-85.52754	Occurrence Download https://doi.org/10.15468/dl.3bxkun
Dioprosopa clavatus	Costa Rica	Guanacaste	Santa Elena	10.901925	-85.731015	Occurrence Download https://doi.org/10.15468/dl.3bxkun
Dioprosopa clavatus	Costa Rica	Guanacaste	San Antonio	10.168217	-85.373656	Occurrence Download https://doi.org/10.15468/dl.3bxkun
Dioprosopa clavatus	Costa Rica	Guanacaste	Santa Cecilia	11.012505	-85.427717	Occurrence Download https://doi.org/10.15468/dl.3bxkun
Dioprosopa clavatus	Costa Rica	Heredia	La Virgen	10.466407	-84.116393	Occurrence Download https://doi.org/10.15468/dl.3bxkun
Dioprosopa clavatus	United States of America	Alabama	Lawrence County	34.521651	-87.311045	Occurrence Download https://doi.org/10.15468/dl.3bxkun
Dioprosopa clavatus	Nicaragua	León	León	12.431667	-86.87778	Occurrence Download https://doi.org/10.15468/dl.3bxkun
Dioprosopa clavatus	Costa Rica	Guanacaste	La Garita	11.033856	-85.524793	Occurrence Download https://doi.org/10.15468/dl.3bxkun
Dioprosopa clavatus	Costa Rica	Guanacaste	Nacascolo	10.705454	-85.579301	Occurrence Download https://doi.org/10.15468/dl.3bxkun
Dioprosopa clavatus	Costa Rica	Guanacaste	Mayorga	10.897554	-85.465274	Occurrence Download https://doi.org/10.15468/dl.3bxkun
Dioprosopa clavatus	Costa Rica	Puntarenas	Bahía Drake	8.679096	-83.566714	Occurrence Download https://doi.org/10.15468/dl.3bxkun
Dioprosopa clavatus	Costa Rica	Puntarenas	Sabalito	8.891473	-82.792936	Occurrence Download https://doi.org/10.15468/dl.3bxkun
Dioprosopa clavatus	Mexico	Tamaulipas	Hidalgo County	26.17	-98.38	Occurrence Download https://doi.org/10.15468/dl.3bxkun
Dioprosopa clavatus	United States of America	Texas	Hillcrest Terrace Colonia	26.381517	-98.114651	Occurrence Download https://doi.org/10.15468/dl.3bxkun
Dioprosopa clavatus	Nicaragua	León	Telica	12.603333	-86.843056	Occurrence Download https://doi.org/10.15468/dl.3bxkun
Dioprosopa clavatus	Nicaragua	León	León	12.435833	-86.882225	Occurrence Download https://doi.org/10.15468/dl.3bxkun
Dioprosopa clavatus	Costa Rica	Guanacaste	Mansión	10.151995	-85.357177	Occurrence Download https://doi.org/10.15468/dl.3bxkun
Dioprosopa clavatus	Costa Rica	Guanacaste	Santa Cecilia	11.022456	-85.425922	Occurrence Download https://doi.org/10.15468/dl.3bxkun
Dioprosopa clavatus	Costa Rica	Puntarenas	Sabalito	8.891405	-82.764751	Occurrence Download https://doi.org/10.15468/dl.3bxkun
Dioprosopa clavatus	Costa Rica	Guanacaste	Mayorga	10.928721	-85.472248	Occurrence Download https://doi.org/10.15468/dl.3bxkun
Dioprosopa clavatus	Puerto Rico	Puerto Rico	Guajataca	18.391003	-66.911086	Occurrence Download https://doi.org/10.15468/dl.3bxkun
Dioprosopa clavatus	Brazil	Rio Grande do Sul	Barão de Cotegipe	-27.6208	-52.3797	Occurrence Download https://doi.org/10.15468/dl.3bxkun
Dioprosopa clavatus	Brazil	Paraná	Jundiaí do Sul	-23.4367	-50.2475	Occurrence Download https://doi.org/10.15468/dl.3bxkun
Dioprosopa clavatus	United States of America	Texas	Austin	30.18	-97.725	Occurrence Download https://doi.org/10.15468/dl.3bxkun
Dioprosopa clavatus	Brazil	Santa Catarina	Seara	-27.1833	-52.3833	Occurrence Download https://doi.org/10.15468/dl.3bxkun
Dioprosopa clavatus	United States of America	California	Ventura County	34.11	-119.11	Occurrence Download https://doi.org/10.15468/dl.3bxkun
Dioprosopa clavatus	United States of America	Illinois	Mason County	40.4114	-89.8662	Occurrence Download https://doi.org/10.15468/dl.3bxkun
Dioprosopa clavatus	United States of America	Illinois	Massac County	37.2841	-88.8592	Occurrence Download https://doi.org/10.15468/dl.3bxkun
Dioprosopa clavatus	United States of America	Texas	Dimmit County	28.344563	-99.416489	Occurrence Download https://doi.org/10.15468/dl.3bxkun
Dioprosopa clavatus	United States of America	Texas	San Patricio County	28.113226	-97.417747	Occurrence Download https://doi.org/10.15468/dl.3bxkun
Dioprosopa clavatus	Brazil	Paraná	Carambeí	-24.9178	-50.0972	Occurrence Download https://doi.org/10.15468/dl.3bxkun
Dioprosopa clavatus	Mexico	Nuevo León	Linares	24.74729	-99.782057	Occurrence Download https://doi.org/10.15468/dl.3bxkun
Dioprosopa clavatus	United States of America	Florida	Archbold	27.181438	-81.352017	Occurrence Download https://doi.org/10.15468/dl.3bxkun
Dioprosopa clavatus	United States of America	Texas	Yoakum County	33.318209	-102.819016	Occurrence Download https://doi.org/10.15468/dl.3bxkun
Dioprosopa clavatus	Brazil	Espírito Santo	Conceição da Barra	-18.5933	-39.7322	Occurrence Download https://doi.org/10.15468/dl.3bxkun
Dioprosopa clavatus	Brazil	Espírito Santo	Santa Teresa	-19.8167	-40.6778	Occurrence Download https://doi.org/10.15468/dl.3bxkun
Dioprosopa clavatus	United States of America	Georgia	Clinch County	30.914947	-82.706237	Occurrence Download https://doi.org/10.15468/dl.3bxkun
Dioprosopa clavatus	United States of America	Illinois	Farina	38.828764	-88.785179	Occurrence Download https://doi.org/10.15468/dl.3bxkun
Dioprosopa clavatus	United States of America	California	Los Angeles County	34.47	-118.02	Occurrence Download https://doi.org/10.15468/dl.3bxkun

Dioprosopa clavatus	Brazil	Espírito Santo	Santa Teresa	-19.9356	-40.6003	Occurrence Download https://doi.org/10.15468/dl.3bxkun
Dioprosopa clavatus	Brazil	Bahia	Maracás	-13.4411	-40.4308	Occurrence Download https://doi.org/10.15468/dl.3bxkun
Dioprosopa clavatus	Mexico	Durango	Durango	23.978655	-104.783788	Occurrence Download https://doi.org/10.15468/dl.3bxkun
Dioprosopa clavatus	Mexico	Sinaloa	La Guayanera	23.410878	-105.899037	Occurrence Download https://doi.org/10.15468/dl.3bxkun
Dioprosopa clavatus	Brazil	Santa Catarina	Seara	-27.183333	-52.383333	Occurrence Download https://doi.org/10.15468/dl.3bxkun
Dioprosopa clavatus	Brazil	São Paulo	Corumbataí	-22.22	-47.6258	Occurrence Download https://doi.org/10.15468/dl.3bxkun
Dioprosopa clavatus	Brazil	Tocantins	Lagoa da Confusão	-11.5667	-50.6667	Occurrence Download https://doi.org/10.15468/dl.3bxkun
Dioprosopa clavatus	Brazil	Minas Gerais	Ibiraci	-20.4622	-47.1222	Occurrence Download https://doi.org/10.15468/dl.3bxkun
Dioprosopa clavatus	Brazil	Bahia	Caculé	-14.5033	-42.2222	Occurrence Download https://doi.org/10.15468/dl.3bxkun
Dioprosopa clavatus	Brazil	Mato Grosso do Sul	Três Lagoas	-20.7511	-51.6783	Occurrence Download https://doi.org/10.15468/dl.3bxkun
Dioprosopa clavatus	Brazil	Mato Grosso	Comodoro	-13.0875	-59.8958	Occurrence Download https://doi.org/10.15468/dl.3bxkun
Dioprosopa clavatus		Rio de Janeiro	Mangaratiba	-22.9597	-44.0406	Occurrence Download https://doi.org/10.15468/dl.3bxkun
Dioprosopa clavatus	Brazil	Tocantins	Conceição do Araguaia	-8.2578	-49.2647	Occurrence Download https://doi.org/10.15468/dl.3bxkun
Dioprosopa clavatus	United States of America	Florida	Okeechobee	27.243935	-80.829783	Occurrence Download https://doi.org/10.15468/dl.3bxkun
Dioprosopa clavatus	Mexico	Morelos	Yautepec	18.918613	-99.05956	Occurrence Download https://doi.org/10.15468/dl.3bxkun
Dioprosopa clavatus	United States of America	Texas	Motley County	34.071887	-100.791158	Occurrence Download https://doi.org/10.15468/dl.3bxkun
Dioprosopa clavatus	United States of America	California	Los Angeles County	34.2	-117.76	Occurrence Download https://doi.org/10.15468/dl.3bxkun
Dioprosopa clavatus	United States of America	California	Riverside County	33.49	-115.79	Occurrence Download https://doi.org/10.15468/dl.3bxkun
Dioprosopa clavatus	United States of America	California	Blythe	33.61	-114.59	Occurrence Download https://doi.org/10.15468/dl.3bxkun
Dioprosopa clavatus	United States of America	California	Pine Valley	32.82144	-116.529184	Occurrence Download https://doi.org/10.15468/dl.3bxkun
Dioprosopa clavatus	United States of America	South Carolina	Pickens County	34.67	-82.84	Occurrence Download https://doi.org/10.15468/dl.3bxkun
Dioprosopa clavatus	United States of America	Texas	College Station	30.584244	-96.291148	Occurrence Download https://doi.org/10.15468/dl.3bxkun
Dioprosopa clavatus	Venezuela	Lara	Quebrada Seca	9.8206	-69.6183	Occurrence Download https://doi.org/10.15468/dl.3bxkun
Dioprosopa clavatus	Suriname	Galibi	Galibi	5.795833	-54.007778	Reemer 2010
Dioprosopa clavatus	Colombia	Antioquia	El Popo	6.371942	-75.109771	Montoya 2016
Dioprosopa clavatus	Colombia	Antioquia	Guarne	6.295271	-75.411851	Montoya 2016
Dioprosopa clavatus	Colombia	Antioquia	Toruro	6.574793	-75.531269	Montoya 2016
Dioprosopa clavatus	Colombia	Antioquia	Santo Domingo	6.506194	-75.064161	Montoya 2016
Dioprosopa clavatus	Colombia	Caquetá	Florencia	1.616667	-75.666667	Parada-Marín et al. 2025
Dioprosopa clavatus	Suriname	Para	Zuid	5.466111	-55.229722	Reemer 2010
Dioprosopa clavatus	Panama	Colón	Santa Isabel	9.470078	-79.226579	Occurrence Download https://doi.org/10.15468/dl.3bxkun
Dioprosopa clavatus	Panama	Panama	Caimitillo	9.224654	-79.503061	Occurrence Download https://doi.org/10.15468/dl.3bxkun
Dioprosopa clavatus	Panama	Colón	Piña	9.246856	-80.051899	Riccardi et al. 2022
Dioprosopa clavatus	Ecuador	Galápagos	Cantón Santa Cruz	-0.630916	-90.335999	Occurrence Download https://doi.org/10.15468/dl.3bxkun
Dioprosopa clavatus	Ecuador	Galápagos	Cantón Santa Cruz	-0.215313	-90.759382	Occurrence Download https://doi.org/10.15468/dl.3bxkun
Dioprosopa clavatus	Ecuador	Galápagos	Cantón Isabela	-0.12363	-91.367342	Riccardi et al. 2022
Dioprosopa clavatus	Ecuador	Galápagos	Cantón Isabela	-0.907047	-90.923957	Occurrence Download https://doi.org/10.15468/dl.3bxkun
Dioprosopa clavatus	Peru	Ucayali	Callería	-8.005071	-74.1324	Riccardi et al. 2022
Dioprosopa clavatus	Peru	Loreto	Vargas Guerra	-7.111555	-75.004741	Occurrence Download https://doi.org/10.15468/dl.3bxkun

Dioprosopa clavatus	Peru	Loreto	Pampa Hermosa	-7.070043	-75.052685	Riccardi et al. 2022
Dioprosopa clavatus	Peru	Vaqueria	Vaqueria	-8.962096	-77.554288	Occurrence Download https://doi.org/10.15468/dl.3bxkun

Supplementary File S2. Environmental predictors initially considered for the ecological niche models of *Dioprosopa clavata*, including 19 bioclimatic variables from WorldClim 2.1 (BIO1–BIO19) and three physiographic variables (elevation, compound topographic index – CTI, and profile curvature – Pcurv), with their definitions and data sources.

**Climate variables: (WorldClim https://www.worldclim.org/data/worldclim21.html)**
BIO1 = Annual Mean Temperature
BIO2 = Mean Diurnal Range (Mean of monthly (max temp – min temp))
BIO3 = Isothermality (BIO2/BIO7) (×100)
BIO4 = Temperature Seasonality (standard deviation ×100)
BIO5 = Max Temperature of Warmest Month
BIO6 = Min Temperature of Coldest Month
BIO7 = Temperature Annual Range (BIO5–BIO6)
BIO8 = Mean Temperature of Wettest Quarter
BIO9 = Mean Temperature of Driest Quarter
BIO10 = Mean Temperature of Warmest Quarter
BIO11 = Mean Temperature of Coldest Quarter
BIO12 = Annual Precipitation
BIO13 = Precipitation of Wettest Month
BIO14 = Precipitation of Driest Month
BIO15 = Precipitation Seasonality (Coefficient of Variation)
BIO16 = Precipitation of Wettest Quarter
BIO17 = Precipitation of Driest Quarter
BIO18 = Precipitation of Warmest Quarter
BIO19 = Precipitation of Coldest Quarter
**Physiographic variables**
Elevation (https://www.worldclim.org/data/worldclim21.html)
CTI = Compound topographic index (https://www.nature.com/articles/s41597-020-0479-6)
Pcurv = Profile curvature (https://www.nature.com/articles/s41597-020-0479-6)

Supplementary File S3. Variable contributions (%) to habitat suitability models for *Dioprosopa clavata* fitted with three algorithms (GLM, DOM and MXD) and their weighted ensemble. GLM contributions aggregate linear and quadratic terms per predictor. Climatic variables are coded following WorldClim v2.1 (BIO1–BIO19), and physiographic predictors include CTI (Compound Topographic Index), Pcurv (Profile curvature) and elevation. *The ensemble includes only models meeting the performance criterion TSS ≥ 0.70.*

**Variable**	**GLM (TSS ≥ 0.70)**	**DOM (TSS ≥ 0.70)**	**MXD (TSS < 0.70)**	**Ensemble**
CTI — Compound Topographic Index	7.50%	12.05%	5.22%	**9.78%**
Pcurv — Profile curvature	4.60%	5.88%	2.81%	**5.24%**
BIO13 — Precipitation of Wettest Month	5.20%	7.17%	11.00%	**6.18%**
BIO14 — Precipitation of Driest Month	9.90%	0.15%	3.37%	**5.02%**
BIO15 — Precipitation Seasonality (CV)	8.20%	10.04%	9.45%	**9.12%**

BIO18 — Precipitation of Warmest Quarter	3.50%	1.48%	3.38%	**2.49%**
BIO19 — Precipitation of Coldest Quarter	9.00%	7.87%	5.84%	**8.44%**
BIO2 — Mean Diurnal Range	9.10%	0.26%	4.48%	**4.68%**
BIO3 — Isothermality (BIO2/BIO7 ×100)	12.40%	19.96%	32.07%	**16.18%**
BIO8 — Mean Temperature of Wettest Quarter	11.70%	11.68%	2.47%	**11.69%**
BIO9 — Mean Temperature of Driest Quarter	13.40%	1.65%	10.95%	**7.53%**
Elevation	5.60%	21.82%	8.98%	**13.71%**

Supplementary File S4. Mobility-Oriented Parity (MOP) maps used to assess analog versus extrapolative environmental conditions for present and future projections of *Dioprosopa clavata*. Green areas indicate analog conditions within the projected range (MOP ≥ 0.05), whereas red areas indicate high extrapolation in strongly novel environmental space (MOP < 0.05). Only scenarios with no high-extrapolation pixels (i.e., all projected cells with MOP ≥ 0.05) were retained for the analyses described in the main text.

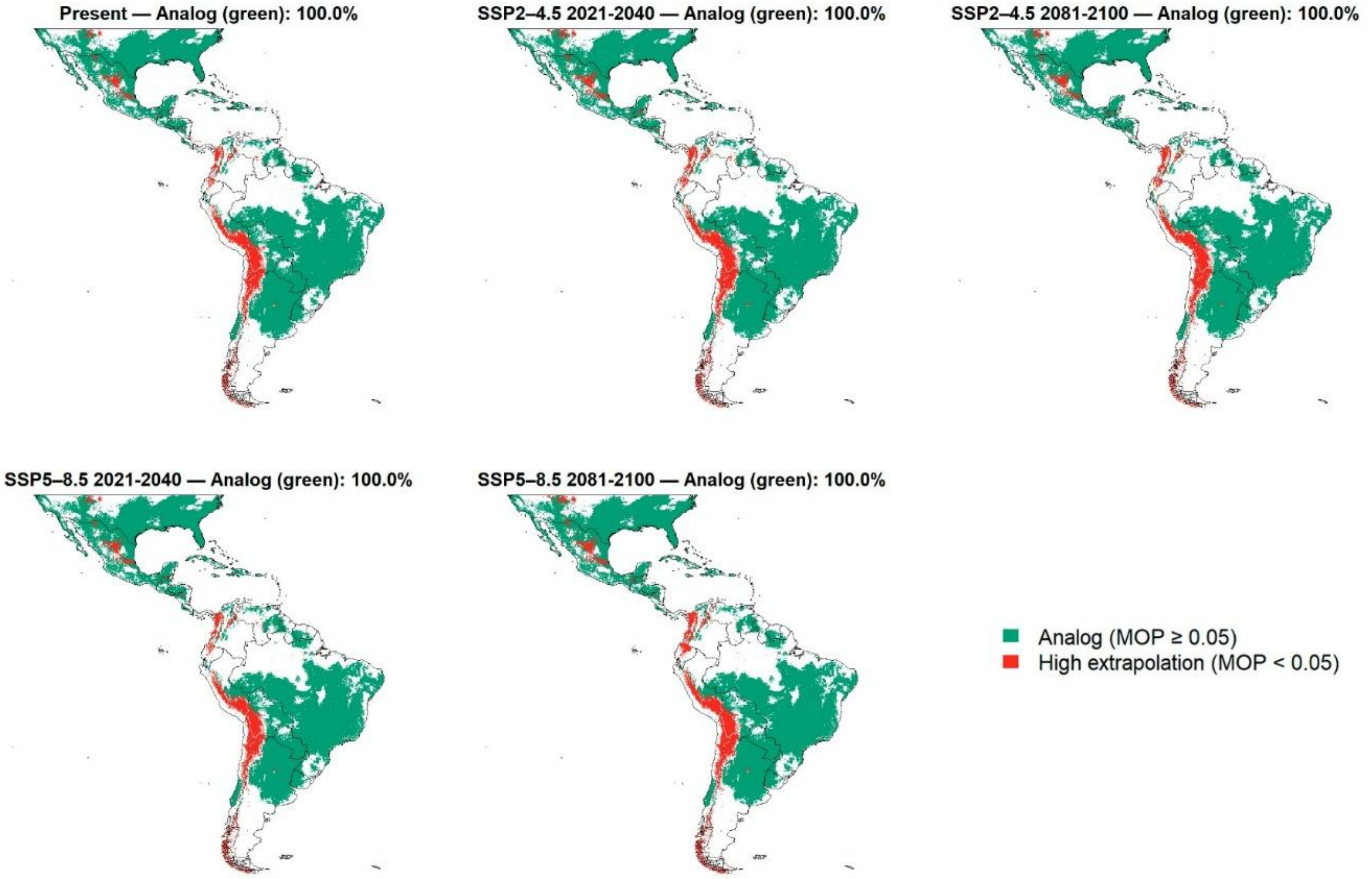

Supplementary File S5. Additional performance metrics for ecological niche models of *Dioprosopa clavata*, including AUC, Kappa, Jaccard, and the corresponding cross-validation standard deviations.

**Algorithm**	**AUC_mean±SD**	**Kappa_mean±SD**	**Jaccard_mean±SD**
DOM	0.928 ± 0.006	0.702 ± 0.016	0.756 ± 0.011
GLM	0.953 ± 0.005	0.797 ± 0.013	0.810 ± 0.012
MXD	0.931 ± 0.009	0.697 ± 0.043	0.748 ± 0.023
SUP	0.954 ± 0.004	0.798 ± 0.012	0.812 ± 0.010
